# Synthesis of Fluorescent Dibenzofuran α-Amino Acids: Conformationally Rigid Analogues of Tyrosine

**DOI:** 10.1021/acs.orglett.5c00433

**Published:** 2025-03-03

**Authors:** Liyao Zeng, Olivia Marshall, Rochelle McGrory, Rebecca Clarke, Ryan J. Brown, Malcolm Kadodwala, Andrew R. Thomson, Andrew Sutherland

**Affiliations:** School of Chemistry, The Joseph Black Building, University of Glasgow, Glasgow G12 8QQ, United Kingdom

## Abstract

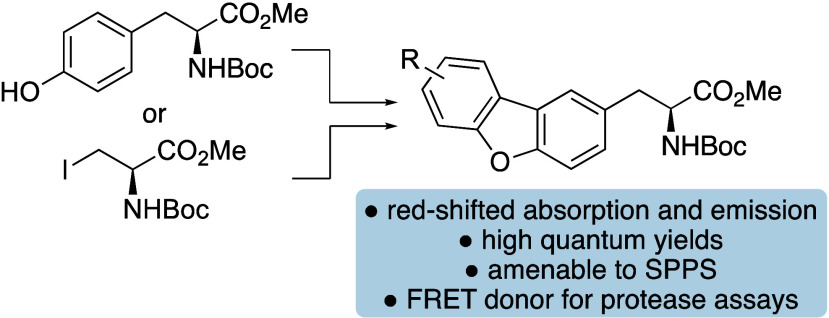

We report two synthetic strategies for the preparation of dibenzofuran α-amino acids, expanding the structural toolbox of fluorescent probes. The strategies involved dibenzofuran synthesis via a Pd(II)-catalyzed C–O cyclization, alongside an efficient Negishi coupling approach for faster access to analogues. These rigid tyrosine mimics possess enhanced fluorescent properties compared to proteinogenic amino acids as demonstrated by application of the lead compound as a FRET donor for monitoring peptide hydrolysis by a serine protease.

α-Amino acids are the key structural components of peptides and proteins and are crucial for a wide range of biological processes.^1^ This importance has resulted in the development of various classes of amino acid analogues, which are widely used for biomedical and life science research. In medicinal chemistry, these are used as enzyme inhibitors, while in biological chemistry they are commonly utilized as probes to investigate protein–protein interactions, protein conformations, and biological mechanisms.^2^ In biological chemistry, fluorescent unnatural amino acids are increasingly used in place of large extrinsic fluorophores that typically require a linker and are positioned at the termini of peptides to minimize disruption of conformation and function.^3^ Fluorescent unnatural amino acids which are generally smaller, can be selectively embedded within proteins and peptides using genetic encoding or solid phase peptide synthesis (SPPS), allowing retention of structure and more localized investigation of the biological environment.

A key strategy for the discovery of novel fluorescent peptide probes is the modification of proteinogenic α-amino acids.^3^ While many studies have focused on extending the conjugation of l-tryptophan,^4^ which has the strongest brightness, fluorescent analogues of l-tyrosine (**1**) have also been developed (Figure 1a). For example, Mely and co-workers synthesized a series of flavone-derived α-amino acids (**2**) from l-tyrosine (**1**).^5^ These were shown to possess dual emission fluorescence and were incorporated into peptides to study interactions with oligonucleotides and lipid bilayers. Other studies have utilized a copper-catalyzed C–O bond coupling reaction to attach polyaromatic groups to l-tyrosine.^6^ Incorporation of moieties such as pyrene and biphenyl generated fluorophores (**3**) with good quantum yields (0.18–0.40) and emission in the UV region. The Heck reaction has also been used to extend the conjugation of l-tyrosine (**1**).^7^ Coupling of diiodo-l-tyrosine with styrenes produced environment sensitive, dialkenyl tyrosine analogues such as **4**, that was incorporated into a cell penetrating peptide and used to monitor cell internalization.

**Figure 1:**
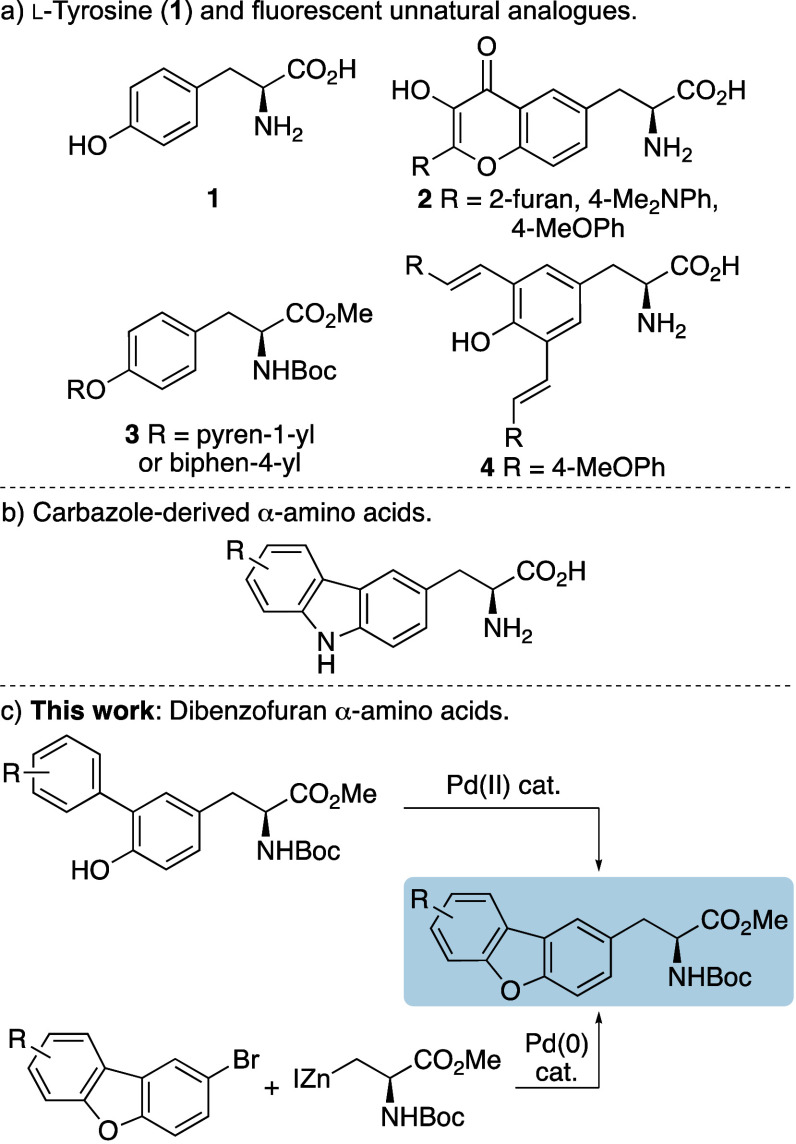
a) l-Tyrosine and fluorescent analogues. b) Carbazole α-amino acids. c) This work.

We have developed a program of research focused on the discovery of fluorescent unnatural α-amino acids and recently reported the synthesis and photophysical properties of carbazole-derived α-amino acids (Figure 1b).^8^ These were designed as structural mimics of tryptophan, which was confirmed by their replacement of tryptophan in peptides with retention of conformation. The lead carbazole-derived α-amino acid was incorporated into a proline-rich peptide ligand and its fluorescent properties were used to measure binding to the WW domain protein. As the oxygen analogue of carbazoles, dibenzofurans have not been previously explored as an amino acid fluorophore,^9^ we were interested in accessing these and assessing their potential for biological applications. We now report the synthesis of dibenzofuran α-amino acids using two approaches involving *O*-arylation of tyrosine and then palladium-catalyzed C–O cyclization or Negishi coupling of 3-iodoalanine with halogenated dibenzofurans (Figure 1c). We also describe their photophysical properties, the compatibility of the lead compound with SPPS and its application as a FRET donor within a decapeptide to monitor enzyme activity.

The first stage of this project focused on the development of an effective synthetic strategy that would allow access to various dibenzofuran α-amino acid analogues.^10^ The first approach involved the *ortho*-arylation of tyrosine, followed by Pd(II)-catalyzed C(sp^2^)–H activation and C–O cyclization (Figure 1c). To access *ortho*-aryl analogues of tyrosine for cyclization, a five-step route was developed. Initially, commercially available tyrosine derivative **5** was brominated using *N*-bromosuccinimide (NBS) and catalytic *p*-tosic acid (Scheme 1).^11^ This gave monobrominated tyrosine **6** in 79% yield. Attempted Suzuki-Miyaura coupling of **6** with various aryl boronic acids gave low yields and returned tyrosine **5**.^12^ It was proposed that this was due to proto-depalladation involving the hydroxyl group. Thus, the hydroxyl group was protected using the methoxymethyl (MOM) group under standard conditions and in quantitative yield. Various Pd-catalysts and ligands were then screened for the subsequent Suzuki-Miyaura reaction with aryl boronic acids. The most effective was the Buchwald group XPhos Pd G3 precatalyst, which allowed fast coupling under mild conditions and access to coupled products (**8a**–**c**) in excellent yields.^13^ The phenol required for the key cyclization step was then deprotected under acidic conditions, which required reprotection of the amine. This gave the cyclization precursors **9a**–**c** in 89–93% yields over the two steps. Various copper- and palladium-catalyzed methods have been reported for the preparation of dibenzofurans via C(sp^2^)–H activation and C–O cyclization.^14^ Procedures by the groups of Liu and Zhu gave the products in good yields, however, these methods required high temperatures (120–140 °C).^14a,14c^ For an approach that would be compatible with α-amino acids, we chose to investigate the method by Wei and Yoshikai involving Pd(OAc)_2_, 3-nitropyridine as the ligand and *tert*-butyl peroxybenzoate as the oxidant.^14b^ Although moderate yielding, this method could be performed at lower temperatures (90 °C). Cyclization of *ortho*-aryl tyrosine analogues **9a**–**c** using this method, which is proposed to proceed via a Pd(II)/Pd(IV) catalytic cycle was investigated. At 90 °C, a reaction time of 18 h was found to be optimal, allowing the isolation of highly conjugated (**10a**), electron-rich (**10b**) and electron-deficient (**10c**) analogues in 33–52% yields.

**Scheme 1:**
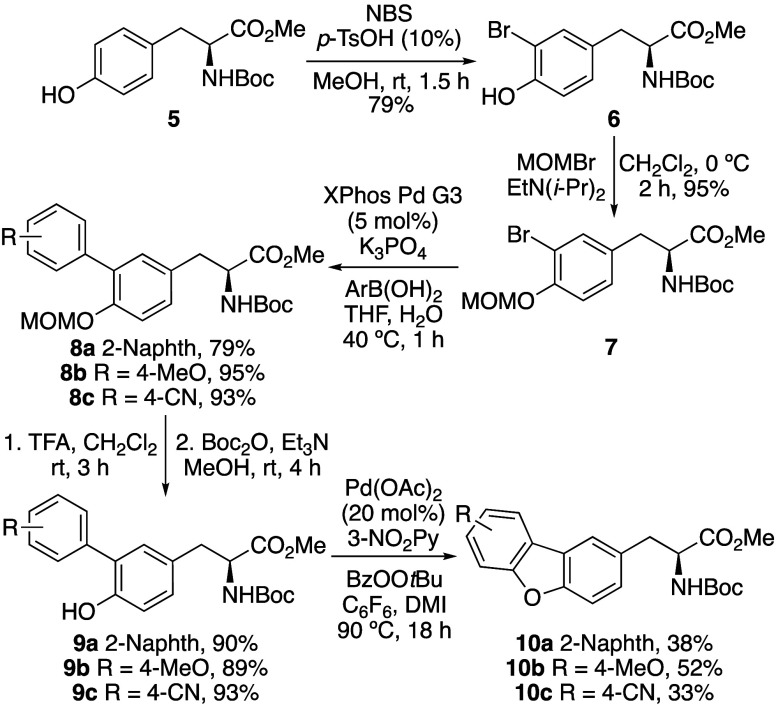
Synthesis of Dibenzofuran α-Amino Acids **10a**–**c**

Although this approach permitted access to **10a**–**c** in acceptable overall yields (20–33%), the six-step route and moderate-yielding cyclization restricted access to other targets. For these reasons, a second approach was investigated involving the Negishi coupling of brominated dibenzofurans with an organozinc reagent derived from 3-iodoalanine (Figure 1c). Jackson and co-workers demonstrated that the coupling of this β-alanine anion reagent with halogenated (hetero)arenes was an effective approach for the preparation of unnatural amino acids.^15^ Initially, 3-iodoalanine **12** was prepared from serine derivative **11** by mesylation and then iodination under standard conditions (Scheme 2).^16^ This gave 3-iodoalanine in 67% yield over the two steps. Organozinc reagent **13** was generated by treatment with iodine-activated zinc dust.^8,15c^ Subsequent palladium-catalyzed coupling with various bromodibenzofurans using SPhos^17^ as a ligand gave dibenzofuran α-amino acids **10a**, **10d** and **10e** in 44–62% yields. Overall, this three-pot approach resulted in an improved synthesis of **10a** and rapid access to two further analogues.

**Scheme 2:**
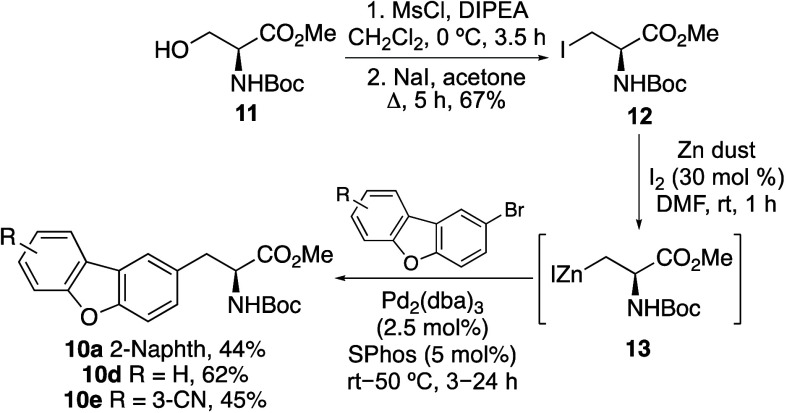
Synthesis of α-Amino Acids **10a**, **10d** and **10e**

The photophysical properties of dibenzofuran α-amino acids **10a**–**e** were then measured (Table 1).^18,19^ As expected, these more conjugated and rigid fluorophores displayed bathochromic shifts of absorption and emission (Figure 2) compared to tyrosine **1**, along with significantly enhanced brightness. For example, highly conjugated naphthobenzofuran **10a** was found to have the most red-shifted absorption and emission and an overall brightness of 12100 cm^–1^ M^–1^. Dibenzofuran α-amino acids bearing electron-rich (**10b**), electron-deficient (**10c**) or no substituent (**10d**) were found to have excellent quantum yields (0.49–0.62), resulting in good brightness for relatively small fluorophores. As **10b** was found to possess the highest quantum yield and brightness, this amino acid was investigated further. Initially **10b** was deprotected to confirm that the parent amino acid retained the favorable photophysical properties. The ester was hydrolyzed using LiOH and the Boc-group was removed using trifluoroacetic acid (TFA) (Scheme 3). On recrystallization, this gave **14** in 95% overall yield and measurement of the optical properties showed similar results to **10b** (Table 1). Solvatochromic and pH studies were then conducted using **14**.^18^ Surprisingly, **14** showed minimal sensitivity to polarity. Although the intensity of the main absorption and emission bands varied from nonpolar to polar solvents, these appeared at similar wavelengths. For example, the emission maximum in THF was found at 317 nm compared to 327 nm in water. pH studies also demonstrated that the absorption and emission properties of amino acid **14** were insensitive to acidic or basic conditions.

**Figure 2:**
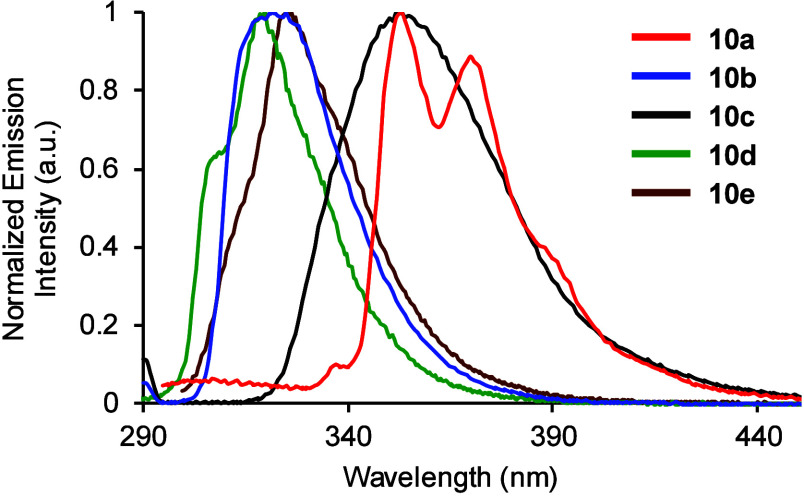
Normalized emission spectra of **10a**–**10e** (1.25–5 μM in MeOH).

**Scheme 3:**
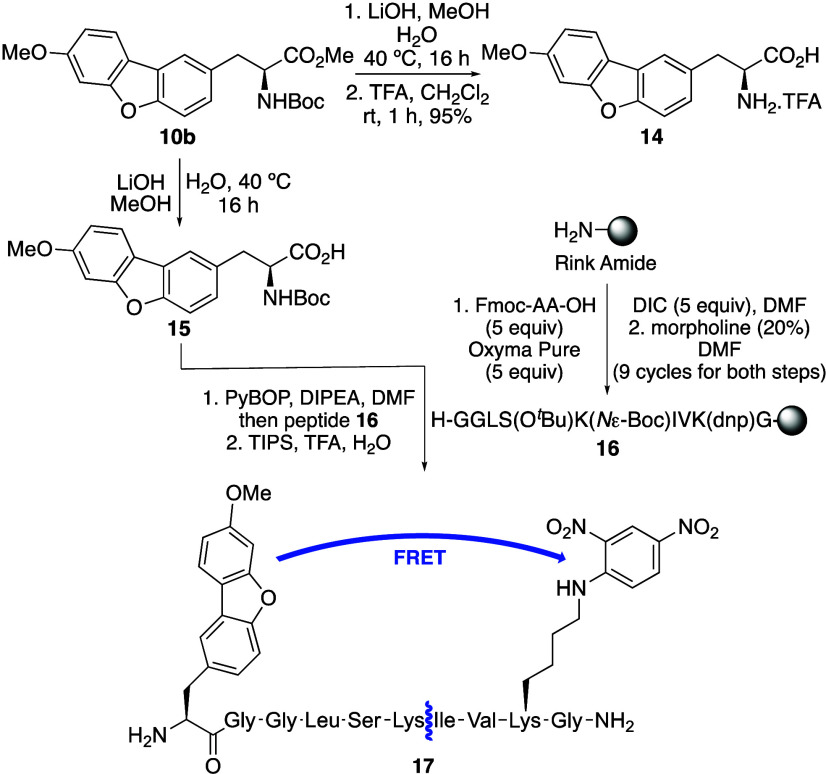
Synthesis of Amino Acid **14** and Peptide **17**

**Table 1:** Photophysical Data of α-Amino Acids^18^

amino acid	λ_Abs_ (nm)^a^	ε (cm^–1^ M^–1^)	λ_Em_ (nm)^a^	Φ_F_^b^	brightness (cm^–1^ M^–1^)
**1**	275	1410	310	0.14	197
**10a**	320	78000	353, 370	0.16	12100
**10b**	306	23100	323	0.62	14300
**10c**	295	21600	353	0.49	10600
**10d**	286	15800	319	0.60	9480
**10e**	290	10200	325	0.40	4110
**14**	307	25947	325	0.57	14791

a Spectra were recorded at 1.25–5 μM in MeOH.

b Quantum yields (Φ_F_) were determined using l-trp as the standard.

As amino acid **14** demonstrated strong fluorescence and photophysical properties that are insensitive to the environment, it was proposed that this could be used as a general probe for measurement of biological processes. Fluorophores attached to peptide substrates are commonly used as Förster resonance energy transfer (FRET) pairs to monitor and evaluate enzyme kinetics.^20^ For example, Poreba and co-workers showed internally quenched fluorescent decapeptides with a N-terminal coumarin donor and a 2,4-dinitrophenyl-lysine acceptor could be used to probe the substrate specificity of protease enzymes.^21^ Based on this work, we believed that amino acid **14** could also act as a donor with 2,4-DNP-lysine. Investigation of their photophysical properties showed excellent overlap of the emission band of **14** with the absorption band of 2,4-DNP-lysine (Figure 3a). Thus, a proof-of-concept study was designed to incorporate amino acid **14** into a peptide containing 2,4-DNP-lysine and to use this to probe the substrate specificity of the serine protease, trypsin.^22,23^ Initially nonapeptide **16** was prepared using Rink Amide resin as the polymer support and routine SPPS methodology (Scheme 3). Coupling of each Fmoc-protected amino acid was performed using *N*,*N*′-diisopropylcarbodiimide (DIC)/OxymaPure activation, followed by morpholine-mediated *N*-deprotection. As well as incorporation of the 2,4-DNP-lysine acceptor, the nonapeptide also included a lysine residue, a known cleavage site of trypsin.^22^ Boc-protected amino acid **15** was then coupled manually and on deprotection and release from the resin using a TFA cocktail, decapeptide **17** was purified using reverse-phase HPLC and characterized by mass spectrometry. Excitation of **17** at 290 nm showed only 3% retention of emission compared to the cleaved decapeptide,^18^ thus confirming effective energy transfer to the 2,4-DNP-lysine acceptor. The Förster distance (*R*_0_), which is the distance of 50% energy transfer between a donor and acceptor, was calculated for the FRET pair and shown to be 34.20 Å.^19^ This value is similar to other commonly used FRET pairs.^19,21^ Decapeptide **17** was then confirmed as a substrate of trypsin. Treatment of the peptide with the serine protease resulted in gradual restoration of emission intensity as a function of time (Figure 3b and 3c). Thus, dibenzofuran α-amino acid **14** was found to be compatible with SPPS methods^24^ and as part of an internally quenched peptide, its fluorescence could be used to monitor the activity of trypsin-mediated hydrolytic digestion.

**Figure 3:**
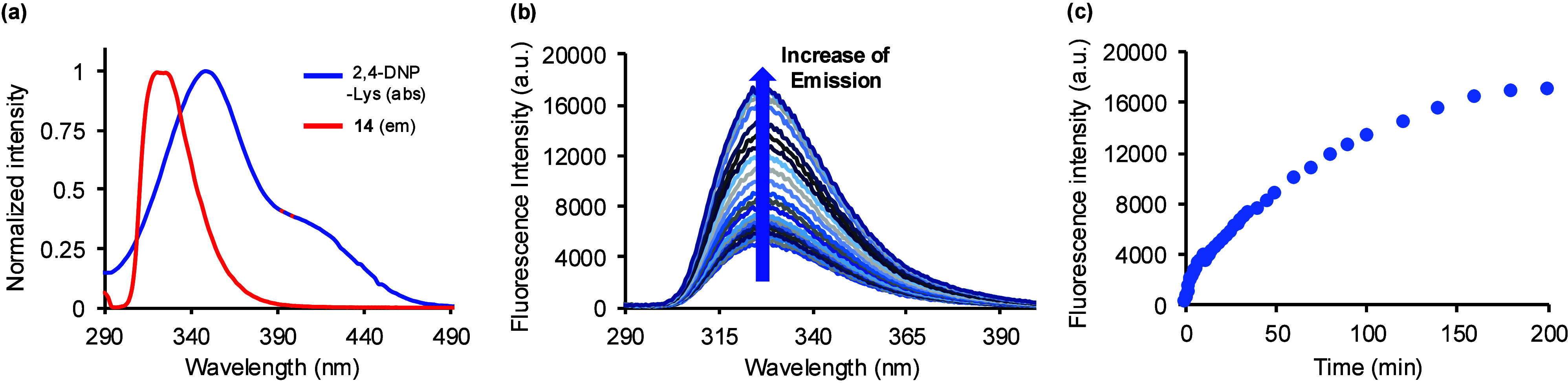
(a) Overlap of absorption of 2,4-DNP-lysine (blue) and emission of amino acid **14** (red). (b) Increase of emission during trypsin cleavage of decapeptide **17**. (c) Emission versus time during trypsin cleavage of **17**. The enzyme hydrolysis reaction used peptide **17** (10 μM) and trypsin (0.02 μM) in 3-morpholinopropanesulfonic acid (MOPS) buffer (20 mM) at pH 7.0.

In summary, this study presents synthetic strategies for the preparation of novel dibenzofuran α-amino acids, using Pd(II)-catalyzed C(sp^2^)–H activation and C–O cyclization, complemented by an optimized Negishi coupling approach. While the Pd(II)-catalyzed approach allowed access to dibenzofuran amino acids in good overall yields (20–33%), the Negishi coupling strategy provided a streamlined alternative with fewer steps and improved overall efficiency. These structurally rigid fluorophores expand the scope of unnatural amino acid chemistry, providing new tools for peptide-based optical probes as demonstrated by the application of the lead compound as a FRET donor for monitoring hydrolytic digestion of the serine protease, trypsin. Future work will explore the application of this methodology for the discovery of additional dibenzofuran analogues and in combination with SPPS will investigate these as components of fluorescent peptide probes for novel biological chemistry applications.

## Data Availability

The data underlying this study are available in the published article and its Supporting Information.

## Supporting Information Available

Experimental procedures, characterization data, photophysical data, and NMR spectra of all compounds (PDF)
