# Supplementary material for: Synthesis of Fluorescent Dibenzofuran α-Amino Acids: Conformationally Rigid Analogues of Tyrosine
Source: Org Lett. 2025 Mar 3;27(10):2475–9. doi: 10.1021/acs.orglett.5c00433 (PMC11915488; doi:10.1021/acs.orglett.5c00433)

Supporting Information for:

Synthesis of Fluorescent Dibenzofuran α-Amino Acids: Conformationally Rigid Analogues of Tyrosine

Liyao Zeng, Olivia Marshall, Rochelle McGrory, Rebecca Clarke, Ryan J. Brown,

*Malcolm Kadodwala, Andrew R. Thomson and Andrew Sutherland**

[†]School of Chemistry, The Joseph Black Building, University of Glasgow, Glasgow G12 8QQ, United Kingdom.

Table of Contents

1. General Experimental S2
2. Experimental Procedures and Spectroscopic Data for all Compounds S2–S14
3. Photophysical Data for α-Amino Acids **10a**–**e** and **14** S15–S18
4. Determination of Förster Distance for Amino acid **14**/Lysine(dnp) Pair and Reaction of Decapeptide **17** with Trypsin S19–S21
5. References S22
6. ^{1}H and ^{13}C NMR Spectra for all Novel Compounds S23–S52

1. General Experimental

All reagents and starting materials were obtained from commercial sources and used as received. Reactions were performed open to air unless otherwise mentioned. All reactions performed at elevated temperatures were heated using an oil bath. Brine refers to a saturated aqueous solution of sodium chloride. Flash column chromatography was performed using silica gel 60 (40–63 μm). Aluminium-backed plates pre-coated with silica gel $60F_{254}$ were used for thin layer chromatography and were visualized with a UV lamp or by staining with potassium permanganate, vanillin or ninhydrin. ^{1}H NMR spectra were recorded on a NMR spectrometer at either 400 or 500 MHz and data are reported as follows: chemical shift in ppm relative to the solvent as internal standard ($CHCl_3$, δ 7.26 ppm; CH_3OH, δ 3.31 ppm; DMSO, δ 2.50), multiplicity (s = singlet, d = doublet, t = triplet, q = quartet, m = multiplet or overlap of non-equivalent resonances, integration). ^{13}C NMR spectra were recorded on a NMR spectrometer at either 101 or 126 MHz and data are reported as follows: chemical shift in ppm relative to tetramethylsilane or the solvent as internal standard ($CDCl_3$, δ 77.2 ppm; CD_3OD, δ 49.0 ppm; DMSO-d_6, δ 39.5), multiplicity with respect to hydrogen (deduced from DEPT experiments, C, CH, CH_2 or CH_3). Infrared spectra were recorded on a FTIR spectrometer; wavenumbers are indicated in cm^{-1}. Mass spectra were recorded using electrospray techniques. HRMS spectra were recorded using quadrupole time of flight (Q-TOF) mass spectrometers. Melting points are uncorrected. Optical rotations were determined as solutions irradiating with the sodium D line (λ = 589 nm) using a polarimeter. $[\alpha]_D$ values are given in units 10^{-1} deg cm^{-1} g^{-1}. UV-Vis and fluorescence spectra were recorded on a fluorescence and absorbance spectrometer. Absorbance spectra were recorded with an integration time of 0.05 s and a band pass of 5 nm. Fluorescence spectra were recorded with excitation and emission band pass of 5 nm, an integration time of 2 s, and with detector accumulations set to 1. Quantum yield data were measured using anthracene and L-tryptophan as standard references.

2. Experimental Procedures and Spectroscopic Data for all Compounds

Methyl (2*S*)-2-[(*tert*-butoxycarbonyl)amino]-3-(3’-bromo-4’-hydroxyphenyl)propanoate (6)[1]

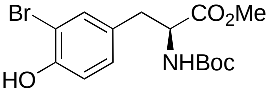

To a stirred solution of methyl (2*S*)-2-[(*tert*-butoxycarbonyl)amino]-3-(4-hydroxyphenyl)propanoate (**5**) (0.670 g, 2.27 mmol) and *p*-toluenesulfonic acid (0.0840 g, 0.227 mmol, 10 mol %) in methanol (2 mL) in a foiled round bottomed flask was added a solution of *N*-bromosuccinimide (0.444 g, 2.50 mmol) in methanol (20 mL) dropwise from a foiled dropping funnel over 0.3 h. The reaction mixture was

stirred at room temperature for 3 h. The reaction mixture was concentrated *in vacuo*. Purification by flash column chromatography, eluting with 5% ethyl acetate in dichloromethane gave methyl (2*S*)-2-[(*tert*-butoxycarbonyl)amino]-3-(3'-bromo-4'-hydroxyphenyl)propanoate (**6**) (0.673 g, 79%) as a white solid. Mp 111–115 °C (lit.[1] 117–119 °C); $[\alpha]_D^{21}$ +45.7 (*c* 0.1, $CHCl_3$); ^{1}H NMR (400 MHz, $CDCl_3$) δ 7.23 (d, *J* = 1.5 Hz, 1H), 7.03–6.90 (m, 2H), 5.43 (s, 1H), 4.98 (d, *J* = 8.2 Hz, 1H), 4.57–4.47 (m, 1H), 3.73 (s, 3H), 3.05 (dd, *J* = 14.0, 5.2 Hz, 1H), 2.95 (dd, *J* = 14.0, 5.9 Hz, 1H), 1.43 (s, 9H); ^{13}C{^{1}H} NMR (101 MHz, $CDCl_3$) δ 172.2, 155.1, 151.5, 132.8, 130.2, 129.9, 116.2, 110.3, 80.2, 54.6, 52.5, 37.4, 28.4; MS (ESI) *m/z*: [M + Na]$^+$ Calcd for $C_{15}H_{20}{}^{79}BrNO_5Na$ 396.05; Found 396.04.

Methyl (2*S*)-3-[3'-bromo-4'-(methoxymethoxy)phenyl]-2-[(*tert*-butoxycarbonyl)amino]propanoate (7)

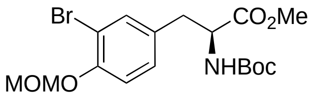

To a stirred solution of methyl (2*S*)-2-[(*tert*-butoxycarbonyl)amino]-3-(3'-bromo-4'-hydroxyphenyl)propanoate (**6**) (4.74 g, 12.7 mmol) in dichloromethane (120 mL) at 0 °C was added *N*,*N*-diisopropylethylamine (9.35 mL, 50.6 mmol) and bromomethyl methyl ether (3.69 mL, 44.3 mmol). The reaction mixture was stirred at 0 °C for 0.2 h, then warmed to room temperature and stirred for 2 h. The reaction mixture was diluted in dichloromethane (100 mL) and washed with 0.5 M citric acid solution (2 × 100 mL), sodium bicarbonate (2 × 80 mL) and brine (2 × 80 mL). The organic layer was dried ($MgSO_4$), filtered and concentrated *in vacuo.* Purification by flash column chromatography, eluting with 20% ethyl acetate in hexane gave methyl (2*S*)-3-[3'-bromo-4'-(methoxymethoxy)phenyl]-2-[(*tert*-butoxycarbonyl)amino]propanoate (**7**) (5.05 g, 95%) as a colorless oil. IR (neat) 3364, 2978, 1746, 1713, 1495, 1366, 1246, 1161, 1045 cm^{-1}; $[\alpha]_D^{22}$ +36.8 (*c* 0.1, $CHCl_3$); ^{1}H NMR (400 MHz, $CDCl_3$) δ 7.30 (d, *J* = 2.0 Hz, 1H), 7.07 (d, *J* = 8.4 Hz, 1H), 7.00 (dd, *J* = 8.4, 2.0 Hz, 1H), 5.22 (s, 2H), 4.99 (d, *J* = 8.2 Hz, 1H), 4.57–4.49 (m, 1H), 3.73 (s, 3H), 3.51 (s, 3H), 3.06 (dd, *J* = 14.0, 5.6 Hz, 1H), 2.96 (dd, *J* = 14.0, 6.0 Hz, 1H), 1.43 (s, 9H); ^{13}C{^{1}H} NMR (101 MHz, $CDCl_3$) δ 172.2, 155.1, 153.0, 134.3, 131.2, 129.4, 116.3, 112.9, 95.3, 80.2, 56.5, 54.5, 52.5, 37.3, 28.4; HRMS (ESI-TOF) *m/z*: [M + Na]$^+$ Calcd for $C_{17}H_{24}{}^{79}BrNO_6Na$ 440.0679; Found 440.0677.

General Procedure for the Synthesis of 3'-Aryl Tyrosine Analogues: A solution of methyl (2*S*)-3-[3'-bromo-4'-(methoxymethoxy)phenyl]-2-[(*tert*-butoxycarbonyl)amino]propanoate (**7**) (1 equiv.), boronic acid (1.5 equiv.) and potassium phosphate (2 equiv.) in tetrahydrofuran (0.1 mL per mmol) and water (0.1 mL per mmol) was degassed under argon for 0.2 h. To this was added XPhos Pd G3 (5 mol

%). The reaction mixture was stirred at 40 °C for 1–2 h. The reaction mixture was cooled to room temperature and concentrated *in vacuo.* The reaction mixture was diluted in water (10 mL) and extracted with ethyl acetate (3 × 10 mL). The organic layer was dried ($MgSO_4$), filtered and concentrated *in vacuo.* Purification by flash column chromatography gave the desired products.

Methyl (2*S*)-2-[(*tert*-butoxycarbonyl)amino]-3-[3'-(naphth-2''-yl)-4'-(methoxymethoxy)phenyl]propanoate (8a)

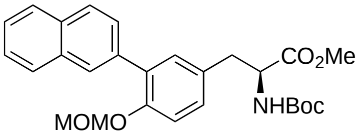

Methyl (2*S*)-2-[(*tert*-butoxycarbonyl)amino]-3-[3'-(naphth-2''-yl)-4'-(methoxymethoxy)phenyl]propanoate (**8a**) was synthesized as described in the general procedure using methyl (2*S*)-3-[3'-bromo-4'-(methoxymethoxy)phenyl]-2-[(*tert*-butoxycarbonyl)amino]propanoate (**7**) (0.300 g, 0.717 mmol), 2-naphthalene boronic acid (0.185 g, 1.08 mmol), XPhos Pd G3 (0.0150 g, 0.0177 mmol, 2.5 mol %) and potassium phosphate (0.304 g, 1.43 mmol) in tetrahydrofuran/water (1:1, 9 mL) at 40 °C for 0.5 h. To this was added additional XPhos Pd G3 (0.0150 g, 0.0177 mmol, 2.5 mol %). The reaction mixture was stirred at 40 °C for an additional 0.5 h. Purification by flash column chromatography, eluting with 20% ethyl acetate in hexane gave methyl (2*S*)-2-[(*tert*-butoxycarbonyl)amino]-3-[3'-(naphth-2''-yl)-4'-(methoxymethoxy)phenyl]propanoate (**8a**) (0.263 g, 79%) as a colorless oil. IR (neat) 3349, 2974, 1744, 1709, 1497, 1157, 991, 752 cm^{-1}; $[\alpha]_D^{22}$ +27.4 (*c* 0.1, $CHCl_3$); ^1H NMR (400 MHz, $CDCl_3$) δ 7.93 (d, J = 1.8 Hz, 1H), 7.90–7.82 (m, 3H), 7.66 (dd, J = 8.5, 1.8 Hz, 1H), 7.53–7.45 (m, 2H), 7.22–7.14 (m, 2H), 7.08 (dd, J = 8.4, 2.3 Hz, 1H), 5.11 (s, 2H), 5.03 (d, J = 8.4 Hz, 1H), 4.66–4.57 (m, 1H), 3.74 (s, 3H), 3.38 (s, 3H), 3.15 (dd, J = 14.0, 5.6 Hz, 1H), 3.07 (dd, J = 14.0, 5.9 Hz, 1H), 1.41 (s, 9H); $^{13}C\{^1H\}$ NMR (101 MHz, $CDCl_3$) δ 172.5, 155.3, 153.6, 136.2, 133.5, 132.6, 132.3, 132.0, 129.9, 129.6, 128.3, 128.2, 128.1, 127.8, 127.4, 126.2, 126.0, 116.0, 95.3, 80.1, 56.3, 54.6, 52.4, 37.7, 28.5; HRMS (ESI-TOF) *m/z*: $[M + Na]^+$ Calcd for $C_{27}H_{31}NO_6Na$ 488.2044; Found 488.2044.

Methyl (2*S*)-2-[(*tert*-butoxycarbonyl)amino]-3-[3'-(4''-methoxyphenyl)-4'-(methoxymethoxy)phenyl]propanoate (8b)

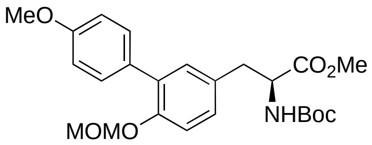

Methyl (2*S*)-2-[(*tert*-butoxycarbonyl)amino]-3-[3'-(4''-methoxyphenyl)-4'-(methoxymethoxy)phenyl]propanoate (**8b**) was synthesized as described in the general procedure using methyl (2*S*)-3-[3'-bromo-4'-(methoxymethoxy)phenyl]-2-[(*tert*-butoxycarbonyl)amino]propanoate (**7**) (0.418 g, 1.00 mmol), 4-methoxyphenylboronic acid (0.228 g, 1.50 mmol), XPhos Pd G3 (0.0420 g, 0.0500 mmol, 5 mol %) and potassium phosphate (0.425 g, 2.00 mmol) in tetrahydrofuran/water (1:1, 8 mL) at 40 °C. The reaction was allowed to stir for 2 h. Purification by flash column chromatography, eluting with 25% ethyl acetate in hexanes gave methyl (2*S*)-2-[(*tert*-butoxycarbonyl)amino]-3-[3'-(4''-methoxyphenyl)-4'-(methoxymethoxy)phenyl]propanoate (**8b**) (0.423 g, 95%) as a colorless oil. IR (neat) 3342, 2954, 2361, 1742, 1712, 1491, 1244, 1160 cm^{-1}; $[\alpha]_D^{22}$ –50.0 (*c* 0.1, $CHCl_3$); ^1H NMR (400 MHz, $CDCl_3$) δ 7.47–7.41 (m, 2H), 7.12 (d, *J* = 8.4 Hz, 1H), 7.05 (d, *J* = 2.2 Hz, 1H), 7.01 (dd, *J* = 8.4, 2.2 Hz, 1H), 6.97–6.91 (m, 2H), 5.09 (s, 2H), 5.02 (d, *J* = 8.4 Hz, 1H), 4.63–4.54 (m, 1H), 3.85 (s, 3H), 3.73 (s, 3H), 3.39 (s, 3H), 3.11 (dd, *J* = 13.9, 5.7 Hz, 1H), 3.03 (dd, *J* = 13.9, 6.0 Hz, 1H), 1.42 (s, 9H); $^{13}C\{^1H\}$ NMR (101 MHz, $CDCl_3$) δ 172.5, 158.8, 155.2, 153.3, 131.9, 131.5, 130.8, 130.6, 129.7, 129.0, 115.8, 113.5, 95.1, 80.0, 56.2, 55.4, 54.5, 52.3, 37.5, 28.4; HRMS (ESI-TOF) *m/z*: $[M + Na]^+$ Calcd for $C_{24}H_{31}NO_7Na$ 468.1993; Found 468.2007.

Methyl (2*S*)-2-[(*tert*-butoxycarbonyl)amino]-3-[3'-(4''-cyanophenyl)-4'-(methoxymethoxy)phenyl]propanoate (8c)

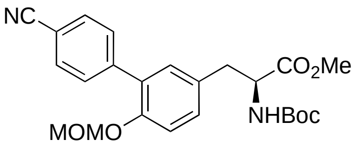

Methyl (2*S*)-2-[(*tert*-butoxycarbonyl)amino]-3-[3'-(4''-cyanophenyl)-4'-(methoxymethoxy)phenyl]propanoate (**8c**) was synthesized as described in the general procedure using methyl (2*S*)-3-[3'-bromo-4'-(methoxymethoxy)phenyl]-2-[(*tert*-butoxycarbonyl)amino]propanoate (**7**) (0.131 g, 0.313 mmol), 4-cyanophenylboronic acid (0.0690 g, 0.470 mmol), XPhos Pd G3 (0.0130 g, 0.0154 mmol, 5 mol %) and potassium phosphate (0.133 g, 0.626 mmol) in tetrahydrofuran/water (1:1,

2 mL) at 40 °C. The reaction was allowed to stir for 2 h. Purification by flash column chromatography, eluting with 2.5% ethyl acetate in dichloromethane gave methyl (2*S*)-2-[(*tert*-butoxycarbonyl)amino]-3-[3'-(4''-cyanophenyl)-4'-(methoxymethoxy)phenyl]propanoate (**8c**) (0.128 g, 93%) as a colorless oil. IR (neat) 3376, 2978, 2226, 1744, 1709, 1493, 1157, 991, 841, 752 cm^{-1}; $[\alpha]_D^{20}$ +27.3 (*c* 0.1, $CHCl_3$); ^{1}H NMR (400 MHz, $CDCl_3$) δ 7.71–7.66 (m, 2H), 7.64–7.58 (m, 2H), 7.15 (d, *J* = 8.5 Hz, 1H), 7.09 (dd, *J* = 8.5, 2.2 Hz, 1H), 7.05 (d, *J* = 2.2 Hz, 1H), 5.12 (s, 2H), 5.01 (d, *J* = 8.4 Hz, 1H), 4.64–4.54 (m, 1H), 3.73 (s, 3H), 3.38 (s, 3H), 3.14 (dd, *J* = 13.9, 5.7 Hz, 1H), 3.02 (dd, *J* = 13.9, 6.1 Hz, 1H), 1.40 (s, 9H); ^{13}C{^{1}H} NMR (101 MHz, $CDCl_3$) δ 172.4, 155.1, 153.3, 143.3, 131.9, 131.8, 130.8, 130.3, 130.1, 129.8, 119.2, 115.7, 110.8, 95.1, 80.2, 56.4, 54.5, 52.5, 37.6, 28.4; HRMS (ESI-TOF) *m/z*: $[M + Na]^+$ Calcd for $C_{24}H_{28}N_2O_6Na$ 463.1840; Found 463.1849.

Methyl (2*S*)-2-[(*tert*-butoxycarbonyl)amino]-3-[3'-(naphth-2''-yl)-4'-hydroxyphenyl]propanoate (9a)

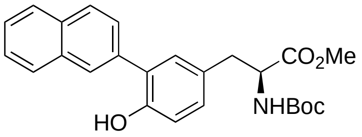

Methyl (2*S*)-2-[(*tert*-butoxycarbonyl)amino]-3-[3'-(naphth-2''-yl)-4'-(methoxymethoxy)phenyl]propanoate (**8a**) (0.315 g, 0.676 mmol) was dissolved in dichloromethane (5 mL) and cooled to 0 °C. Trifluoroacetic acid (1.5 mL) was added dropwise. The reaction mixture was warmed to room temperature and stirred for 3 h. Concentration *in vacuo* gave methyl (2*S*)-2-amino-3-[3'-(naphtha-2''-yl)-4'-hydroxyphenyl]propanoate trifluoroacetate (0.285 g) as an off-white solid. To a solution of methyl (2*S*)-2-amino-3-[3'-(naphtha-2''-yl)-4'-hydroxyphenyl]propanoate trifluoroacetate (0.285 g, 0.681 mmol) in methanol (3 mL) at 0 °C was added triethylamine (0.283 mL, 2.04 mmol) and di-*tert*-butyl dicarbonate (0.164 g, 0.749 mmol). The reaction mixture was stirred at 0 °C for 0.2 h before warming to room temperature and stirring for 4 h. The reaction mixture was concentrated *in vacuo*. Purification by flash column chromatography, eluting with 2% ethyl acetate in dichloromethane gave methyl (2*S*)-2-[(*tert*-butoxycarbonyl)amino]-3-[3'-(naphth-2''-yl)-4'-hydroxyphenyl]propanoate (**9a**) (0.258 g, 90%) as a white solid. Mp 63–65 °C; IR (neat) 3341, 2972, 2362, 1717, 1679, 1504, 1364, 1280, 1252, 1160, 822 cm^{-1}; $[\alpha]_D^{21}$ +46.4 (*c* 0.1, $CHCl_3$); ^{1}H NMR (400 MHz, $CDCl_3$) δ 7.96 (d, *J* = 8.5 Hz, 1H), 7.94–7.82 (m, 3H), 7.60–7.50 (m, 3H), 7.09 (d, *J* = 2.3 Hz, 1H), 7.04 (dd, *J* = 8.2, 2.3 Hz, 1H), 6.95 (d, *J* = 8.2 Hz, 1H), 5.36 (br s, 1H), 5.03 (d, *J* = 8.5 Hz, 1H), 4.65–4.54 (m, 1H), 3.73 (s, 3H), 3.12 (dd, *J* = 14.0, 5.7 Hz, 1H), 3.05 (dd, *J* = 14.0, 5.7 Hz, 1H), 1.41 (s, 9H); ^{13}C{^{1}H} NMR (101 MHz, $CDCl_3$) δ 172.6, 155.3, 151.9, 134.5, 133.7, 132.9, 131.5, 130.1, 129.3, 128.4, 128.19, 128.16, 127.9,

127.2, 126.8, 126.6, 116.2, 80.1, 54.7, 52.4, 37.7, 28.5; HRMS (ESI-TOF) *m/z*: $[M - H]^-$ Calcd for $C_{25}H_{26}NO_5$ 420.1816; Found 420.1817.

Methyl (2*S*)-2-[(*tert*-butoxycarbonyl)amino]-3-[3'-(4''-methoxyphenyl)-4'-hydroxyphenyl]propanoate (9b)

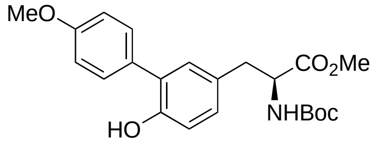

The reaction was conducted as described for **9a** using methyl (2*S*)-2-[(*tert*-butoxycarbonyl)amino]-3-[3'-(4''-methoxyphenyl)-4'-(methoxymethoxy)phenyl]propanoate (**8b**) (0.401 g, 0.800 mmol). Purification by flash column chromatography, eluting with 25% ethyl acetate in hexanes gave methyl (2*S*)-2-[(*tert*-butoxycarbonyl)amino]-3-[3'-(4''-methoxyphenyl)-4'-hydroxyphenyl]propanoate (**9b**) (0.323 g, 89%) as a white solid. Mp 43–44 °C; IR (neat) 3365, 2974, 2361, 1685, 1502, 1364, 1240, 1160, 753 cm^{-1}; $[\alpha]_D^{22}$ –160.0 (*c* 0.1, $CHCl_3$); ^1H NMR (400 MHz, $CDCl_3$) δ 7.42–7.35 (m, 2H), 7.02–6.92 (m, 4H), 6.89–6.83 (m, 1H), 5.65 (s, 1H), 5.04 (d, *J* = 8.4 Hz, 1H), 4.56 (dt, *J* = 8.4, 5.8 Hz, 1H), 3.84 (s, 3H), 3.71 (s, 3H), 3.07 (dd, *J* = 13.9, 5.8 Hz, 1H), 3.00 (dd, *J* = 14.1, 5.8 Hz, 1H), 1.41 (s, 9H); $^{13}C\{^1H\}$ NMR (101 MHz, $CDCl_3$) δ 172.6, 159.3, 155.3, 151.9, 131.3, 130.3, 129.5, 129.4, 128.0, 116.0, 114.6, 80.1, 55.4, 54.6, 52.4, 37.5, 28.4; HRMS (ESI-TOF) *m/z*: $[M + Na]^+$ Calcd for $C_{22}H_{27}NO_6Na$ 424.1731: Found 424.1747.

Methyl (2*S*)-2-[(*tert*-butoxycarbonyl)amino]-3-[3'-(4''-cyanophenyl)-4'-hydroxyphenyl]propanoate (9c)

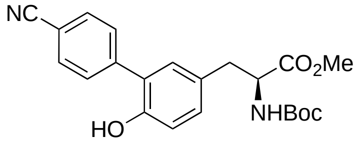

The reaction was conducted as described for **9a** using methyl (2*S*)-2-[(*tert*-butoxycarbonyl)amino]-3-[3'-(4''-cyanophenyl)-4'-(methoxymethoxy)phenyl]propanoate (**8c**) (0.210 g, 0.470 mmol). Purification by flash column chromatography, eluting with 25% ethyl acetate in hexanes gave methyl (2*S*)-2-[(*tert*-butoxycarbonyl)amino]-3-[3'-(4''-cyanophenyl)-4'-hydroxyphenyl]propanoate (**9c**) (0.173 g, 93%) as a white solid. Mp 66–67 °C; IR (neat) 3354, 2981, 2226, 1683, 1605, 1499, 1365, 1161, 842 cm^{-1}; $[\alpha]_D^{22}$ +20.0 (*c* 0.1, $CHCl_3$); ^1H NMR (400 MHz, $CDCl_3$) δ 7.67 (d, *J* = 8.2 Hz, 2H), 7.63 (d, *J* = 8.2 Hz, 2H), 7.06–6.89 (m, 2H), 6.81 (d, *J* = 8.1 Hz, 1H), 6.48 (s, 1H), 5.08 (d, *J* = 8.4 Hz, 1H), 4.60–4.51 (m, 1H), 3.71 (s, 3H), 3.09 (dd, *J* = 14.0, 5.8 Hz, 1H), 2.98 (dd, *J* = 14.0, 6.2 Hz, 1H),

1.39 (s, 9H); $^{13}C\{^1H\}$ NMR (101 MHz, $CDCl_3$) δ 172.6, 155.4, 152.3, 142.9, 132.2, 131.4, 130.7, 130.0, 128.4, 126.6, 119.0, 116.7, 110.7, 80.5, 54.6, 52.5, 37.6, 28.4; HRMS (ESI-TOF) *m/z*: $[M + H]^+$ Calcd for $C_{22}H_{24}N_2O_5H$ 397.1758; Found 397.1762.

Methyl (2*S*)-2-[(*tert*-butoxycarbonyl)amino]-3-(3'-naphtho[2,3-b]benzofuran)propanoate (10a)

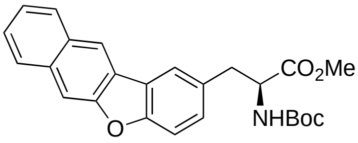

A solution of methyl (2*S*)-2-[(*tert*-butoxycarbonyl)amino]-3-[3'-(naphth-2''-yl)-4'-hydroxyphenyl]propanoate (**9a**) (0.15 g, 0.35 mmol), palladium acetate (0.016 g, 0.070 mmol, 20 mol %) and 3-nitropyridine (0.0090 g, 0.070 mmol, 20 mol %) in hexafluorobenzene (0.45 mL) and *N*,*N*-dimethylimidazolidinone (0.3 mL) was degassed under argon for 0.2 h. To this was added *tert*-butyl peroxybenzoate (0.13 mL, 0.70 mmol). The reaction was heated to 90 °C and stirred for 18 h. The reaction mixture was cooled to room temperature. Purification by flash column chromatography, eluting with 25% ethyl acetate in hexane gave methyl (2*S*)-2-[(*tert*-butoxycarbonyl)amino]-3-(3'-naphtho[2,3-b]benzofuran)propanoate (**10a**) (0.057 g, 38%) as a yellow solid. Mp 120–124 °C; IR (neat) 3357, 2921, 2358, 1700, 1504, 1481, 1394, 1242, 1167, 747 cm^{-1}; $[\alpha]_D^{15}$ +67.2 (*c* 0.1, $CHCl_3$); ^1H NMR (400 MHz, $CDCl_3$) δ 8.34 (s, 1H), 8.05–8.00 (m, 1H), 7.98–7.93 (m, 1H), 7.89 (s, 1H), 7.82 (d, *J* = 1.8 Hz, 1H), 7.55–7.45 (m, 3H), 7.26 (dd, *J* = 8.1, 1.8 Hz, 1H), 5.11 (d, *J* = 8.3 Hz, 1H), 4.73–4.65 (m, 1H), 3.75 (s, 3H), 3.31 (dd, *J* = 14.0, 5.9 Hz, 1H), 3.23 (dd, *J* = 14.0, 6.2 Hz, 1H), 1.44 (s, 9H); $^{13}C\{^1H\}$ NMR (101 MHz, $CDCl_3$) δ 172.5, 156.9, 155.2, 133.2, 130.8, 130.2, 129.5, 128.5, 127.9, 126.0, 125.3, 124.4, 124.3, 122.0, 119.2, 111.6, 107.1, 80.1, 55.0, 52.4, 38.4, 28.4; HRMS (ESI-TOF) *m/z*: $[M + Na]^+$ Calcd for $C_{25}H_{25}NO_5Na$ 442.1625; Found 442.1639.

Methyl (2*S*)-2-[(*tert*-butoxycarbonyl)amino]-3-(7-methoxydibenzo[b,d]furan-2-yl)propanoate (10b)

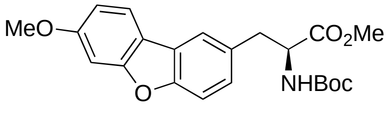

The reaction was conducted as described for **10a** using methyl (2*S*)-2-[(*tert*-butoxycarbonyl)amino]-3-[3'-(4''-methoxyphenyl)-4'-hydroxyphenyl]propanoate (**9b**) (0.040 g, 0.10 mmol). Purification by flash column chromatography, eluting with 25% ethyl acetate in hexanes gave methyl (2*S*)-2-[(*tert*-butoxycarbonyl)amino]-3-(7-methoxydibenzo[b,d]furan-2-yl)propanoate (**10b**) (0.021 g, 52%) as a

white solid. Mp 127–128 °C; IR (neat) 3338, 2932, 2359, 1737, 1688, 1496, 1278, 1164, 1144, 811 cm^{-1}; $[\alpha]_D^{22}$ –190.0 (*c* 0.1, $CHCl_3$); ^1H NMR (400 MHz, $CDCl_3$) δ 7.76 (d, *J* = 8.5 Hz, 1H), 7.64–7.58 (m, 1H), 7.43 (d, *J* = 8.5 Hz, 1H), 7.12 (d, *J* = 8.5 Hz, 1H), 7.07 (d, *J* = 2.3 Hz, 1H), 6.93 (dd, *J* = 8.5, 2.3 Hz, 1H), 5.05 (d, *J* = 8.3 Hz, 1H), 4.67–4.59 (m, 1H), 3.89 (s, 3H), 3.72 (s, 3H), 3.26 (dd, *J* = 13.9, 6.0 Hz, 1H), 3.19 (dd, *J* = 13.9, 6.2 Hz, 1H), 1.41 (s, 9H); $^{13}C\{^1H\}$ NMR (101 MHz, $CDCl_3$) δ 172.5, 160.1, 158.0, 155.6, 155.2, 130.6, 126.8, 124.8, 121.0, 120.5, 117.2, 111.4, 111.1, 96.6, 80.1, 55.8, 55.0, 52.4, 38.4, 28.4; HRMS (ESI-TOF) *m/z*: $[M + Na]^+$ Calcd for $C_{22}H_{25}NO_6Na$ 422.1574; Found 422.1586.

Methyl (2*S*)-2-[(*tert*-butoxycarbonyl)amino]-3-(7-cyanodibenzo[b,d]furan-2-yl)propanoate (10c)

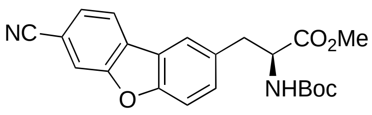

The reaction was conducted as described for **10a** using methyl (2*S*)-2-[(*tert*-butoxycarbonyl)amino]-3-[3’-(4’’-cyanophenyl)-4’-hydroxyphenyl]propanoate (**9c**) (0.210 g, 0.10 mmol). Purification by flash column chromatography, eluting with 25% ethyl acetate in hexanes gave methyl (2*S*)-2-[(*tert*-butoxycarbonyl)amino]-3-(7-cyanodibenzo[b,d]furan-2-yl)propanoate (**10c**) (0.0689 g, 33%) as a white solid. Mp 132–133 °C; IR (neat) 3354, 2986, 2357, 2227, 1734, 1683, 1523, 1251, 1162, 1016, 822 cm^{-1}; $[\alpha]_D^{22}$ –30.0 (*c* 0.1, $CHCl_3$); ^1H NMR (400 MHz, $CDCl_3$) δ 7.98 (d, *J* = 8.0 Hz, 1H), 7.85 (s, 1H), 7.77 (s, 1H), 7.62 (d, *J* = 8.0 Hz, 1H), 7.54 (d, *J* = 8.5 Hz, 1H), 7.33 (d, *J* = 8.5 Hz, 1H), 5.06 (d, *J* = 8.2 Hz, 1H), 4.69–4.61 (m, 1H), 3.73 (s, 3H), 3.31 (dd, *J* = 14.1, 5.9 Hz, 1H), 3.20 (dd, *J* = 14.1, 6.2 Hz, 1H), 1.40 (s, 9H); $^{13}C\{^1H\}$ NMR (101 MHz, $CDCl_3$) δ 172.3, 156.5, 155.3, 155.1, 131.9, 130.6, 128.5, 126.7, 123.1, 122.1, 121.5, 119.1, 115.8, 112.2, 109.9, 80.2, 54.9, 52.4, 38.4, 28.4; HRMS (ESI-TOF) *m/z*: $[M + Na]^+$ Calcd for $C_{22}H_{22}N_2O_5Na$ 417.1421; Found 417.1439.

Methyl (2*R*)-2-[(*tert*-butoxycarbonyl)amino]-3-iodopropanoate (12)[2]

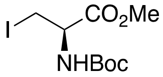

Methyl (2*S*)-2-[(*tert*-butoxycarbonyl)amino]-3-hydroxypropanoate (**11**) (0.624 g, 2.85 mmol) was dissolved in dry dichloromethane (12 mL) and cooled to 0 °C. Diisopropylethylamine (0.550 mL, 3.13 mmol) was added, followed by dropwise addition of methanesulfonyl chloride (0.240 mL, 3.13 mmol). The reaction mixture was stirred for 3.5 h at 0 °C. The reaction quenched with water (12 mL). The product was extracted with dichloromethane (2 × 100 mL) and washed with brine (100 mL). The solvent

was removed under reduced pressure to give methyl (2*S*)-2-[(*tert*-butoxycarbonyl)amino]-3-(methylsulfonyloxy)propanoate as a colorless oil. The crude methyl (2*S*)-2-[(*tert*-butoxycarbonyl)amino]-3-(methylsulfonyloxy)propanoate product was subsequently dissolved in acetone (18 mL). Sodium iodide (0.855 g, 5.70 mmol) was added. The reaction mixture was heated to 60 °C and stirred for 3 h. The mixture was cooled and filtered and the filtrate concentrated *in vacuo*. Purification by flash column chromatography, eluting with 20% ethyl acetate in hexane gave methyl (2*R*)-2-[(*tert*-butoxycarbonyl)amino]-3-iodopropanoate (**12**) (0.628 g, 67%) as a yellow solid. Mp 43–45 °C; $[\alpha]_D^{24}$ +37.4 (*c* 1.0, $CHCl_3$) [lit.[2] +40.3 (*c* 1.0, $CHCl_3$)]; ^{1}H NMR (400 MHz, $CDCl_3$) δ 5.35 (br d, J = 7.0 Hz, 1H), 4.54–4.48 (m, 1H), 3.78 (s, 3H), 3.58 (dd, J = 10.4, 3.8 Hz, 1H), 3.54 (dd, J = 10.4, 4.1 Hz, 1H), 1.44 (s, 9H); ^{13}C{^{1}H} NMR (101 MHz, $CDCl_3$) δ 170.2, 155.0, 80.5, 53.8, 53.1, 28.4, 7.9; MS (ESI) *m/z*: $[M + Na]^+$ Calcd for $C_9H_{16}INO_4Na$ 352.01; Found 352.01.

Methyl (2*S*)-2-[(*tert*-butoxycarbonyl)amino]-3-(3'-naptho[2,3-b]benzofuran)propanoate (10a)

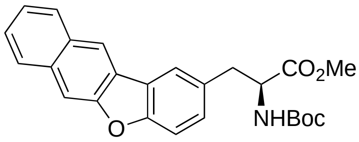

To an oven-dried Young's flask under an inert atmosphere was added zinc dust (0.0908 g, 1.39 mmol) and iodine (0.0179 g, 0.0705 mmol). Methyl (2*R*)-2-[(*tert*-butoxycarbonyl)amino]-3-iodopropanoate (**12**) (0.150 g, 0.456 mmol) was added as a solution in dry *N*,*N*'-dimethylformamide (1.0 mL). A further portion of iodine was added (0.0184 g, 0.0725 mmol) and a colour change from yellow to colorless was observed, along with a noticeable exotherm. The reaction mixture was stirred at room temperature for 1 h. Tris(dibenzylideneacetone)dipalladium(0)-chloroform adduct (0.0124 g, 0.0120 mmol), 2-dicyclohexylphosphino-2′,6′-dimethoxybiphenyl (0.00990 g, 0.0241 mmol) and 2-bromonaphtho[2,3-b]benzofuran (0.176 g, 0.593 mmol) were added to the reaction mixture. A further 1.5 mL of *N*,*N*'-dimethylformamide was added and the reaction stirred at 40 °C for 3.5 h. The reaction mixture was diluted with ethyl acetate (50 mL) and washed with an aqueous lithium chloride solution (5% w/w, 2 × 50 mL). The organic layer was dried ($MgSO_4$) and concentrated under reduced pressure. Purification by flash column chromatography, eluting with 15% ethyl acetate in hexane gave methyl (2*S*)-2-[(*tert*-butoxycarbonyl)amino]-3-(3'-naptho[2,3-b]benzofuran)propanoate (**10a**) (0.0844 g, 44%) as a yellow solid. Physical properties and spectroscopic data as described above.

Methyl (2*S*)-2-[(*tert*-butoxycarbonyl)amino]-3-(dibenzo[b,d]furan-2′-yl)propanoate (10d)

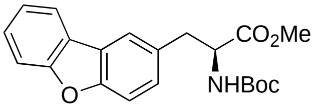

Methyl (2*S*)-2-[(*tert*-butoxycarbonyl)amino]-3-(dibenzo[b,d]furan-2′-yl)propanoate (**10d**) was synthesized as described for methyl (2*S*)-2-[(*tert*-butoxycarbonyl)amino]-3-(3'-naptho[2,3-b]benzofuran)propanoate (**10a**), using zinc dust (0.0899 g, 1.37 mmol), iodine (2 × 0.0170 g, 0.137 mmol), methyl (2*R*)-2-[(*tert*-butoxycarbonyl)amino]-3-iodopropanoate (**12**) (0.150 g, 0.456 mmol), tris(dibenzylideneacetone)dipalladium(0)-chloroform adduct (0.0124 g, 0.0120 mmol), 2-dicyclohexylphosphino-2′,6′-dimethoxybiphenyl (0.00950 g, 0.0231 mmol), and 2-bromodibenzo[b,d]furan (0.147 g, 0.595 mmol) in *N*,*N*'-dimethylformamide (2.5 mL) at 40 °C for 4 h. Purification by flash column chromatography, eluting with 10–20% ethyl acetate in hexane gave methyl (2*S*)-2-[(*tert*-butoxycarbonyl)amino]-3-(dibenzo[b,d]furan-2′-yl)propanoate (**10d**) as an off-white solid (0.104 g, 62%). Mp 100–102 °C; $[\alpha]_D^{18}$ +51.4 (*c* 0.1, $CHCl_3$); IR (neat) 3350, 2934, 2165, 1735, 1688, 1515, 1282, 1192, 1157, 995 cm^{-1}; ^1H NMR (400 MHz, $CDCl_3$) δ 7.92 (d, *J* = 7.6 Hz, 1H), 7.72 (d, *J* = 1.6 Hz, 1H), 7.56 (d, *J* = 8.0 Hz, 1H), 7.52–7.42 (m, 2H), 7.34 (t, *J* = 7.6 Hz, 1H), 7.21 (dd, *J* 8.4, 1.6 Hz, 1H), 5.03 (d, *J* = 7.6 Hz, 1H), 4.69–4.59 (m, 1H), 3.72 (s, 3H), 3.29 (dd, *J* = 14.0, 6.0 Hz, 1H), 3.21 (dd, *J* = 14.0, 6.0 Hz, 1H), 1.42 (s, 9H); $^{13}C\{^1H\}$ NMR (101 MHz, $CDCl_3$) δ 172.5, 156.6, 155.5, 155.2, 130.7, 128.4, 127.4, 124.6, 124.1, 122.9, 121.4, 120.7, 111.9, 111.8, 80.1, 55.0, 52.4, 38.5, 28.4; HRMS (ESI-TOF) *m/z*: $[(MH - CO_2{}^tBu) + H]^+$ Calcd for $C_{16}H_{15}NO_3H$ 270.1125; Found 270.1128.

Methyl (2*S*)-2-[(*tert*-butoxycarbonyl)amino]-3-(8′-cyanodibenzo[b,d]furan-2′-yl)propanoate (10e)

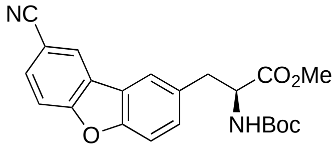

Methyl (2*S*)-2-[(*tert*-butoxycarbonyl)amino]-3-(8′-cyanodibenzo[b,d]furan-2′-yl)propanoate (**10e**) was synthesized as described for methyl (2*S*)-2-[(*tert*-butoxycarbonyl)amino]-3-(3'-naptho[2,3-b]benzofuran)propanoate (**10a**), using zinc dust (0.0586 g, 0.896 mmol), iodine (2 × 0.0115 g, 0.0909 mmol), methyl (2*R*)- 2-[(*tert*-butoxycarbonyl)amino]-3-iodopropanoate (**12**) (0.0997 g, 0.303 mmol), tris(dibenzylideneacetone)dipalladium(0)-chloroform adduct (0.00780 g, 0.00760 mmol), 2-dicyclohexylphosphino-2′,6′-dimethoxybiphenyl (0.0062 g, 0.0152 mmol) and 8-bromodibenzo[b,d]furan-2-carbonitrile (0.108 g, 0.397 mmol) in *N*,*N*'-dimethylformamide (1.0 mL) at

20 °C for 24 h. Purification by flash column chromatography, eluting with 15% ethyl acetate in petroleum ether gave methyl (2*S*)-2-[(*tert*-butoxycarbonyl)amino]-3-(8′-cyanodibenzo[b,d]furan-2′-yl)propanoate (**10e**) as a brown solid (0.0548 g, 45%). Mp 135–138 °C; $[\alpha]_D^{18}$ +49.1 (*c* 0.1, $CHCl_3$); IR (neat) 3340, 2919, 2223, 1735, 1689, 1553, 1483, 1455, 1250, 1157, 822 cm^{-1}; ^1H NMR (400 MHz, $CDCl_3$) δ 8.23 (d, *J* = 2.0 Hz, 1H), 7.76–7.71 (m, 2H), 7.64 (d, *J* = 8.4 Hz, 1H), 7.54 (d, *J* = 8.8 Hz, 1H), 7.32 (dd, *J* = 8.8, 2.0 Hz, 1H), 5.05 (d, *J* = 8.8 Hz, 1H), 4.69–4.60 (m, 1H), 3.74 (s, 3H), 3.32 (dd, *J* 14.0, 5.6 Hz, 1H), 3.20 (dd, *J* = 14.0, 6.0 Hz, 1H), 1.41 (s, 9H); $^{13}C\{^1H\}$ NMR (101 MHz, $CDCl_3$) δ 172.3, 158.4, 156.1, 155.2, 132.1, 131.1, 130.1, 125.5, 125.2, 123.0, 121.8, 119.3, 113.1, 112.2, 106.8, 80.3, 54.9, 52.5, 38.5, 28.4; HRMS (ESI-TOF) *m/z*: $[(MH - CO_2{}^tBu) + H]^+$ Calcd for $C_{17}H_{14}N_2O_3H$ 295.1077; Found 295.1076.

(2*S*)-2-Amino-3-(7-methoxydibenzo[b,d]furan-2-yl)propanoic acid trifluoroacetate (14)

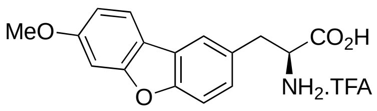

To a stirred solution of methyl (2*S*)-2-[(*tert*-butoxycarbonyl)amino]-3-(7-methoxydibenzo[b,d]furan-2-yl)propanoate (**10b**) (0.040 g, 0.10 mmol) in methanol (3 mL) was added dropwise a solution of lithium hydroxide (0.011 g, 0.25 mmol) in water (1 mL). The reaction mixture was heated to 40 °C and stirred for 16 h. The reaction mixture was cooled to room temperature and concentrated *in vacuo.* The reaction mixture was diluted in water (5 mL), acidified to pH 1 using 1 M aqueous hydrochloric acid and extracted with ethyl acetate (3 × 20 mL). The combined organic layers were dried ($MgSO_4$), filtered and concentrated *in vacuo.* The residue was dissolved in dichloromethane (2 mL) and cooled to 0 °C. Trifluoroacetic acid (0.5 mL) was added dropwise. The reaction mixture was warmed to room temperature and stirred for 1 h. Concentration *in vacuo*, followed by recrystallization from methanol and diethyl ether gave (2*S*)-2-amino-3-(7-methoxydibenzo[b,d]furan-2-yl)propanoic acid trifluoroacetate (**14**) as a white solid (0.036 g, 95%). Mp 202–203 °C; IR (neat) 2932, 2361, 1670, 1635, 1609, 1498, 1430, 1279, 1187, 1142, 799 cm^{-1}; $[\alpha]_D^{22}$ +11.0 (*c* 1.0, MeOH); ^1H NMR (400 MHz, CD_3OD) δ 7.89–7.79 (m, 2H), 7.50 (d, *J* = 8.4 Hz, 1H), 7.30 (dd, *J* = 8.4, 1.9 Hz, 1H), 7.13 (d, *J* = 2.3 Hz, 1H), 6.96 (dd, *J* = 8.6, 2.3 Hz, 1H), 4.31 (dd, *J* = 7.8, 5.4 Hz, 1H), 3.88 (s, 3H), 3.46 (dd, *J* = 14.6, 5.4 Hz, 1H), 3.37–3.24 (m, 2H); $^{13}C\{^1H\}$ NMR (101 MHz, CD_3OD) δ 171.3, 161.9, 159.4, 157.2, 130.2, 128.0, 126.4, 122.1, 121.8, 118.0, 112.7, 112.4, 97.3, 56.2, 55.5, 37.3; HRMS (ESI-TOF) *m/z*: $[M + H]^+$ Calcd for $C_{16}H_{15}NO_4H$ 286.1074; Found 286.1077.

Synthesis of Peptide (16, H-GGLS(O^{t}Bu)K(Nε-Boc)IVK(dnp)G-resin)

Peptide **16** was synthesised on a CEM Liberty Blue peptide synthesis instrument using standard solid phase peptide synthesis (SPPS) protocols, involving Fmoc/^{t}Bu protecting group strategy and DIC/OxymaPure activation chemistry. Resin (0.1 mmol, Rink Amide MBHA, 0.33 mmol/g) was used. After the synthesis was completed, the resin was washed by *N*,*N*'-dimethylformamide (3 × 3 mL) and dichloromethane (3 × 3 mL).

Synthesis of Peptide (17, H-15-GGLSKIVK(dnp)G-NH$_2$)

To a stirred solution of methyl (2*S*)-2-[(*tert*-butoxycarbonyl)amino]-3-(7-methoxydibenzo[b,d]furan-2-yl)propanoate (**10b**) (0.040 g, 0.10 mmol) in methanol (3 mL) was added dropwise a solution of lithium hydroxide (0.011 g, 0.25 mmol) in water (1 mL). The reaction mixture was heated to 40 °C and stirred for 16 h. The reaction mixture was cooled to room temperature and concentrated *in vacuo.* The reaction mixture was diluted in water (5 mL), acidified to pH 1 using 1 M aqueous hydrochloric acid and extracted with ethyl acetate (3 × 20 mL). The combined organic layers were dried ($MgSO_4$), filtered and concentrated *in vacuo*. The resulting carboxylic acid, (2*S*)-2-[(*tert*-butoxycarbonyl)amino]-3-(7-methoxydibenzo[b,d]furan-2-yl)propanoic acid (**15**) (0.029 g, 0.075 mmol) was coupled with peptide **16** (0.05 mmol) using DIPEA (26 µL, 0.15 mmol), PyBOP (0.052 g, 0.10 mmol) in *N*,*N*'-dimethylformamide (1.5 mL) for 2 h. Then the reaction mixture was washed by *N*,*N*'-dimethylformamide (3 × 3 mL) and dichloromethane (3 × 3 mL). Triisopropylsilane (50 µL, 2.5%), water (50 µL, 2.5%) and TFA (1.9 mL, 95%) were added to the resin. The reaction mixture was stirred gently for 2 h and then filtered. The filtrate was evaporated with a flow of nitrogen gas, followed by precipitation of the peptide in cold diethyl ether (50 mL). The precipitate was dissolved in 1:1 H_2O/MeCN and lyophilized to give a yellow powder. Peptide **17** was purified on a Dionex reverse-phase HPLC system equipped with Dionex P680 pumps and a Dionex UVD170U UV–vis detector (monitoring at 214 nm and 280 nm), using a Phenomenex, Gemini, C18, 5 µm, 250 × 21.2 mm column at a flow rate of 8 mL/min. Gradients were run using a binary solvent system consisting of A (H_2O + 0.1% TFA) and B (MeCN + 0.1% TFA). A gradient from 25% to 45% solution B over 0.5 h was used. Collected fractions were lyophilized on a Christ Alpha 2–4 LO plus freeze dryer. LC–MS analysis was performed on a Thermo Scientific LCQ Fleet quadrupole mass spectrometer using positive mode electrospray ionization (ESI+). Gradients were run using a binary solvent system consisting of solution A (95/5 H_2O/MeCN + 0.1% TFA) and B (95/5 MeCN/H_2O + 0.1% TFA). A gradient from 10% to 60% solution B over 20 min was used. MS (ESI) *m/z*: 1290.6 $[M + H]^+$.

Peptide **17** was analyzed on a Shimadzu reverse-phase HPLC system equipped with Shimadzu LC-20AT pumps, a Shimadzu SIL-20A autosampler and a Shimadzu SPD-20A UV–vis detector

(monitoring at 214 and 280 nm) using a Phenomenex, Aeris, 5 μm, peptide XB-C18, 150 × 4.6 mm column at a flow rate of 1 mL/min. Gradients were run using a binary solvent system consisting of solution A (H_2O + 0.1% TFA) and B (MeCN + 0.1% TFA). A gradient from 30% to 60% solution B over 20 min was used, with a peak found at 15.51 min retention time.

HPLC trace for peptide **17** indicating >99% purity:

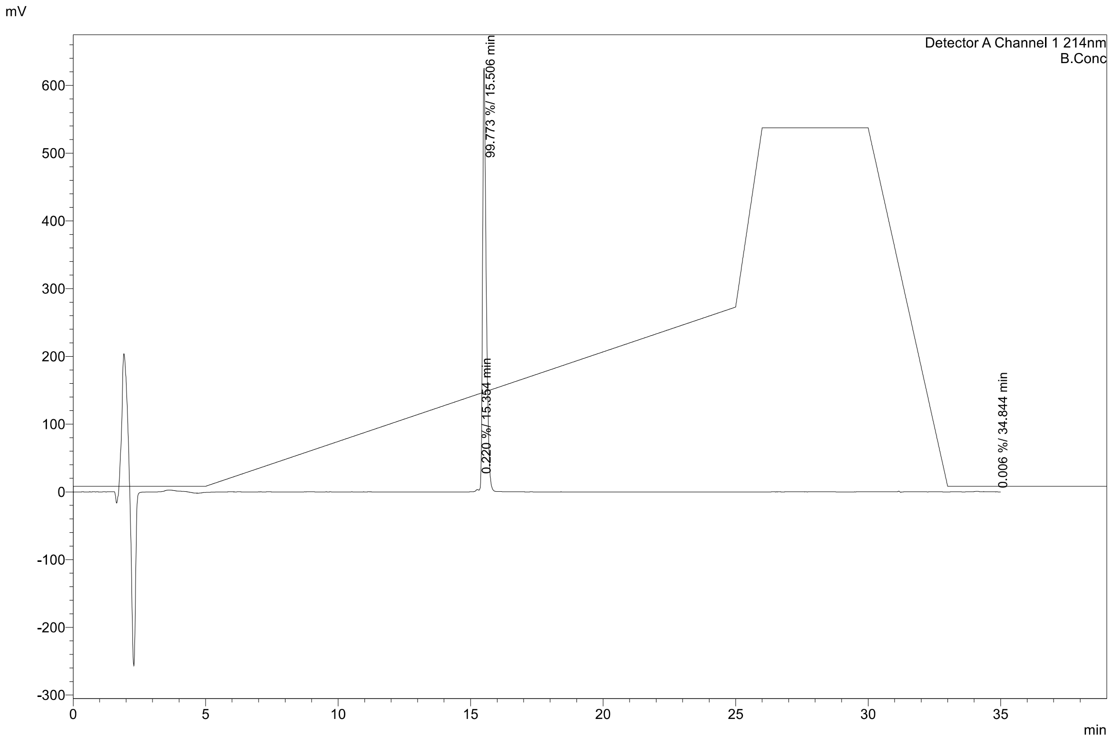

3. Photophysical Data for α-Amino Acids 10a–e and 14.

Spectra were recorded at 1.25–5 μM in methanol.

Absorption and Emission Spectra for 10a (1.25 μM). Excitation at 275 nm.

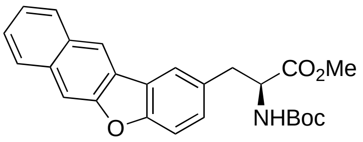

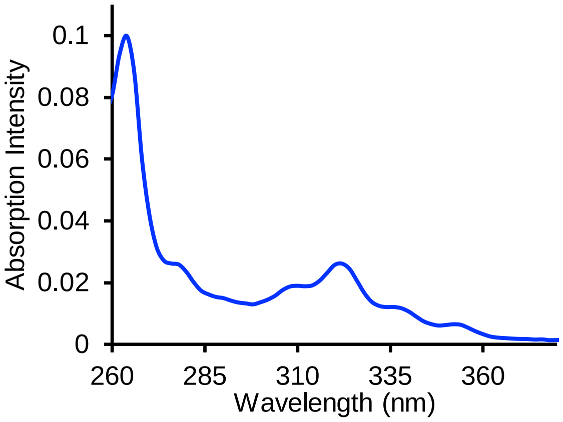

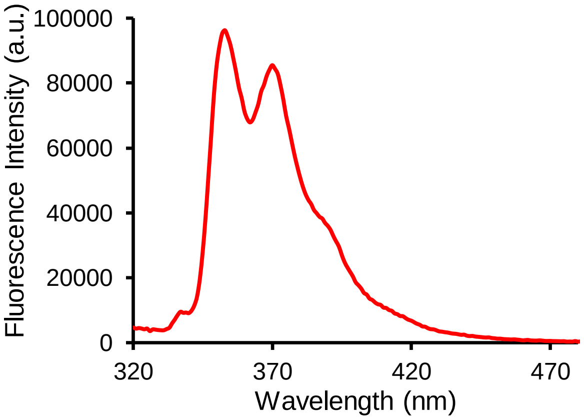

Absorption and Emission Spectra for 10b (5 μM). Excitation at 290 nm.

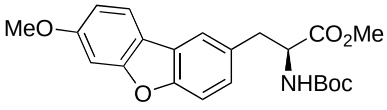

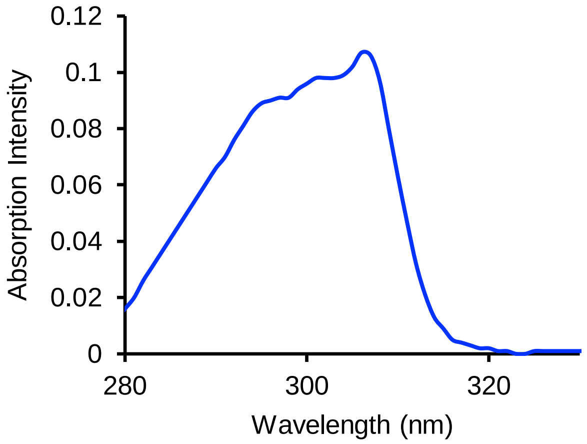

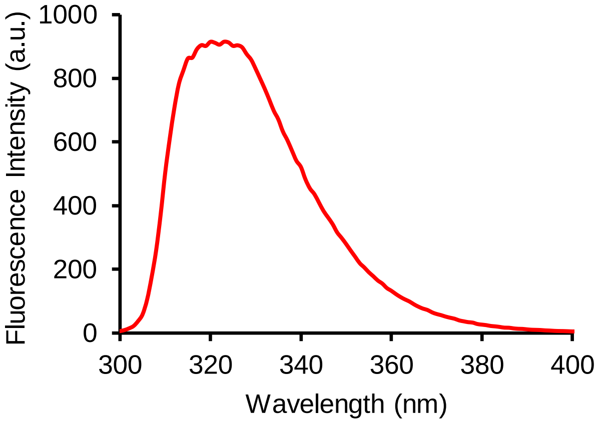

Absorption and Emission Spectra for 10c (4 μM). Excitation at 290 nm.

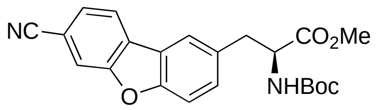

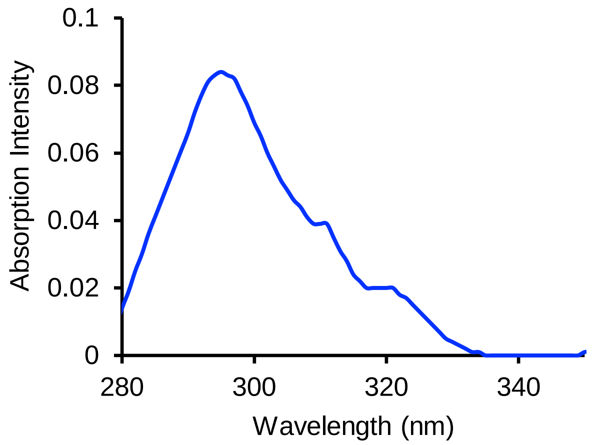

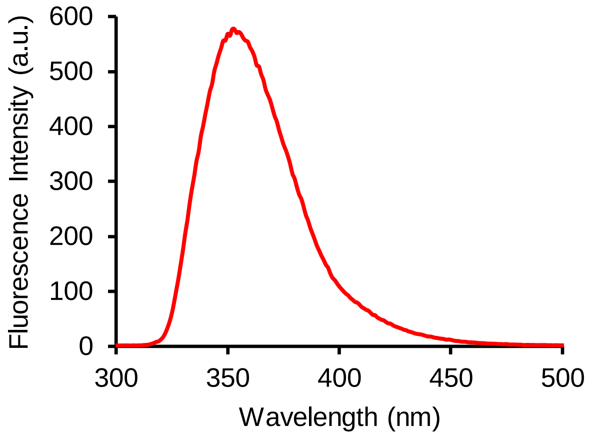

Absorption and Emission Spectra for 10d (5 μM). Excitation at 283 nm.

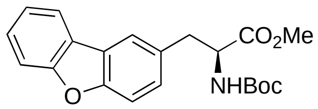

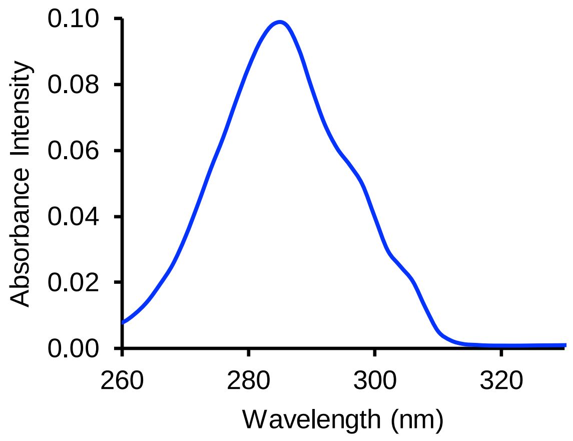

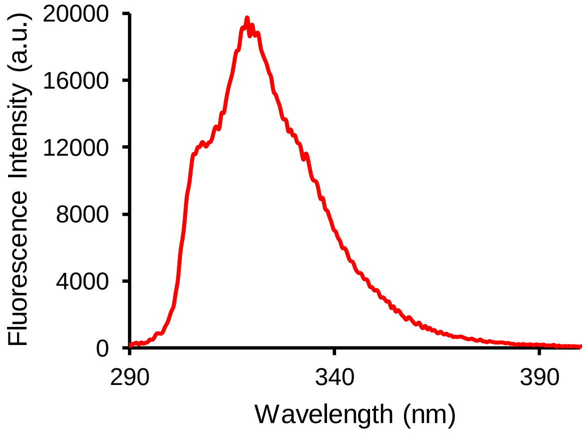

Absorption and Emission Spectra for 10e (5 μM). Excitation at 290 nm.

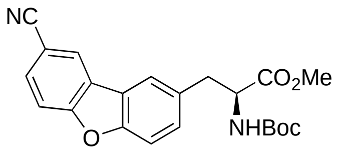

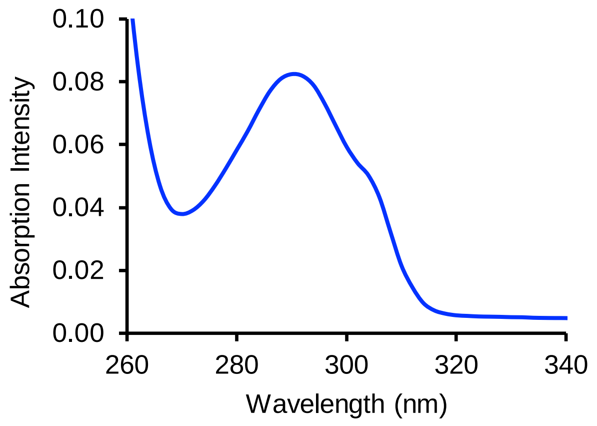

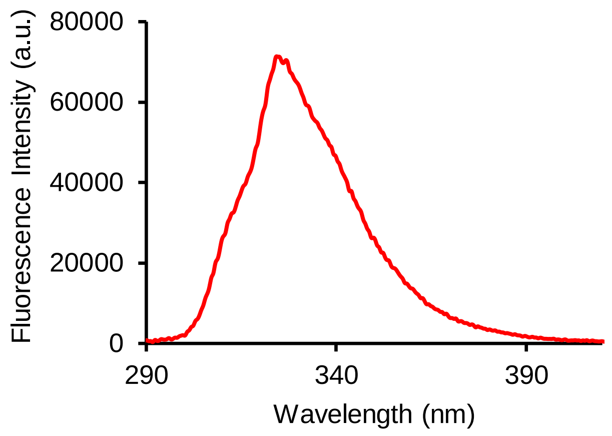

Absorption and Emission Spectra for 14 (5 μM). Excitation at 290 nm.

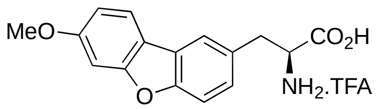

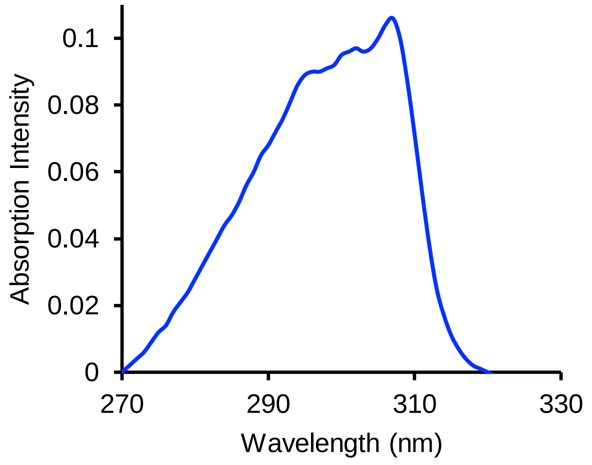

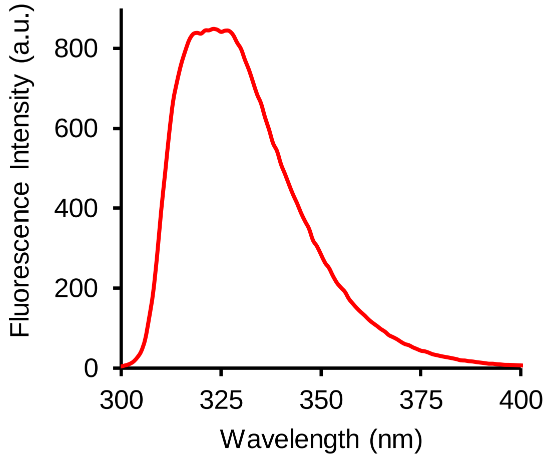

Additional Photophysical Data for 14.

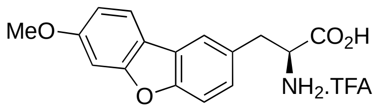

Solvatochromic Study:

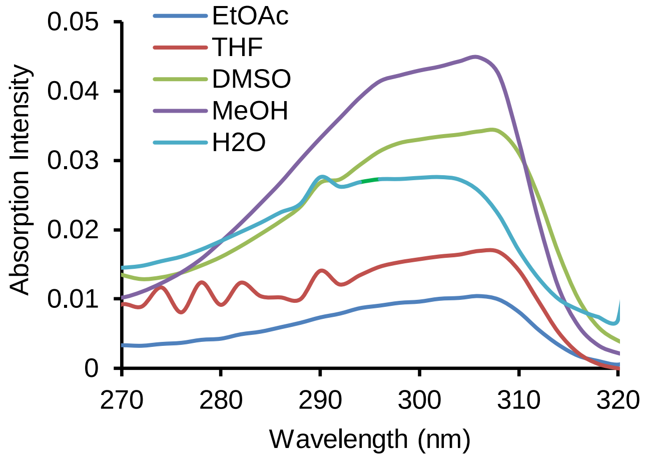

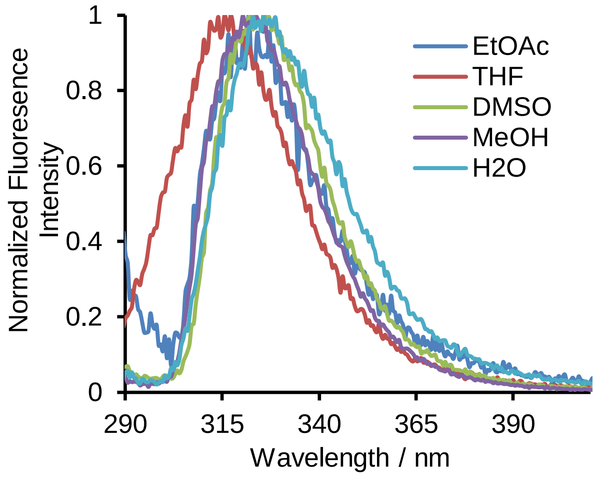

Lippert-Mataga Plot:

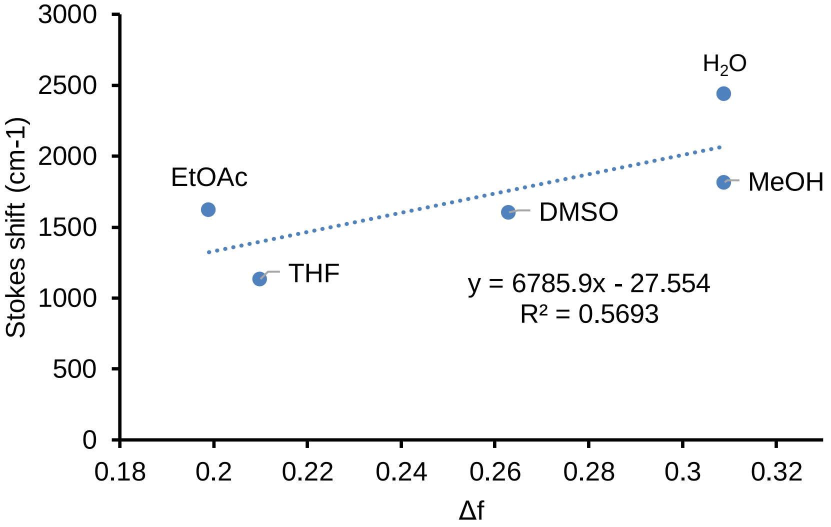

4. Determination of Förster Distance for Amino acid 14/Lysine(dnp) Pair and Reaction of Decapeptide 17 with Trypsin

Determination of Förster distance (R_0) for amino acid 14/lysine(dnp) pair

Förster distance, R_0, was determined using the following equation[3]:

$$R_0 = 0.211[\kappa^2\, \eta^{-4} Q_D J(\lambda)]^{1/6}$$

where, κ^2 is a factor describing the relative orientation in space between two transition dipoles (typically assumed to be equal to 2/3); η is the refractive index of the medium and Q_D is the quantum yield of the donor in the absence of the acceptor. J(λ) is the overlap integral between the donor emission and acceptor absorption and can be calculated using the following equation:

$$J(\lambda) = \frac{\int_0^\infty F_D(\lambda)\varepsilon_A(\lambda)\lambda^4 d\lambda}{\int_0^\infty F_D(\lambda)d\lambda}$$

where λ is the wavelength and ε_A is the extinction coefficient of the acceptor. J(λ) can also be expressed by the following equation for calculating the spectral overlap in excel[4]:

$$J(\lambda) = \frac{\sum_{\lambda=300,\lambda\in N}^{600} F_D(\lambda)\varepsilon_A\lambda^4}{\sum_{\lambda=300,\lambda\in N}^{600} F_D(\lambda)}$$

Emission and absorption spectra of amino acid **14** and lysine(dnp) were recorded in methanol at a concentration of 5 μM. Förster distance calculation was performed in excel using template provided by Hink and Visser.[5] This gave a Förster distance between the FRET pair of 34.20 Å.

Spectroscopic overlap between emission of amino acid **14** (red) and absorption of lysine(dnp) (blue):

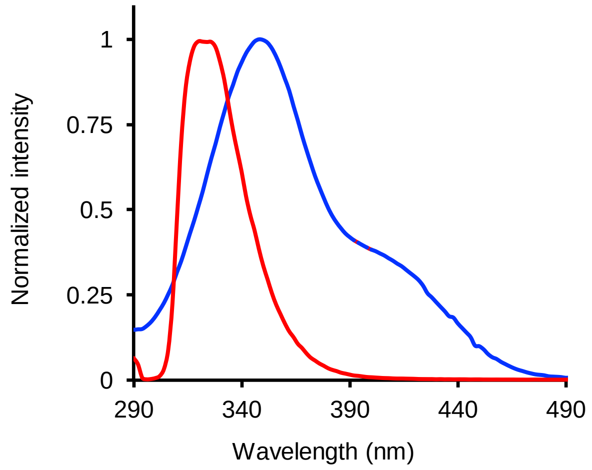

Trypsin Digestion of Decapeptide 17

Spectra were obtained using a Horiba Duetta Fluorescence and Absorbance spectrometer at 25 °C. A 11 µM solution of TPCK-treated trypsin (Sigma, 1000–2000 units/mg, 0.25 mg/mL) was prepared in 1 mM aqueous hydrochloric acid and stored frozen. A portion of a stock solution of decapeptide **17** was diluted to 10 µM in 3-morpholinopropanesulfonic acid (MOPS) buffer (20 mM, pH 7.0) to a total volume of 1 mL. The trypsin stock solution (2 µL, 0.02 µM) was added and the emission spectra were measured by excitation at 290 nm and scanning from 290 nm to 600 nm. The emission spectra of the sample were collected regularly until constant fluorescence intensity (200 minutes).

Increase in Emission over Time:

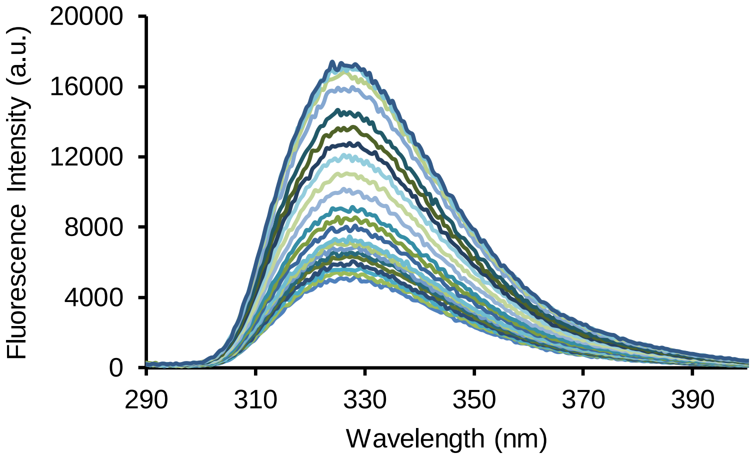

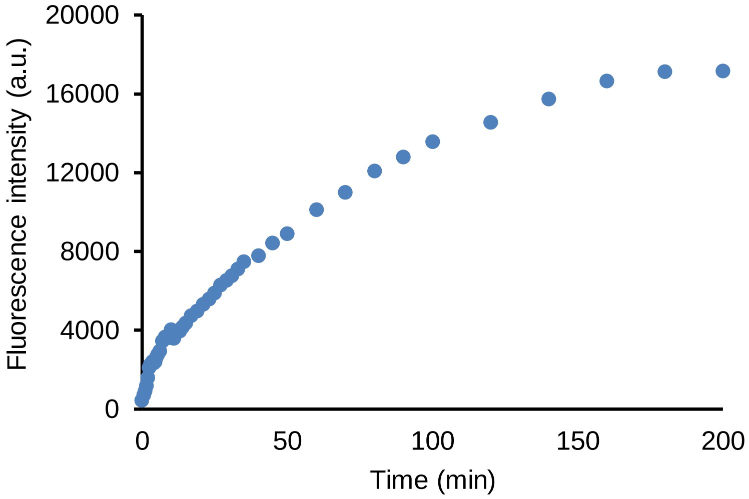

Emission of decapeptide 17 before addition of trypsin (3% relative intensity versus cleaved peptide):

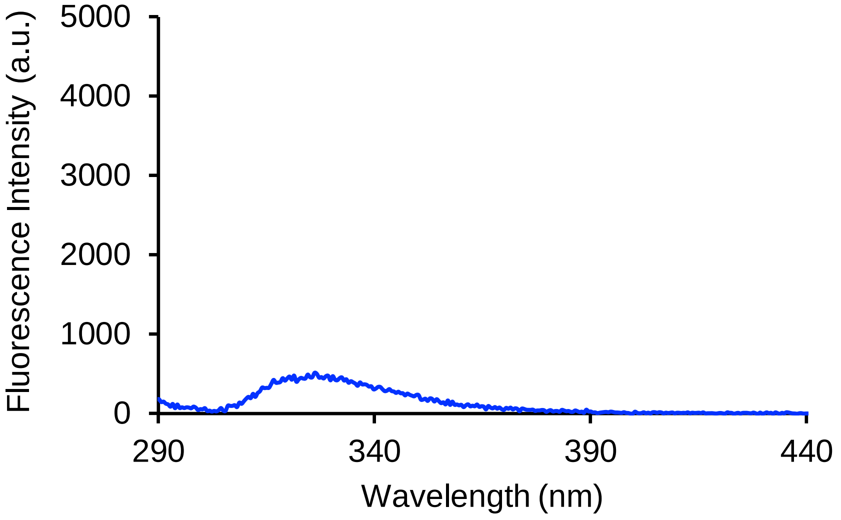

Trypsin cleaved peptide after 200 minutes:

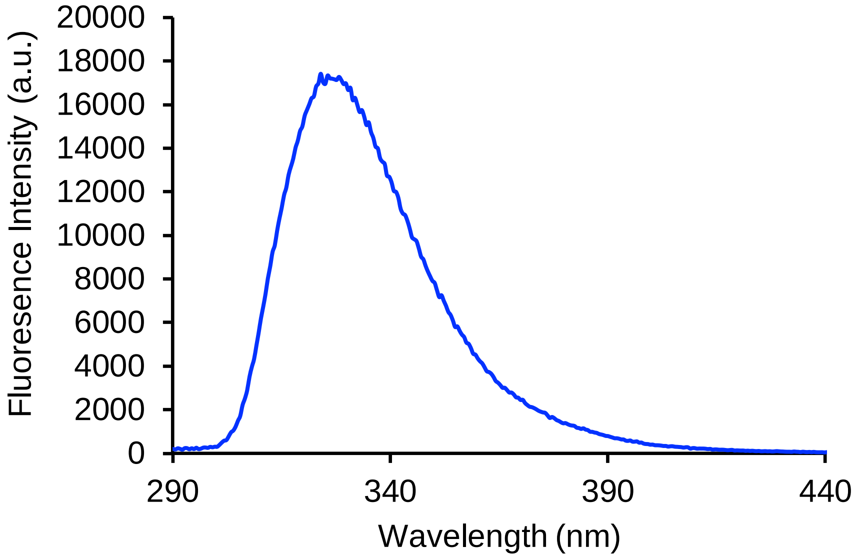

5. References

1. Georgiev, D.; Saes, B. W. H.; Johnston, H. J.; Boys, S. K.; Healy, A.; Hulme, A. N. Selective and Efficient Generation of *ortho*-Brominated *para*-Substituted Phenols in ACS-Grade Methanol. *Molecules* **2016**, *21*, https://doi.org/10.3390/molecules21010088.

2. Stempel, E.; Kaml, R. F.-X.; Budisa, N.; Kalesse, M. Painting Argyrins Blue: Negishi Cross-Coupling for Synthesis of Deep-Blue Tryptophan Analogue β-(1-Azulenyl)-L-Alanine and its Incorporation into Argyrin C. *Bioorg. Med. Chem*. **2018**, *26*, 5259–5269.

3. J. R. Lakowicz, Principles of Fluorescence Spectroscopy, Springer, New York, 2006.

4. M. Poreba, A. Szalek, W. Rut, P. Kasperkiewicz, I. Rutkowska-Wlodarczyk, S. J. Snipas, Y. Itoh, D. Turk, B. Turk, C. M. Overall, L. Kaczmarek, G. S. Salvesen and M. Drag, *Sci. Rep*., 2017, **7**, 43135.

5. Critical Transfer Distance Determination Between FRET Pairs, http://photobiology.info/Experiments/Biolum-Expt.html (accessed September 2024).

6. ^{1}H and ^{13}C NMR Spectra for all Novel Compounds

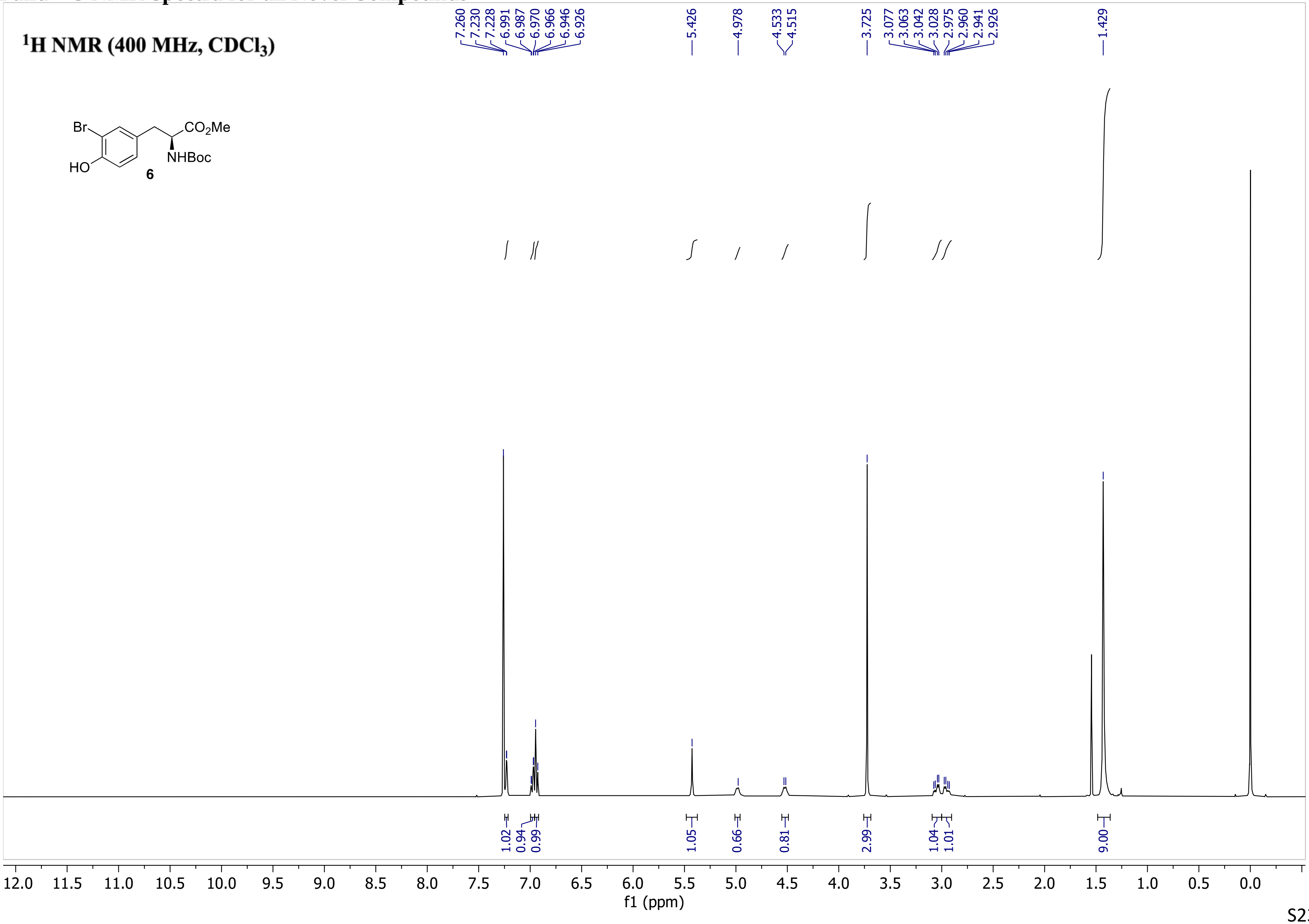

$^{13}C\{^{1}H\}$ NMR (101 MHz, $CDCl_3$)

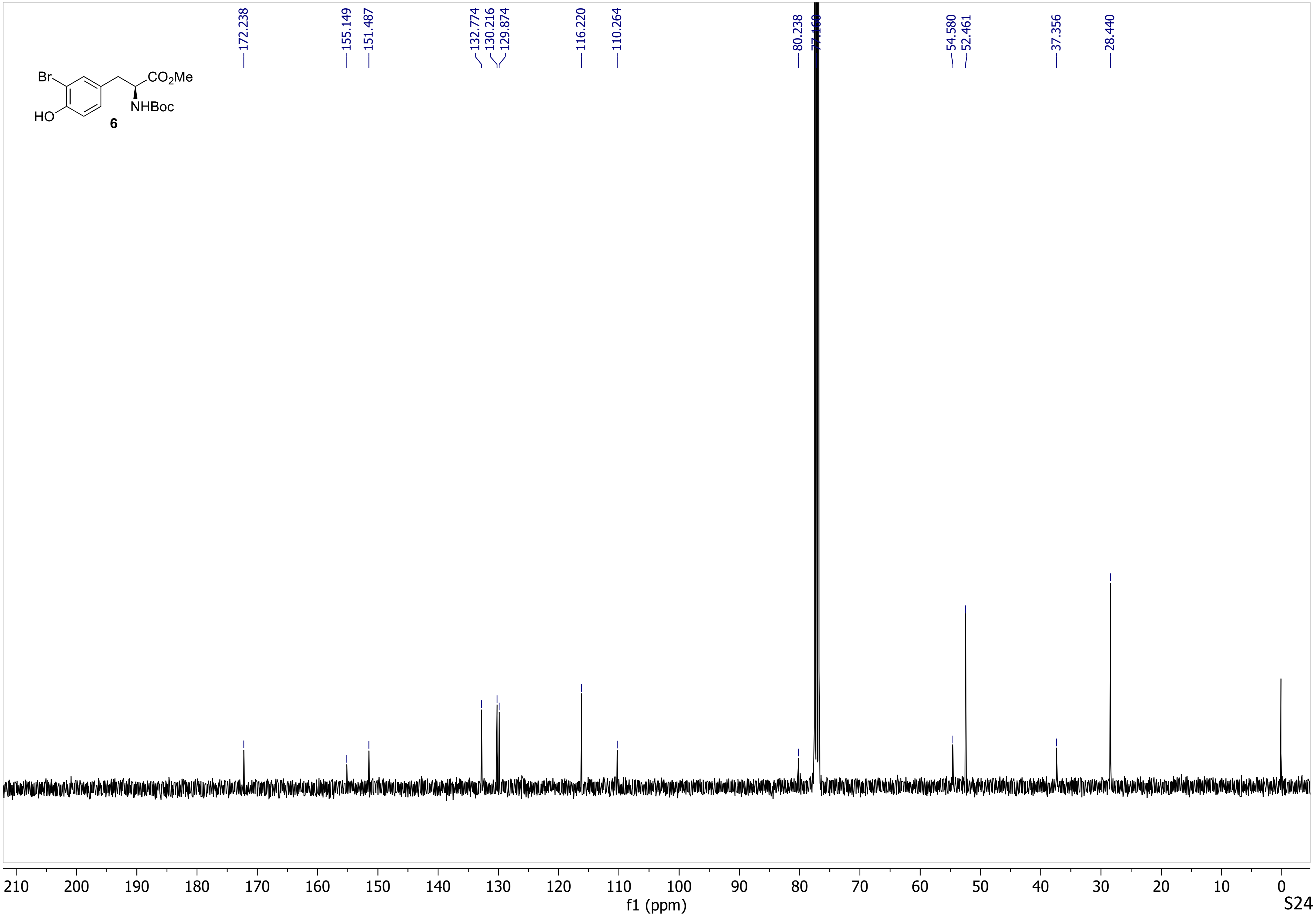

^{1}H NMR (400 MHz, $CDCl_3$)

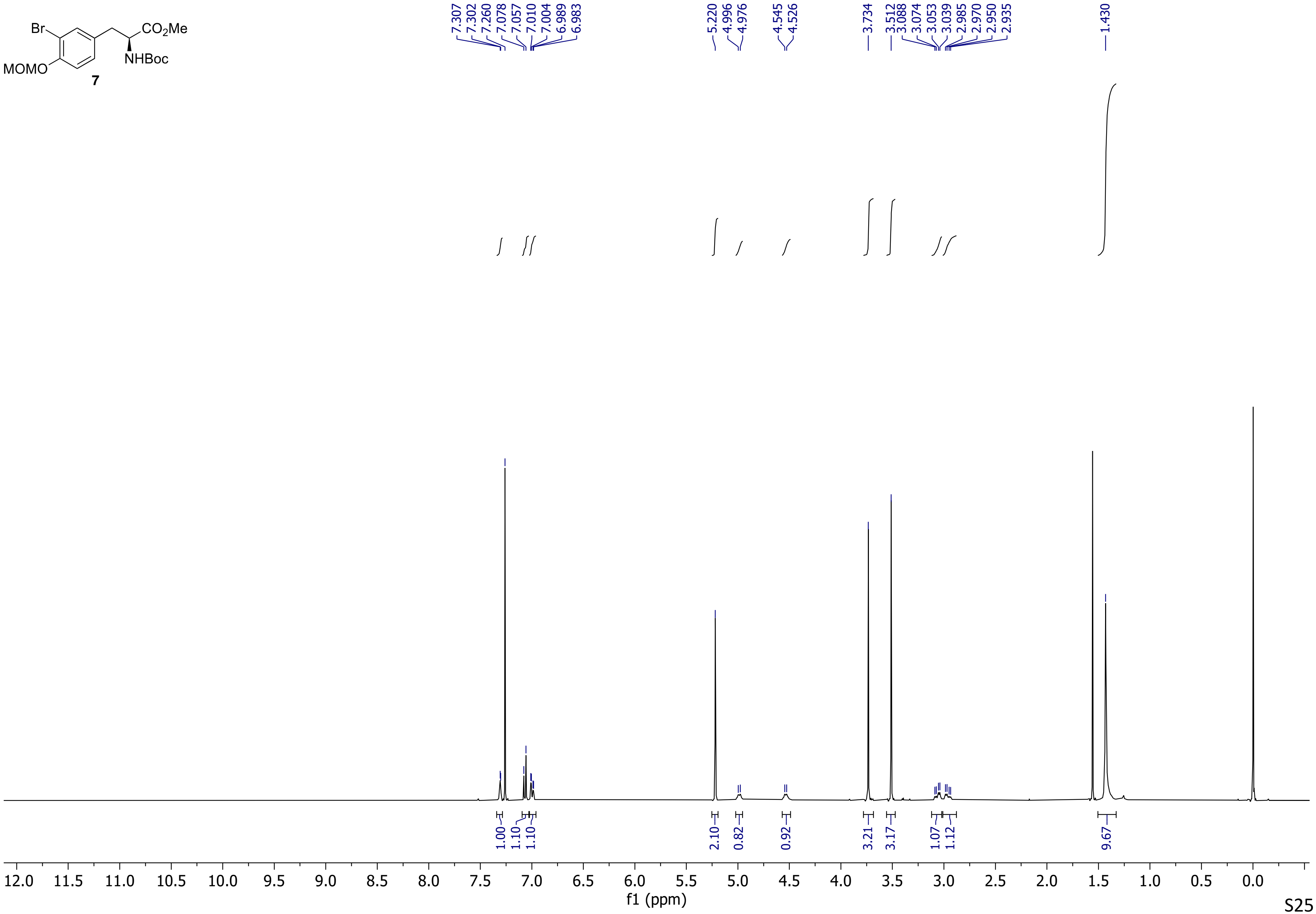

$^{13}C\{^1H\}$ NMR (101 MHz, $CDCl_3$)

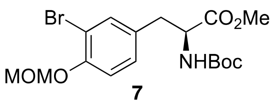

7

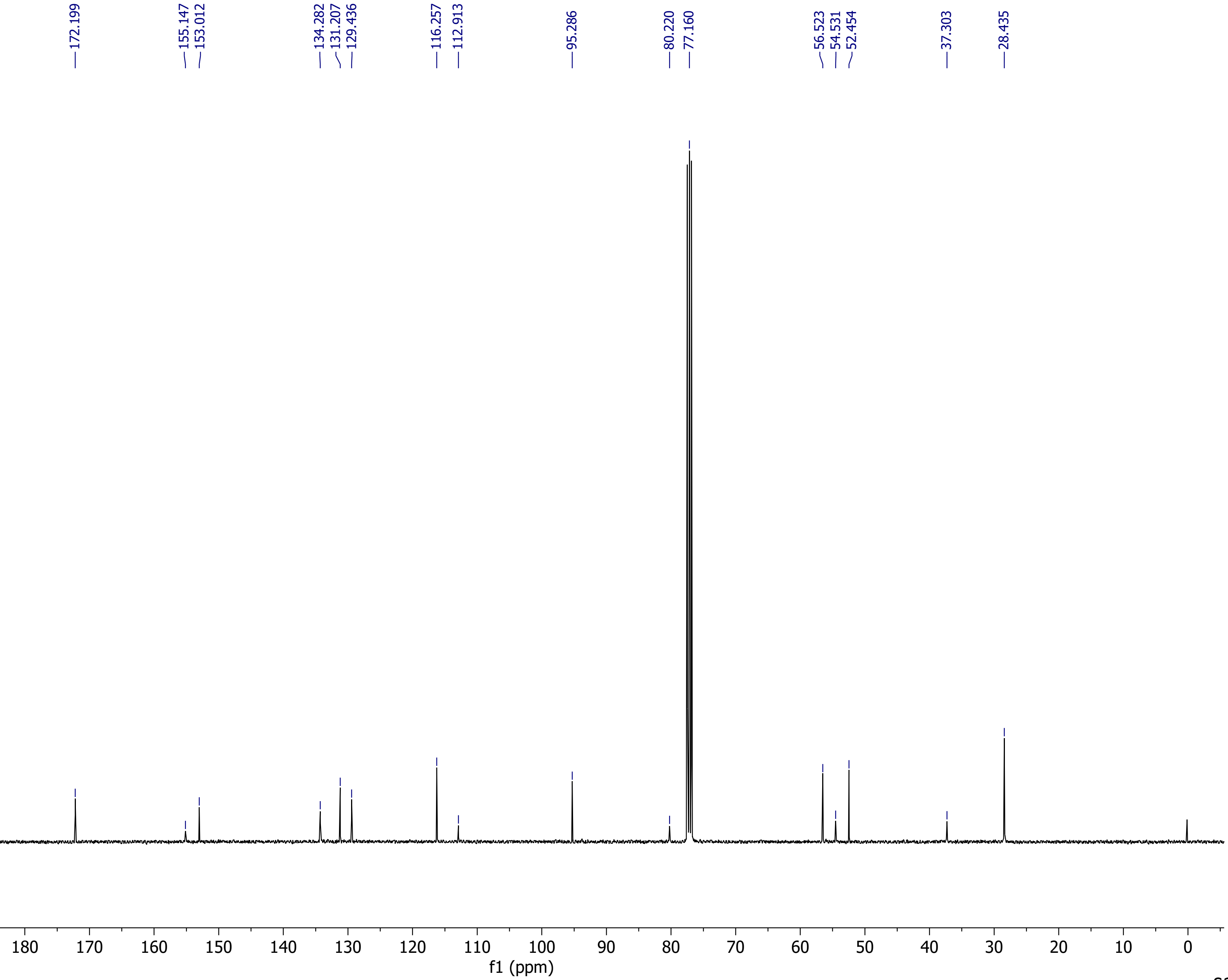

^1H NMR (400 MHz, $CDCl_3$)

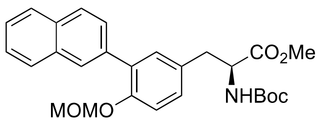

8a

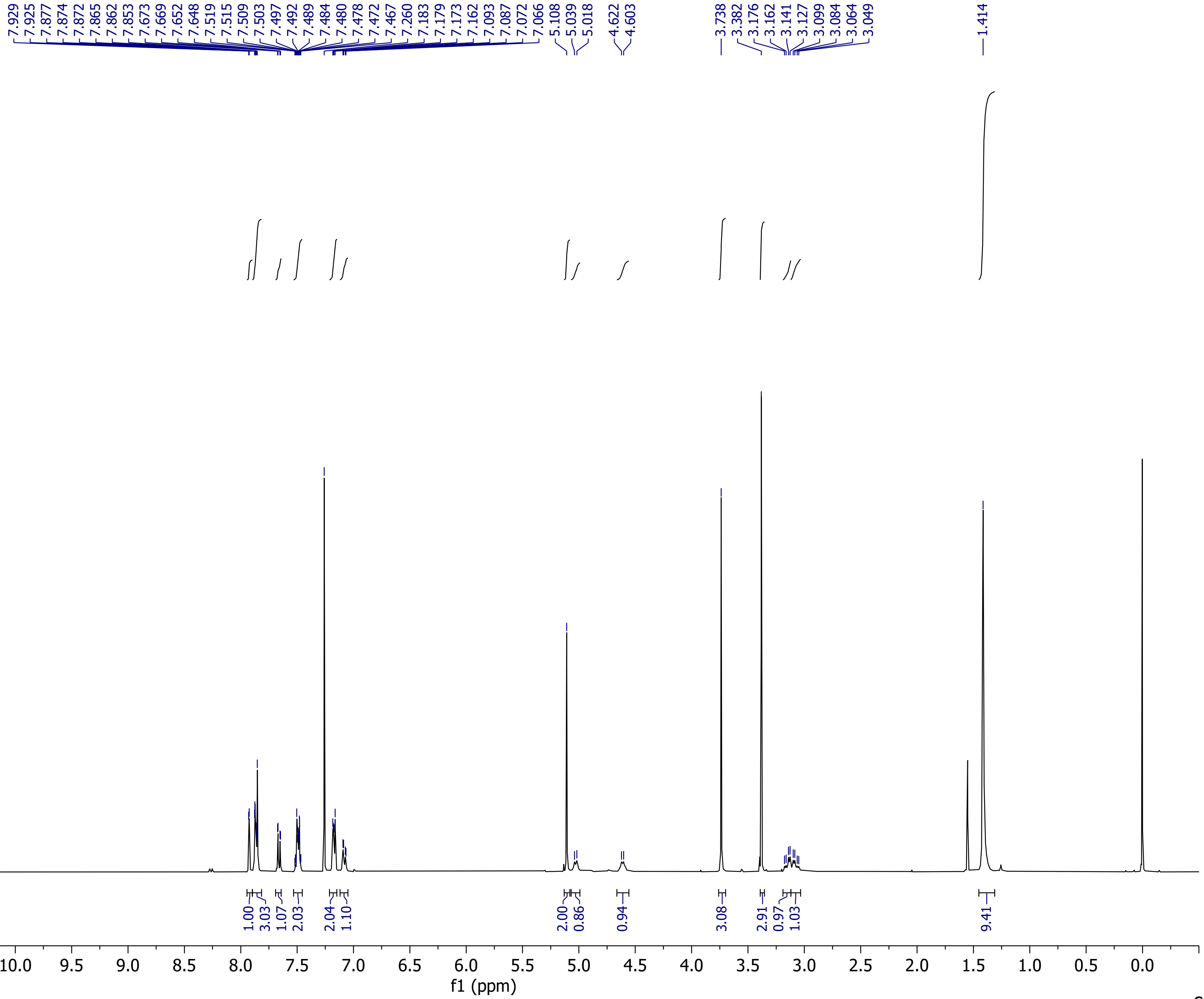

$^{13}C\{^{1}H\}$ NMR (101 MHz, $CDCl_3$)

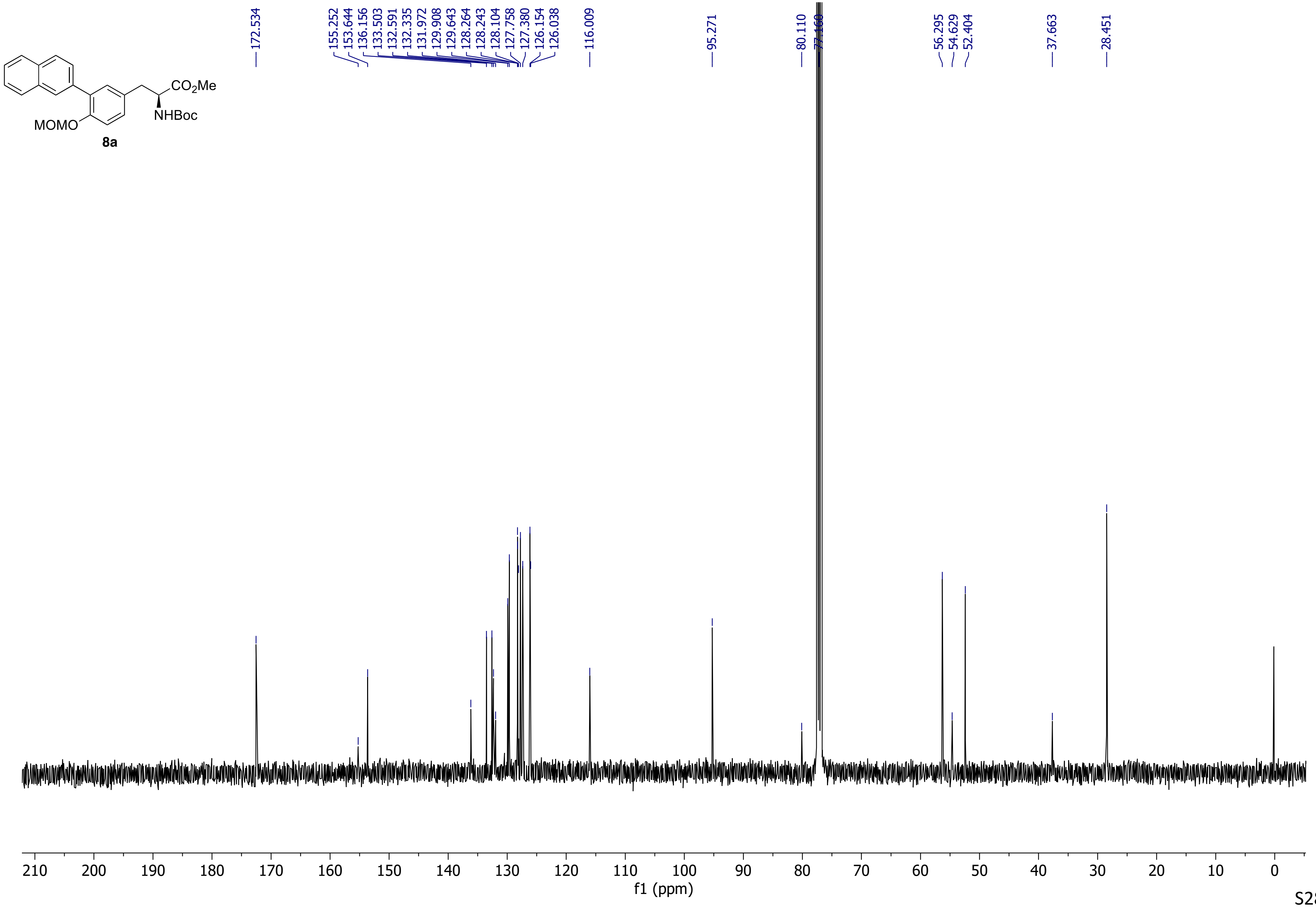

^{1}H NMR (400 MHz, $CDCl_3$)

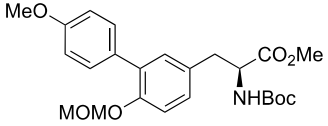

8b

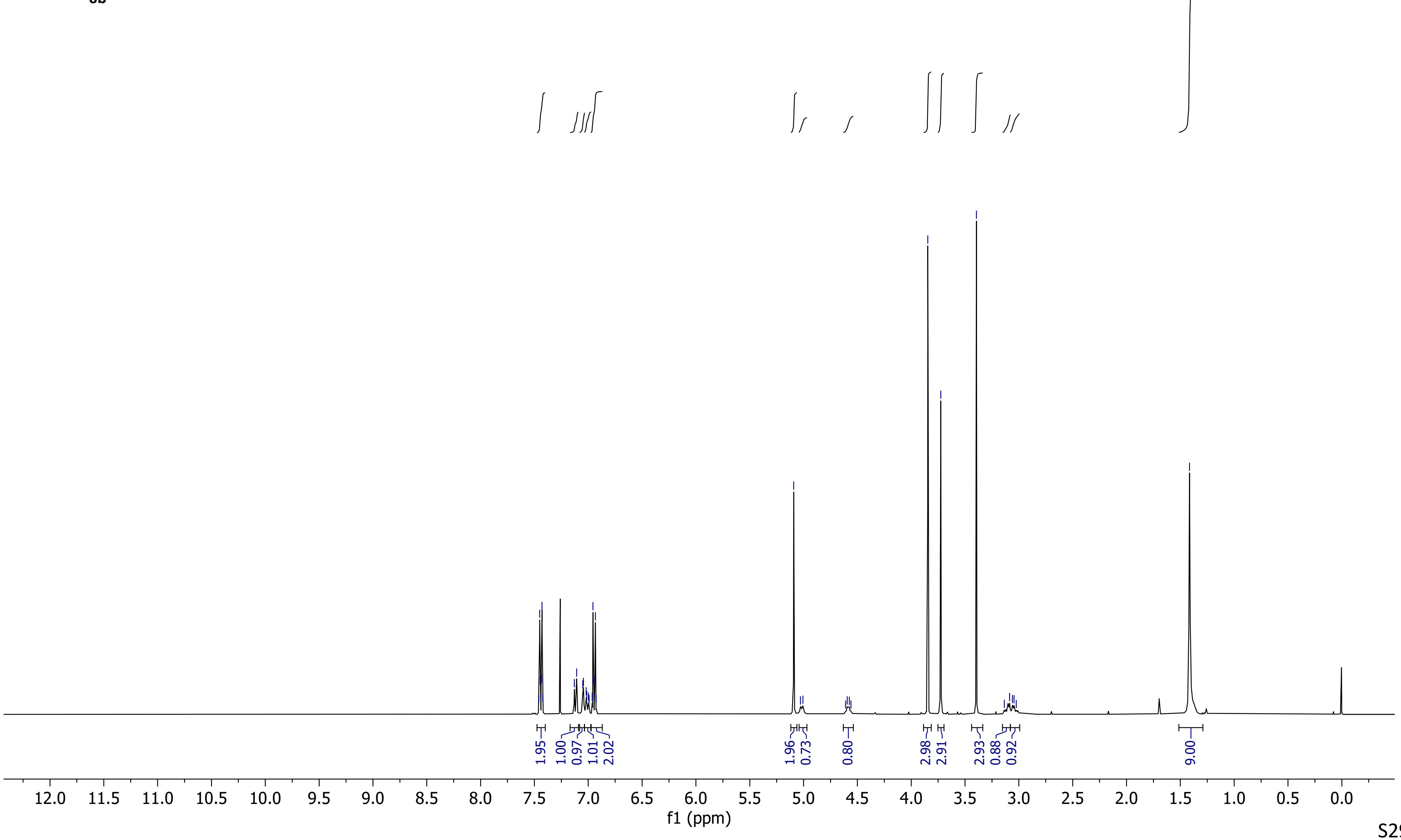

$^{13}C\{^{1}H\}$ NMR (101 MHz, $CDCl_3$)

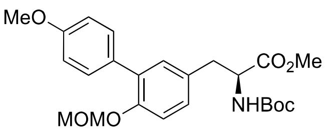

8b

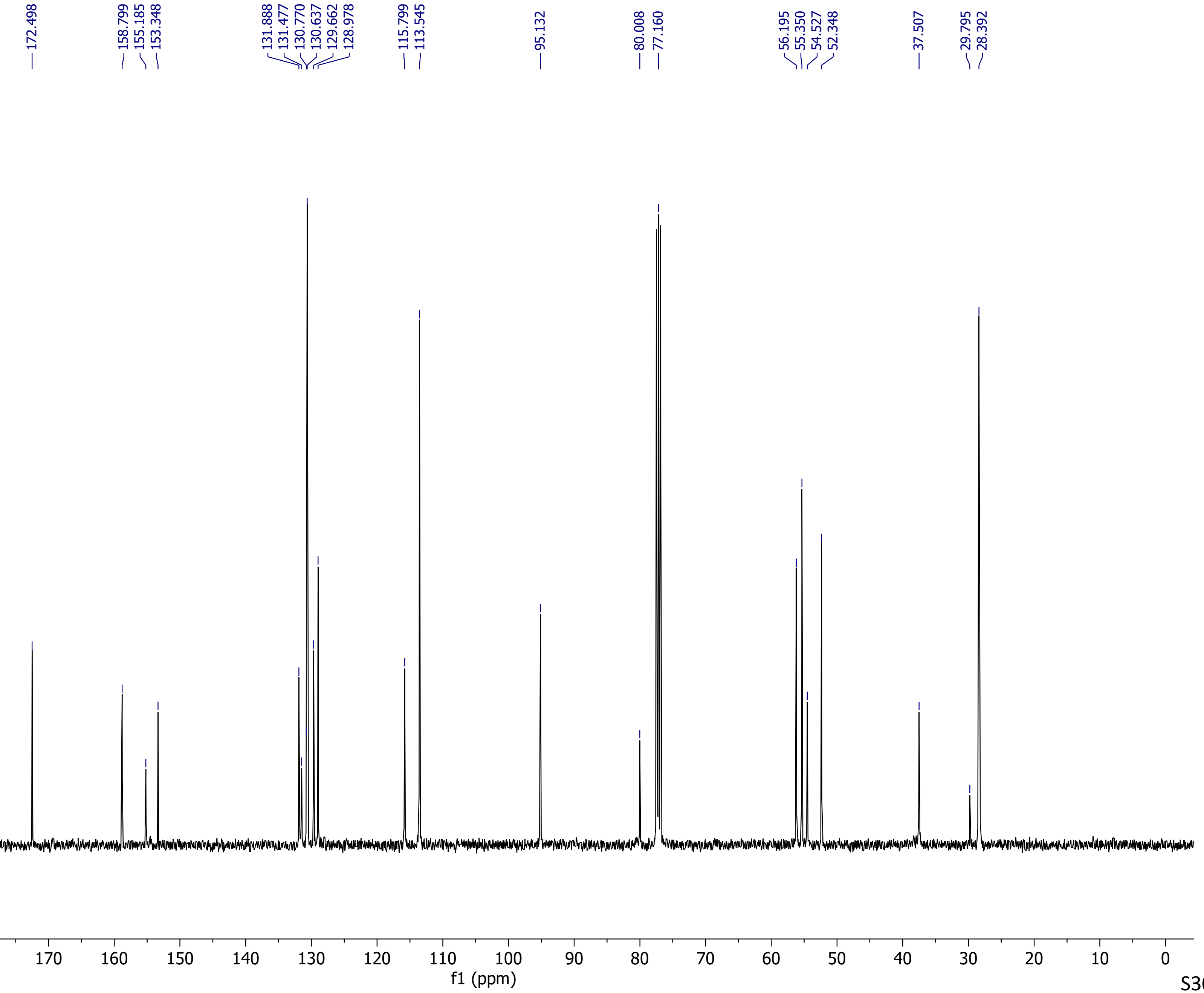

^{1}H NMR (400 MHz, $CDCl_3$)

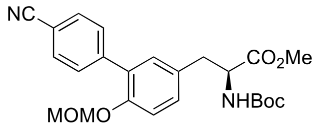

8c

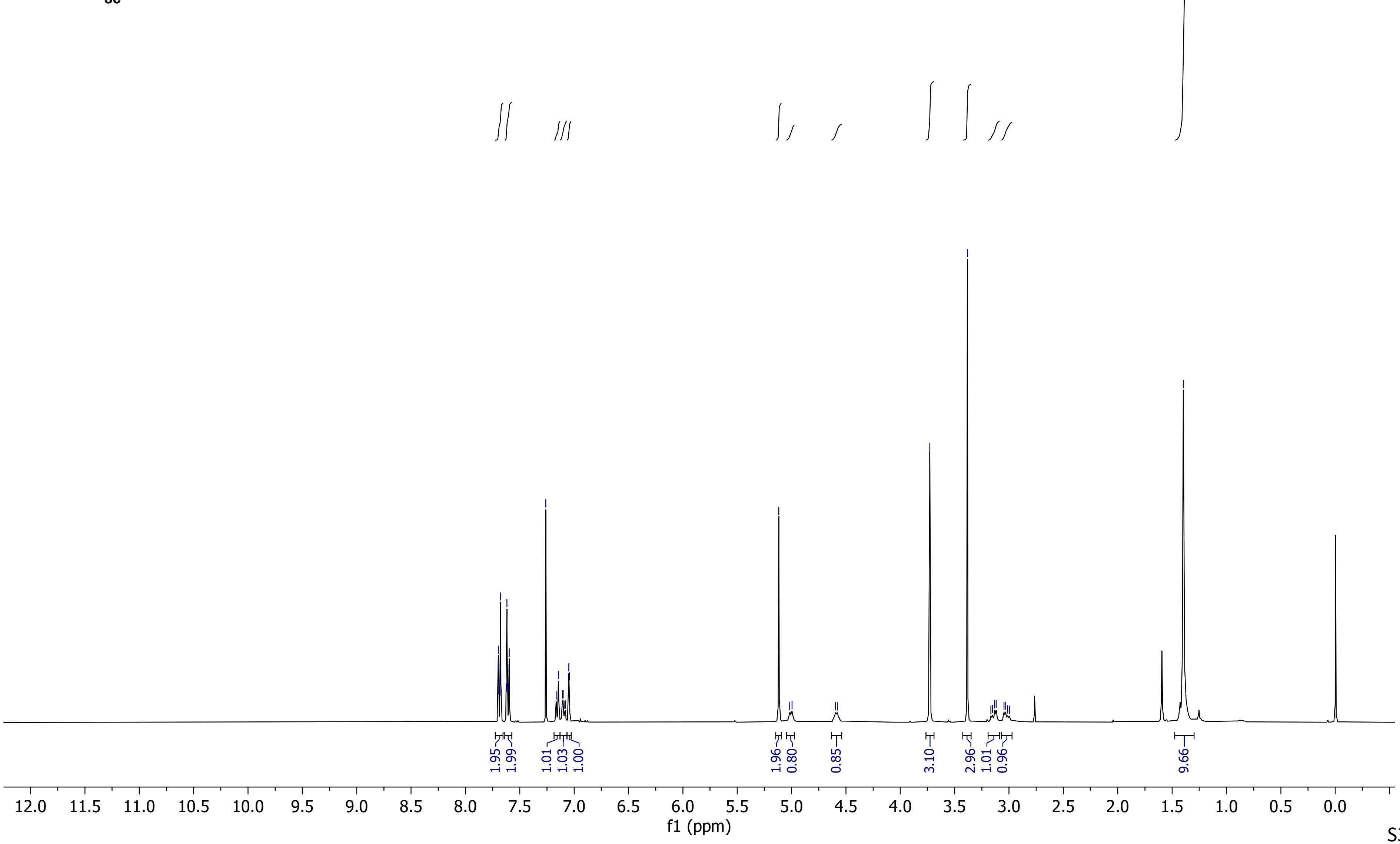

$^{13}C\{^1H\}$ NMR (101 MHz, $CDCl_3$)

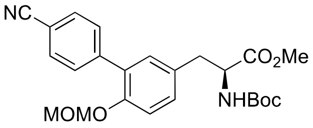

8c

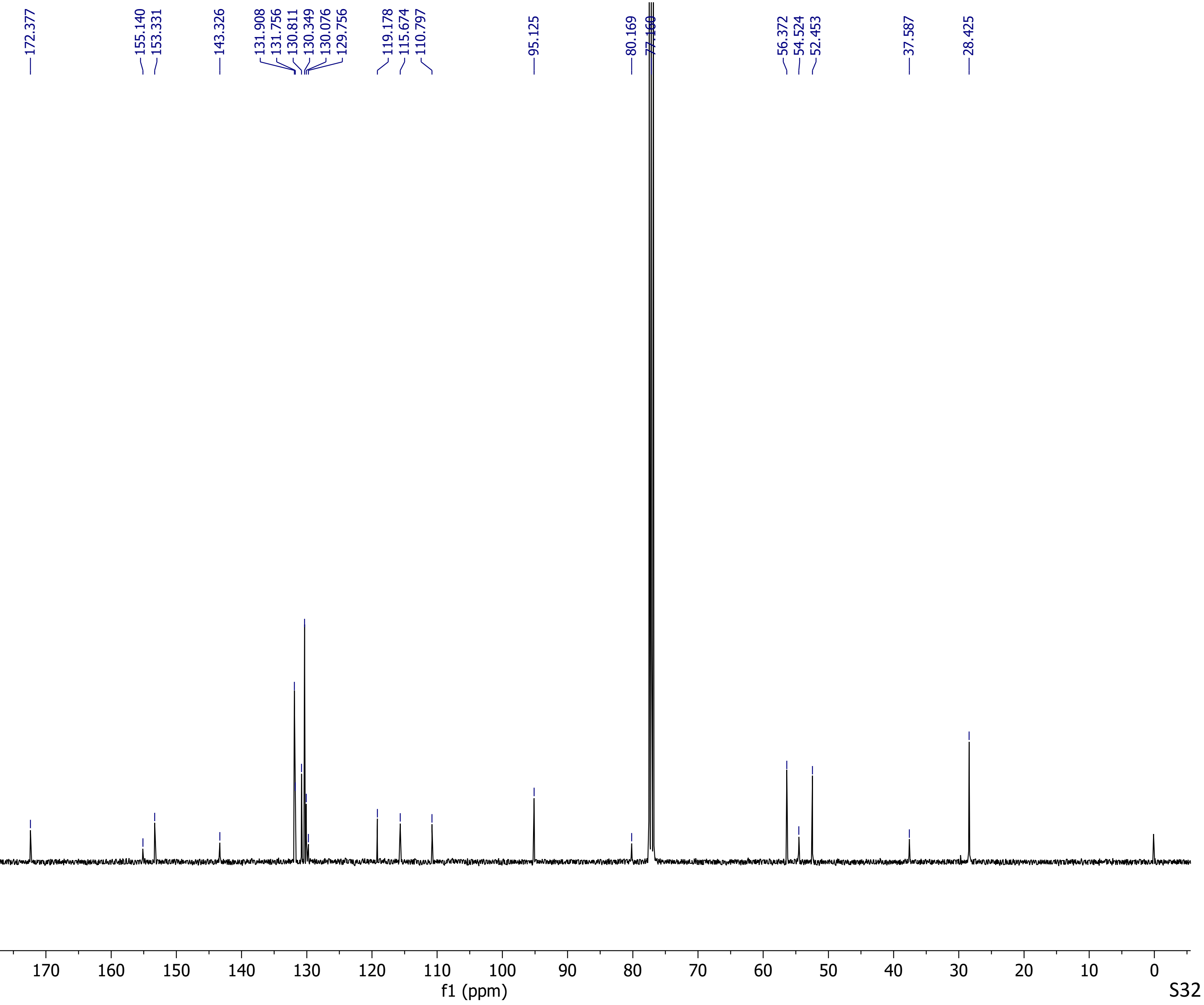

^{1}H NMR (400 MHz, $CDCl_3$)

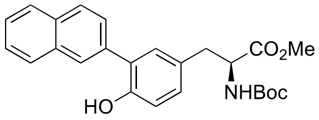

9a

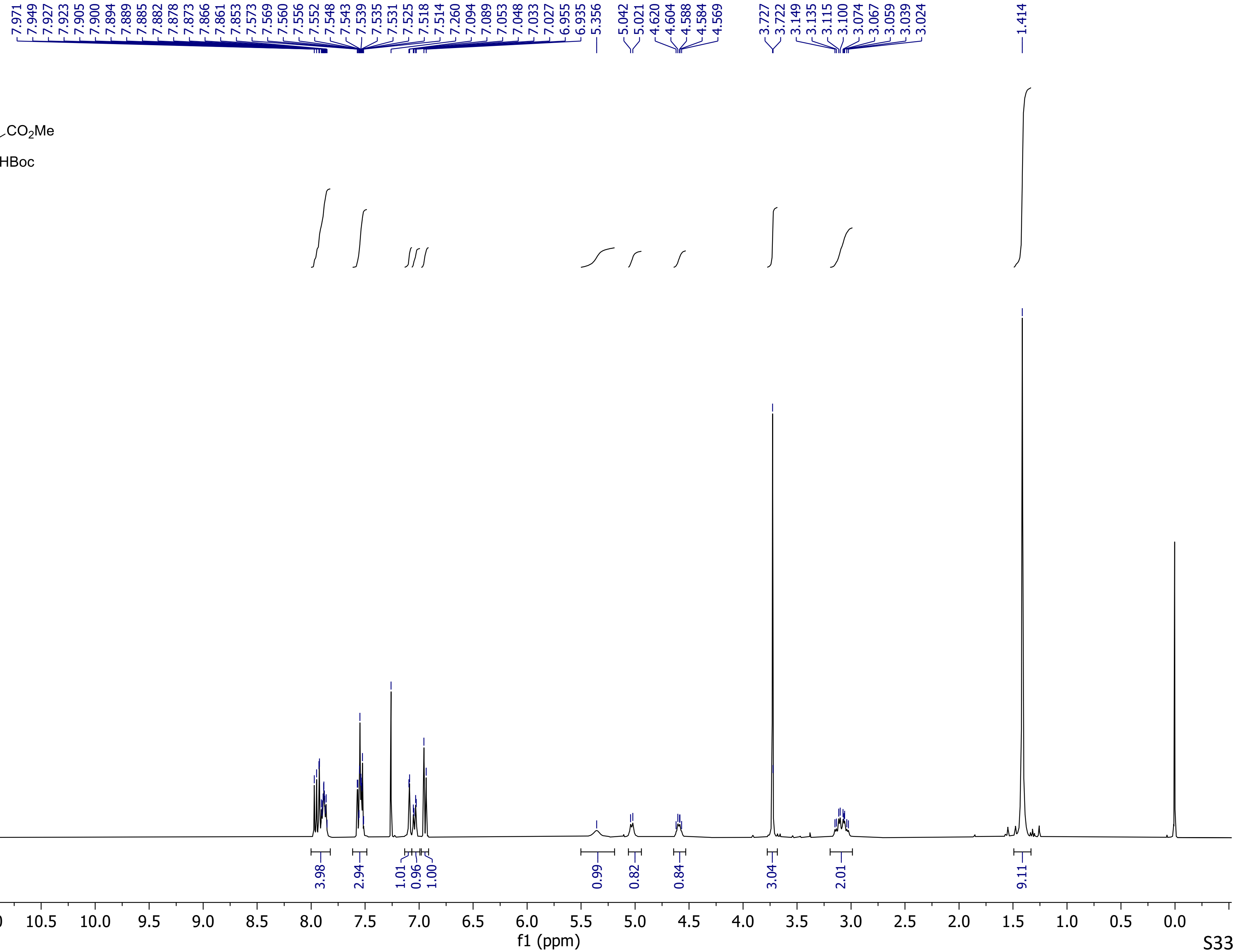

$^{13}C\{^{1}H\}$ NMR (101 MHz, $CDCl_3$)

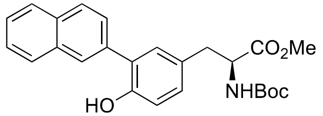

9a

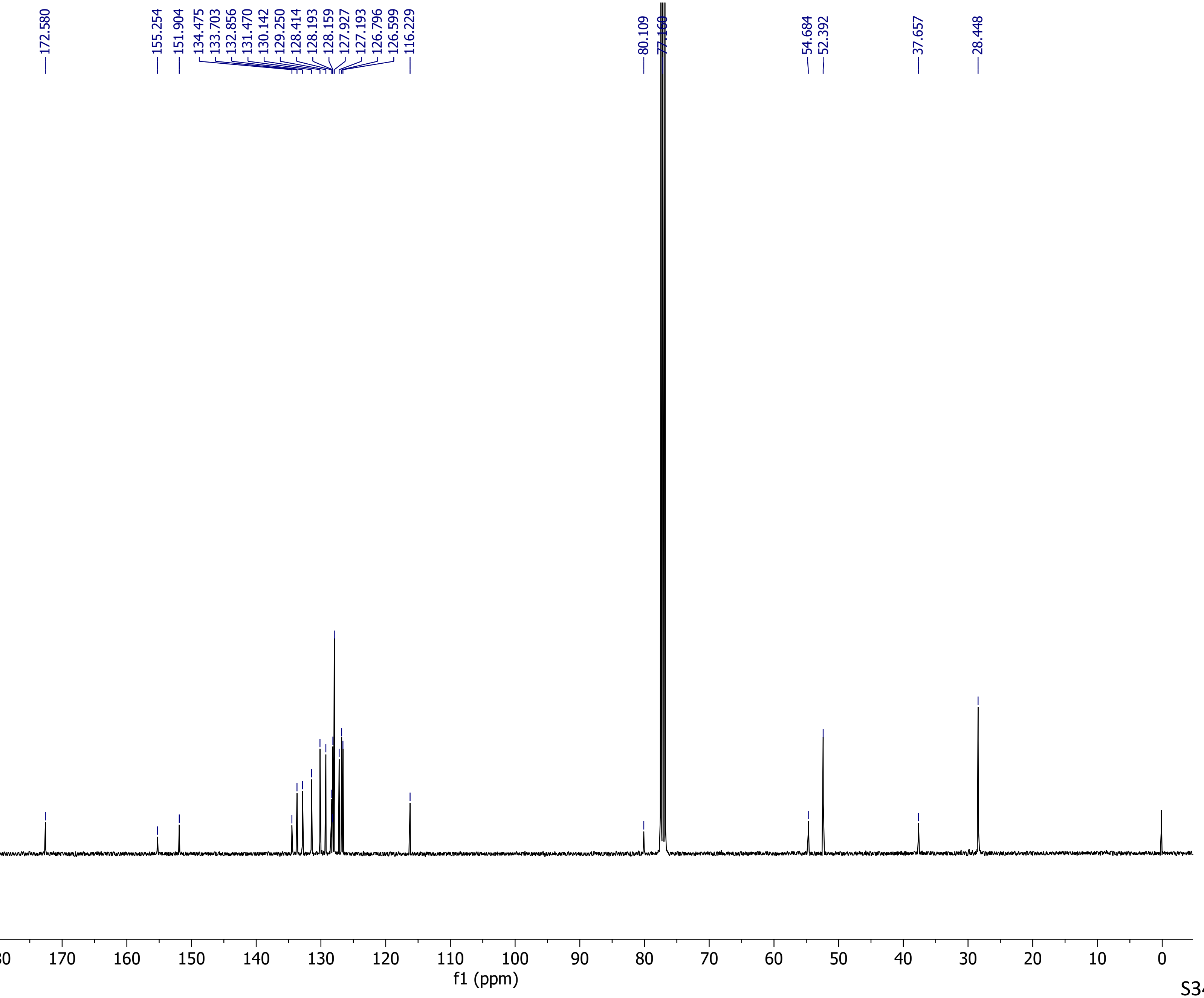

^{1}H NMR (400 MHz, $CDCl_3$)

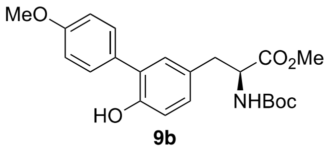

9b

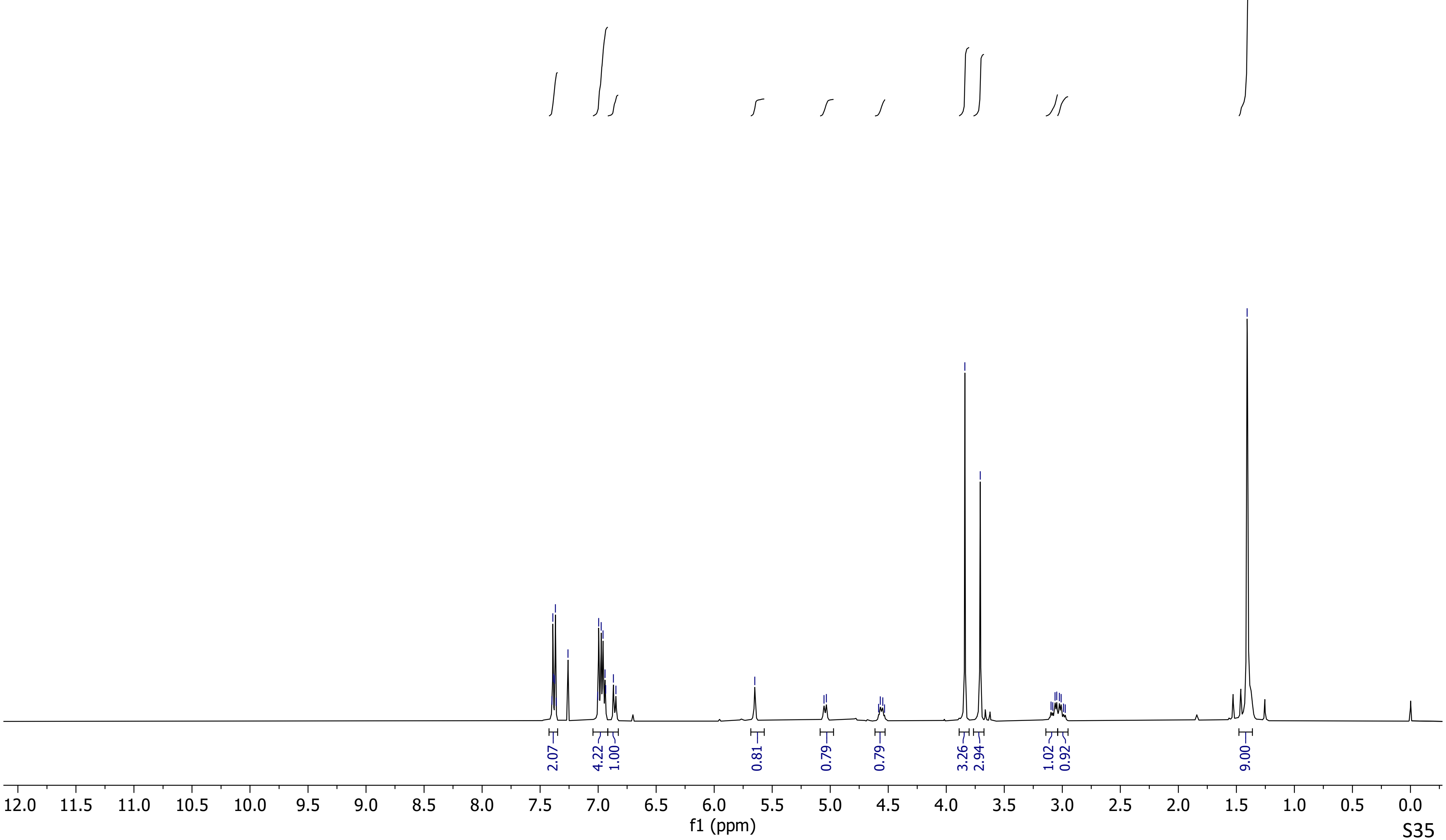

$^{13}C\{^{1}H\}$ NMR (101 MHz, $CDCl_3$)

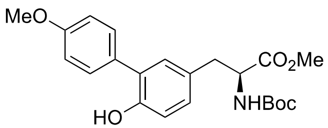

9b

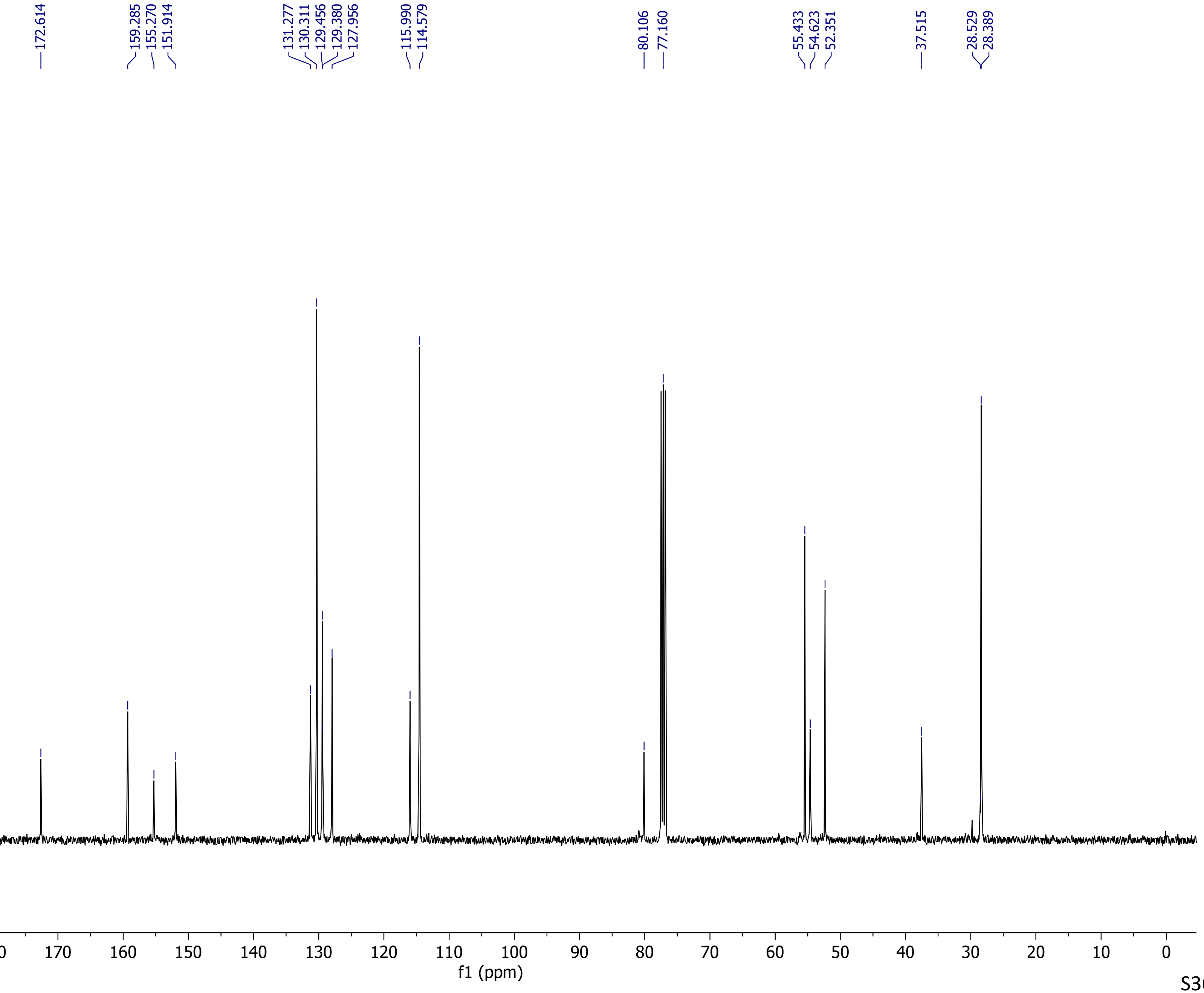

^{1}H NMR (400 MHz, $CDCl_3$)

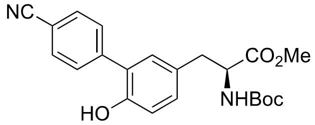

9c

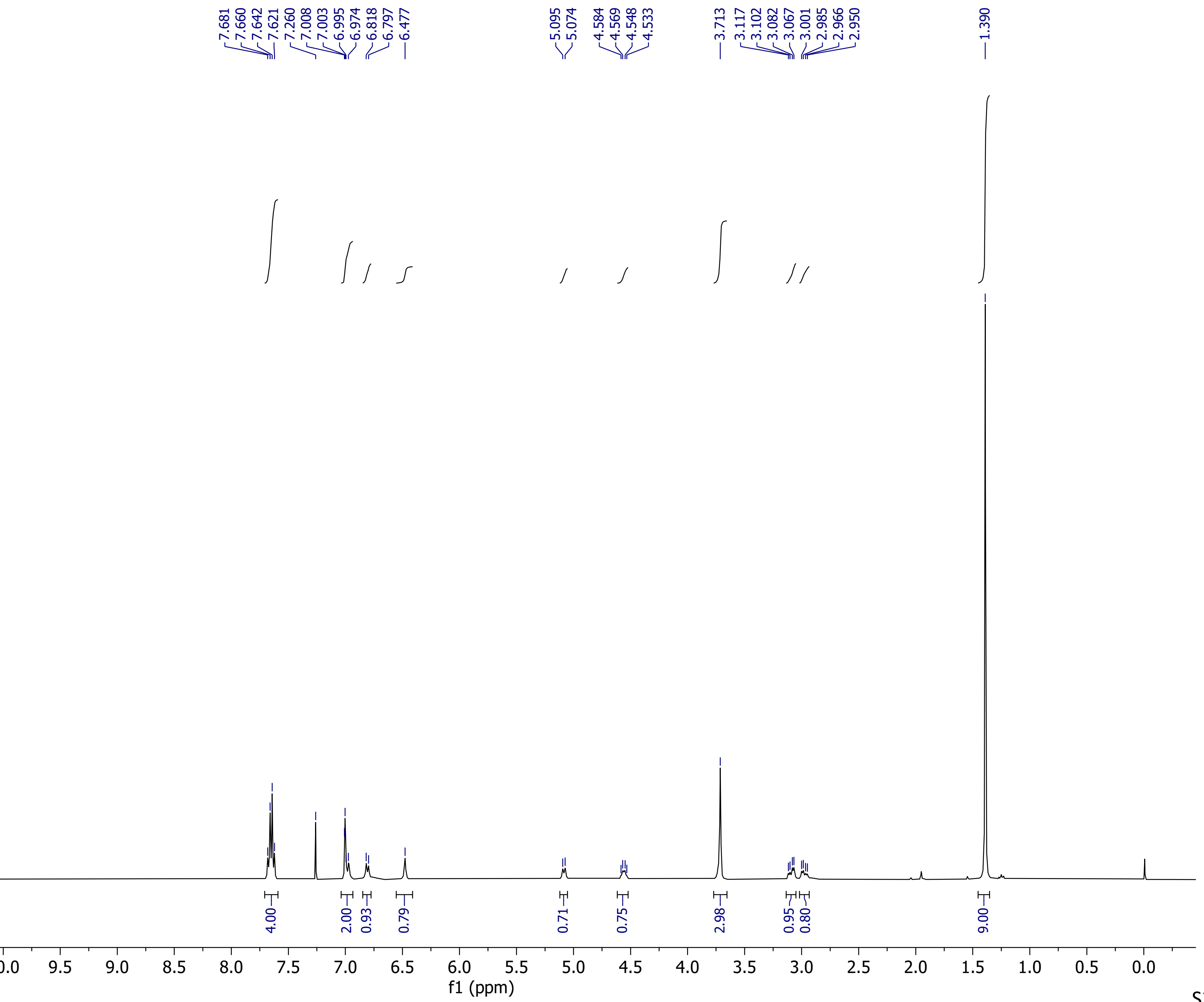

$^{13}C\{^1H\}$ NMR (101 MHz, CDCl$_3$)

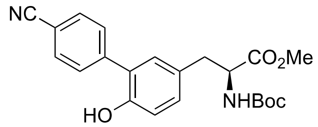

9c

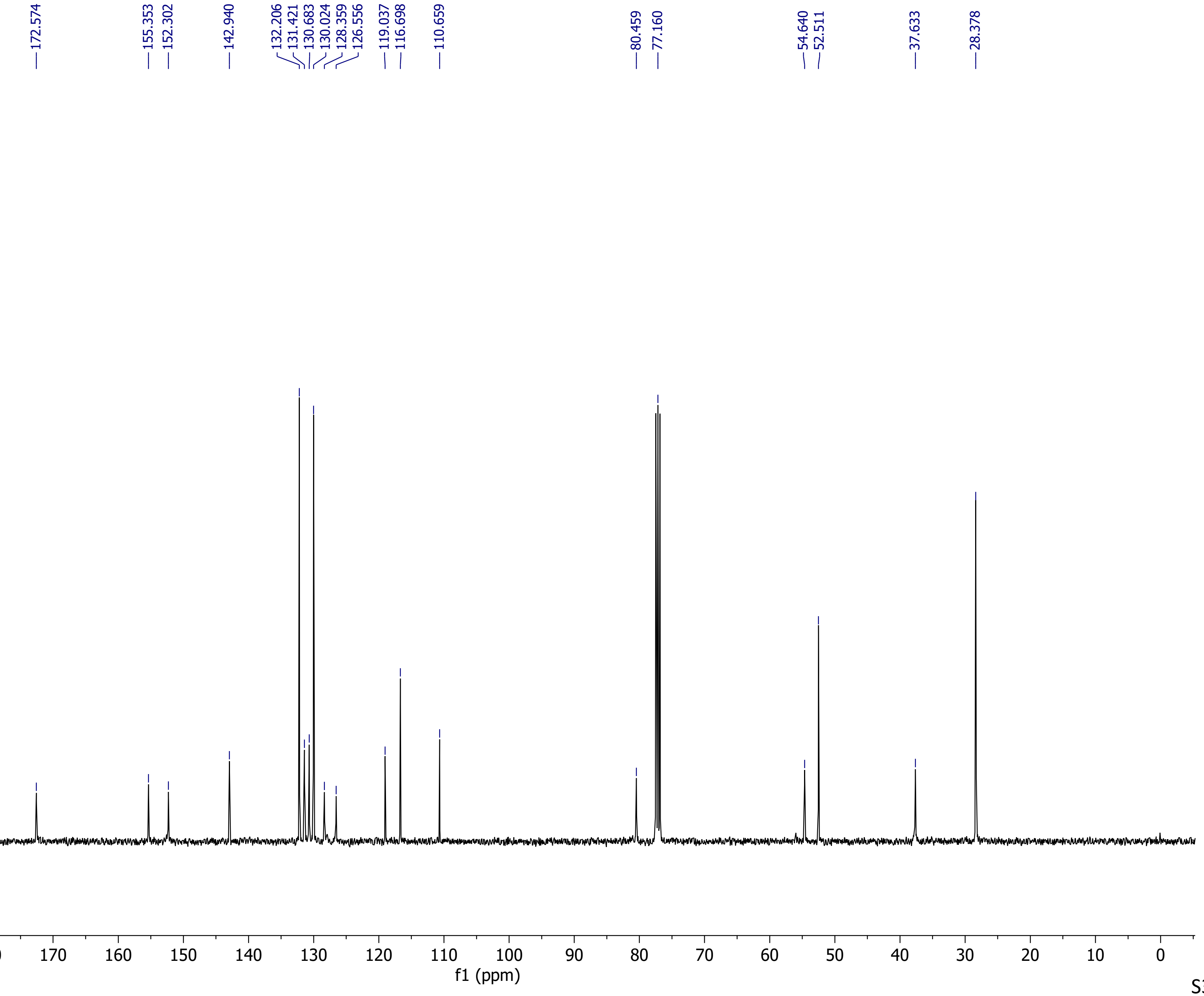

^{1}H NMR (400 MHz, $CDCl_3$)

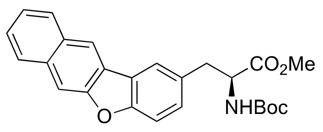

10a

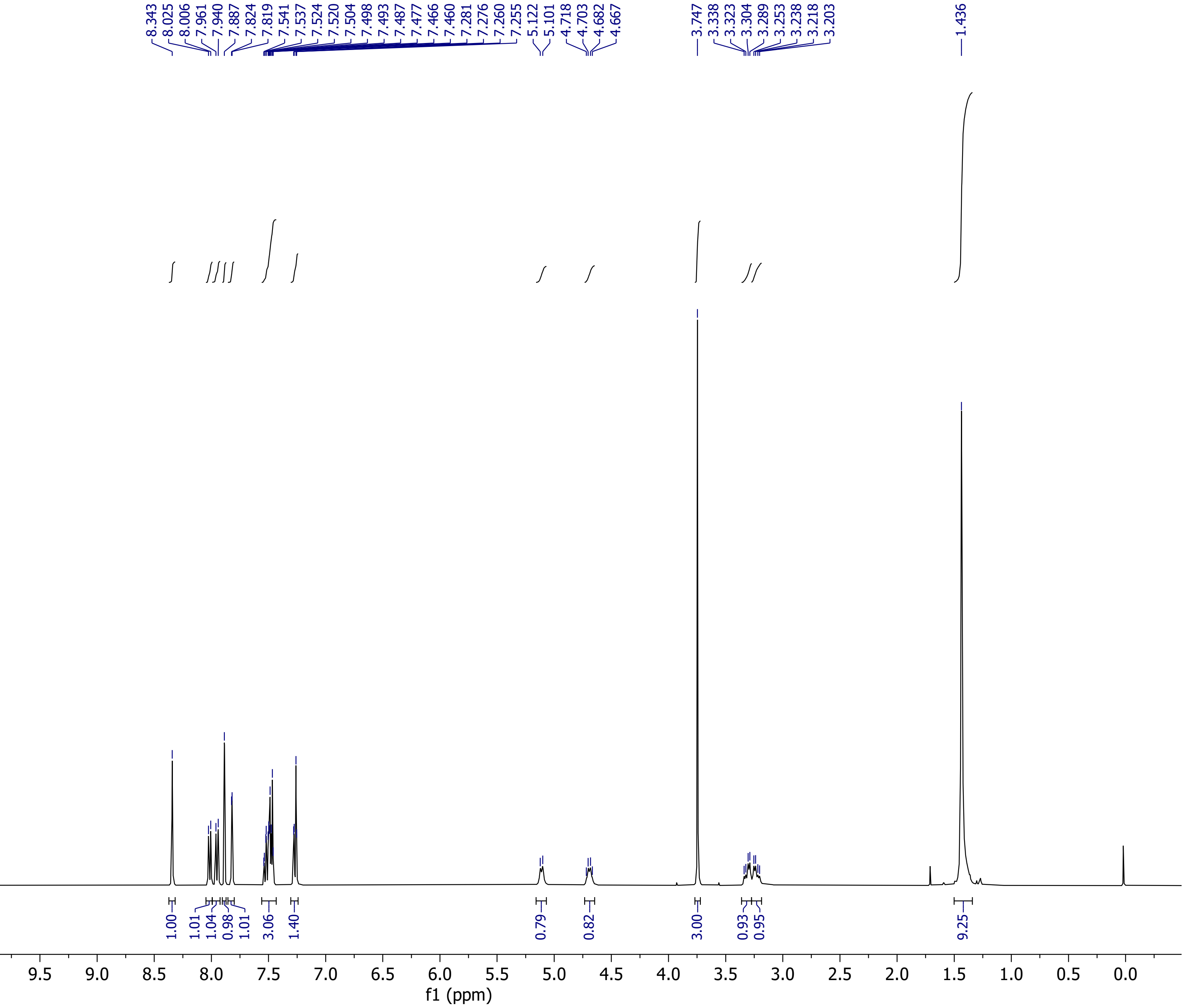

$^{13}C\{^1H\}$ NMR (101 MHz, $CDCl_3$)

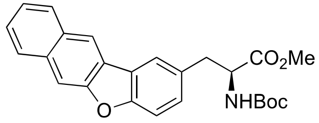

10a

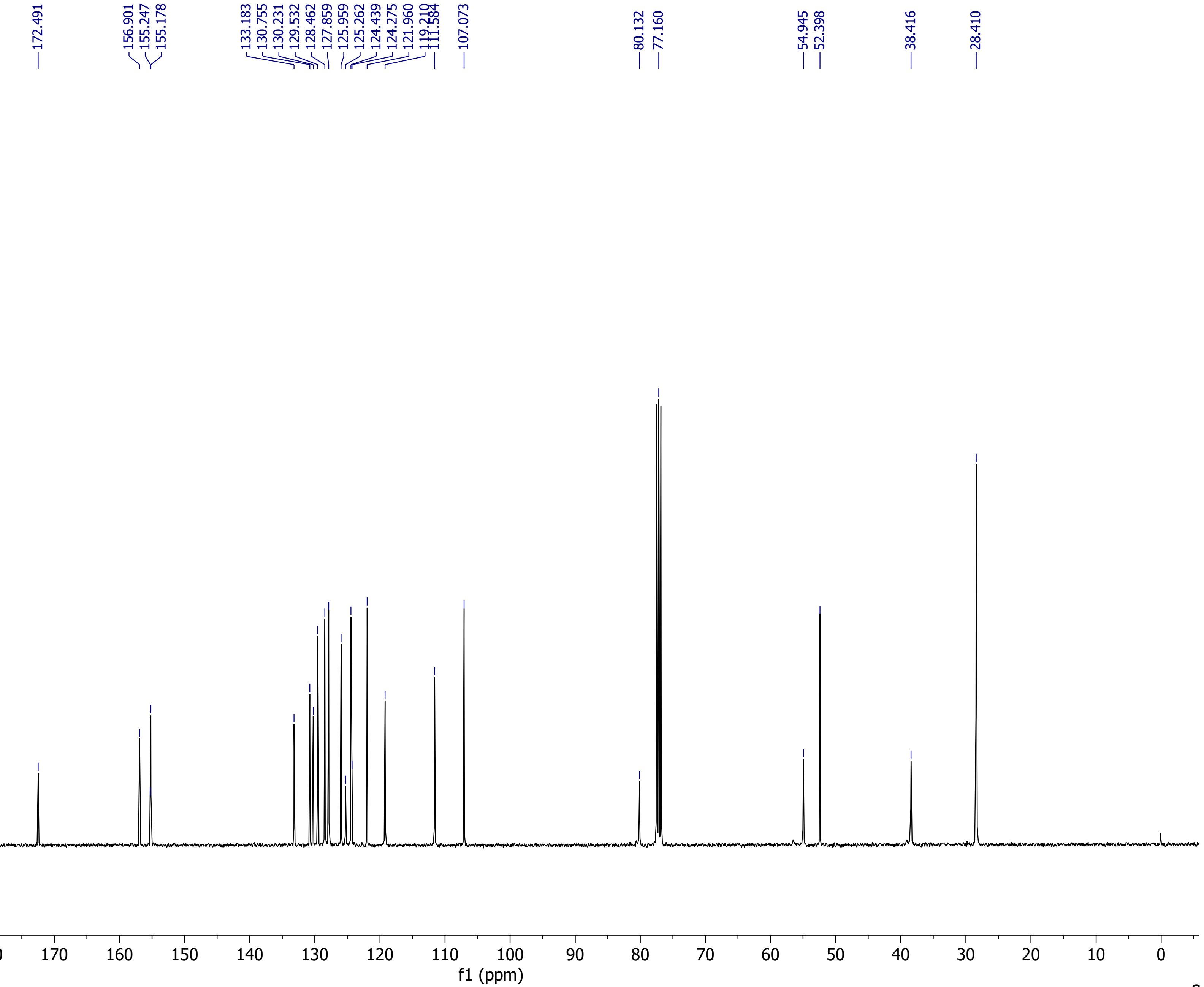

^{1}H NMR (400 MHz, CDCl$_3$)

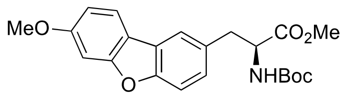

10b

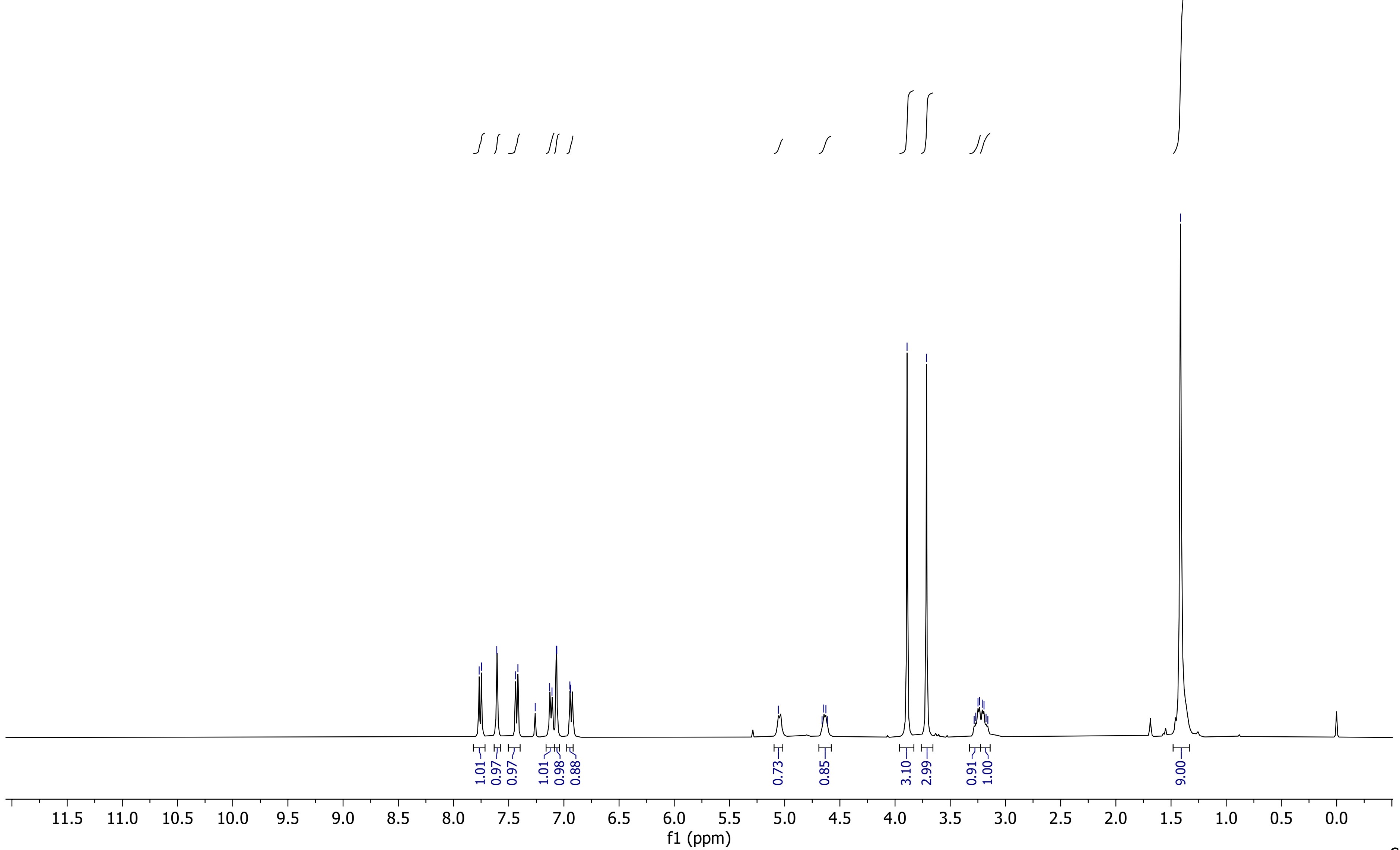

$^{13}C\{^1H\}$ NMR (101 MHz, $CDCl_3$)

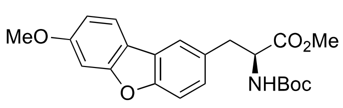

10b

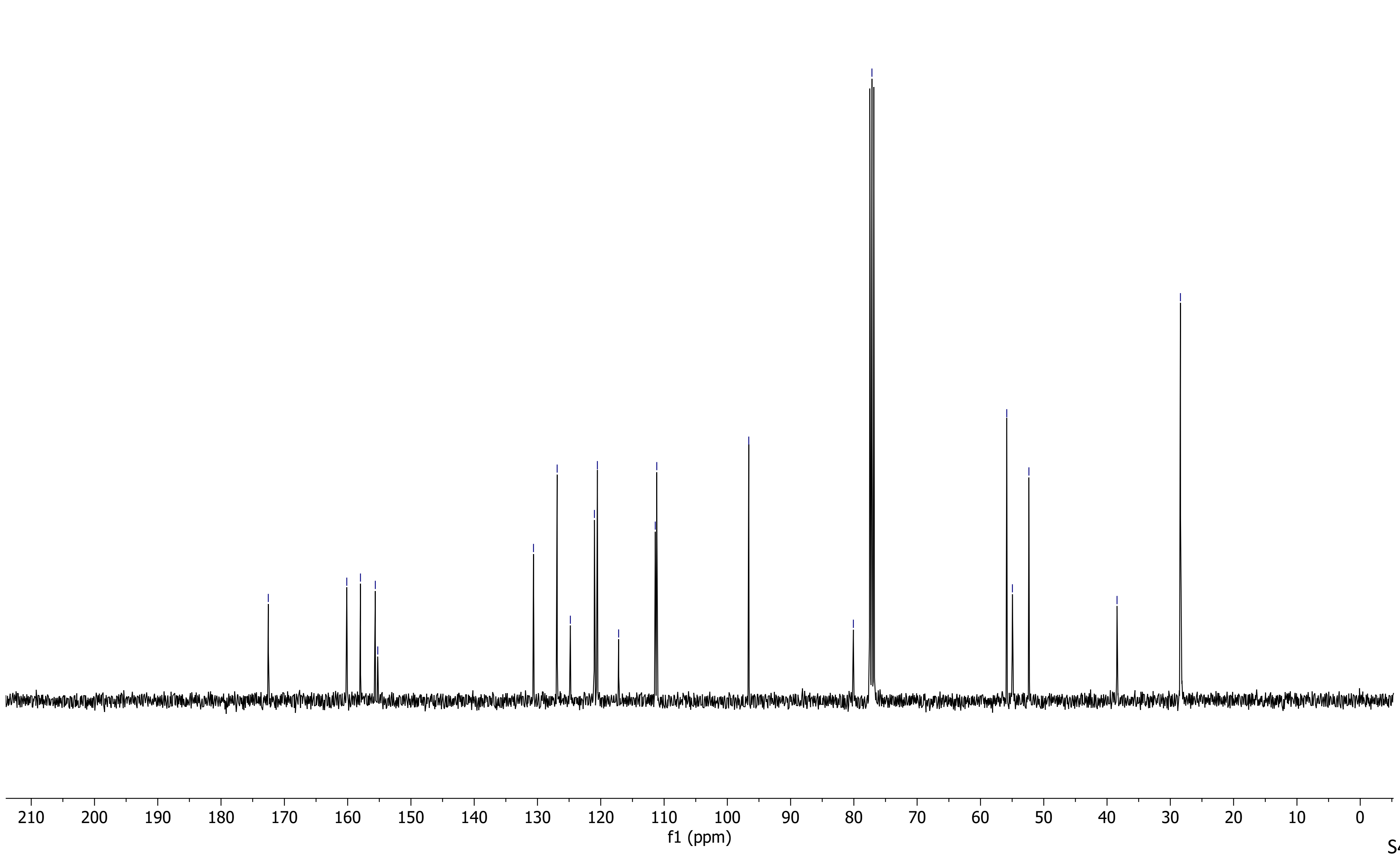

^{1}H NMR (400 MHz, $CDCl_3$)

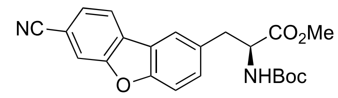

10c

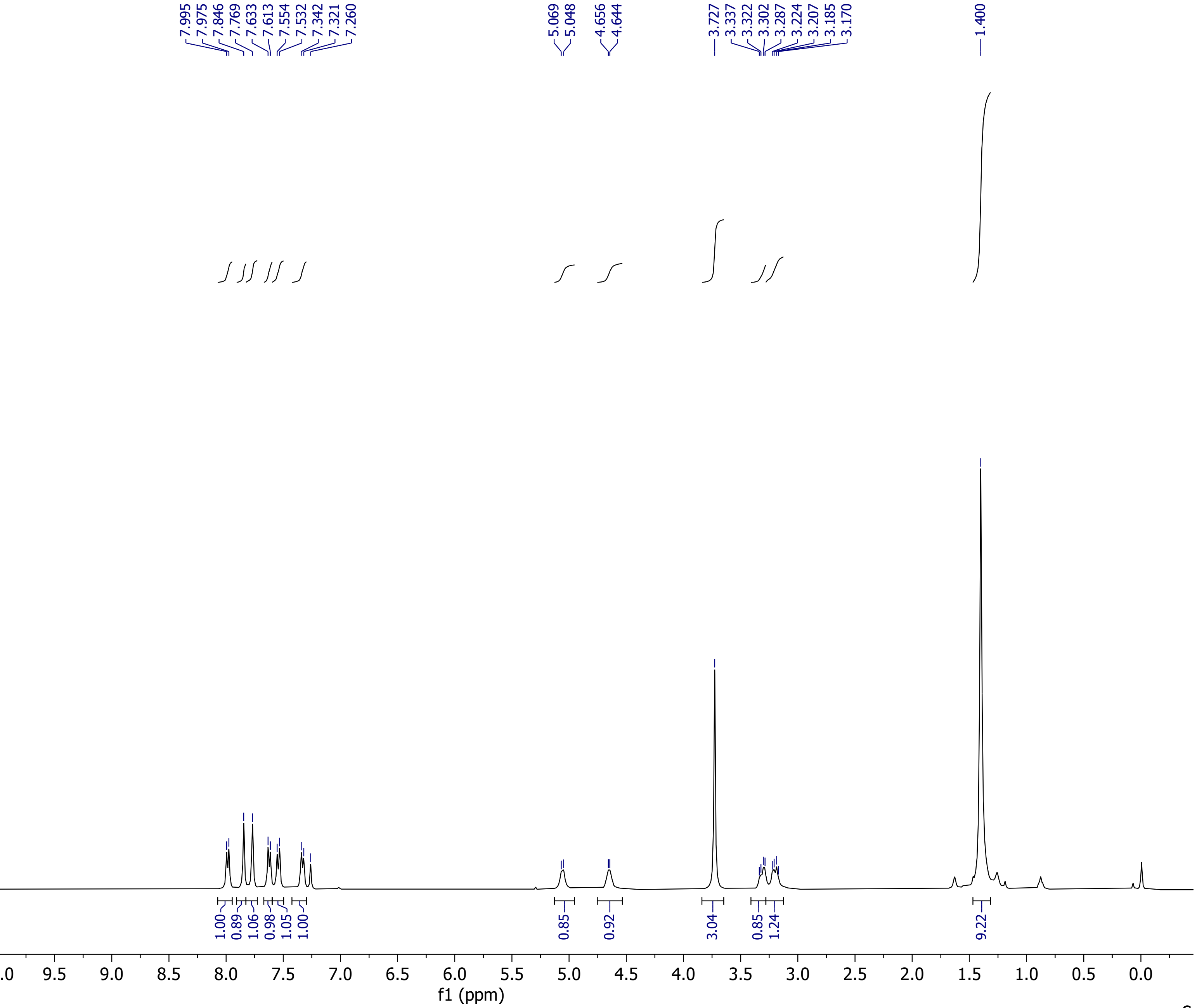

$^{13}C\{^{1}H\}$ NMR (101 MHz, $CDCl_3$)

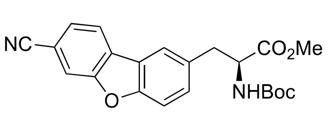

10c

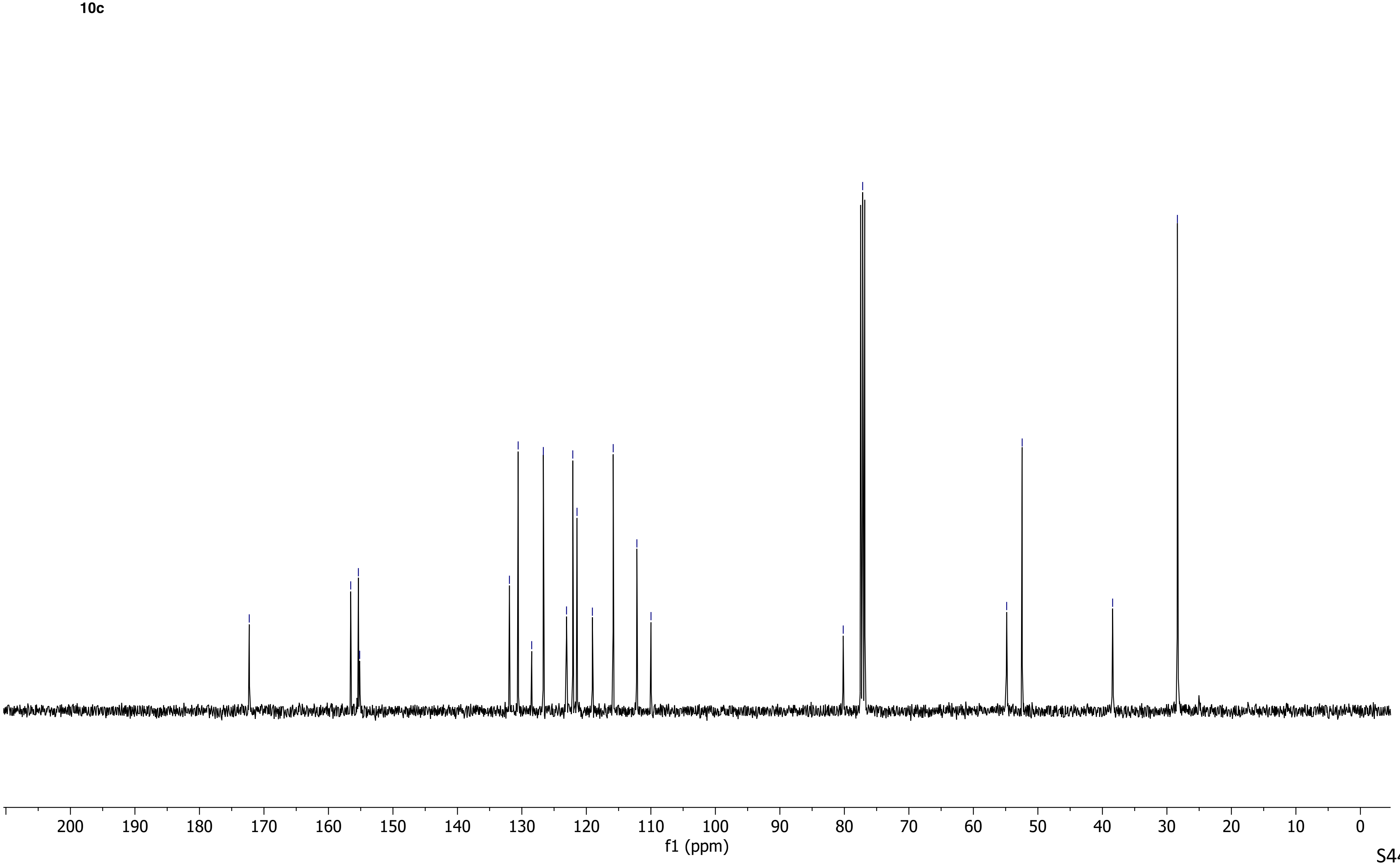

^{1}H NMR (400 MHz, $CDCl_3$)

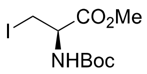

12

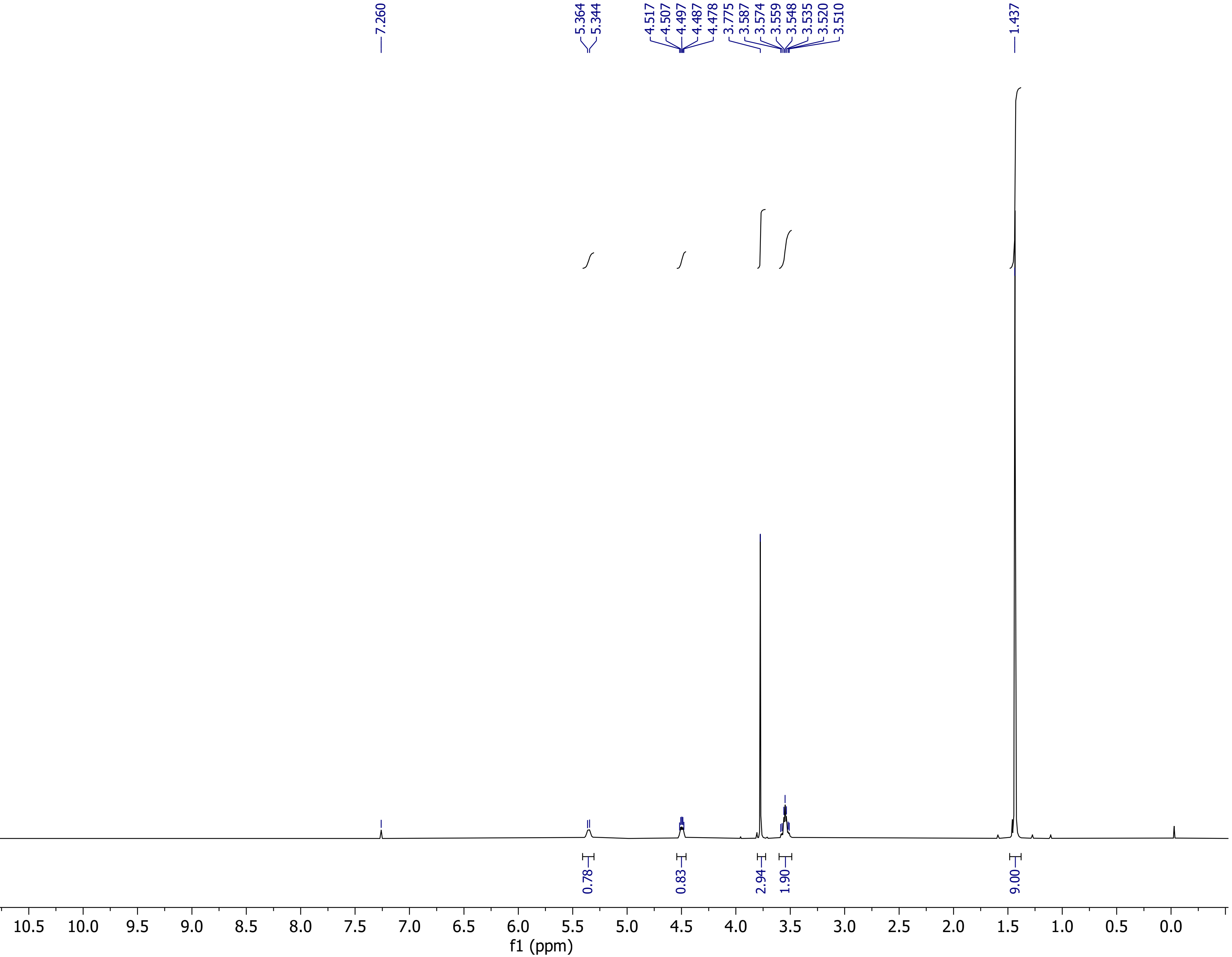

$^{13}C\{^{1}H\}$ NMR (101 MHz, $CDCl_3$)

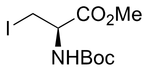

12

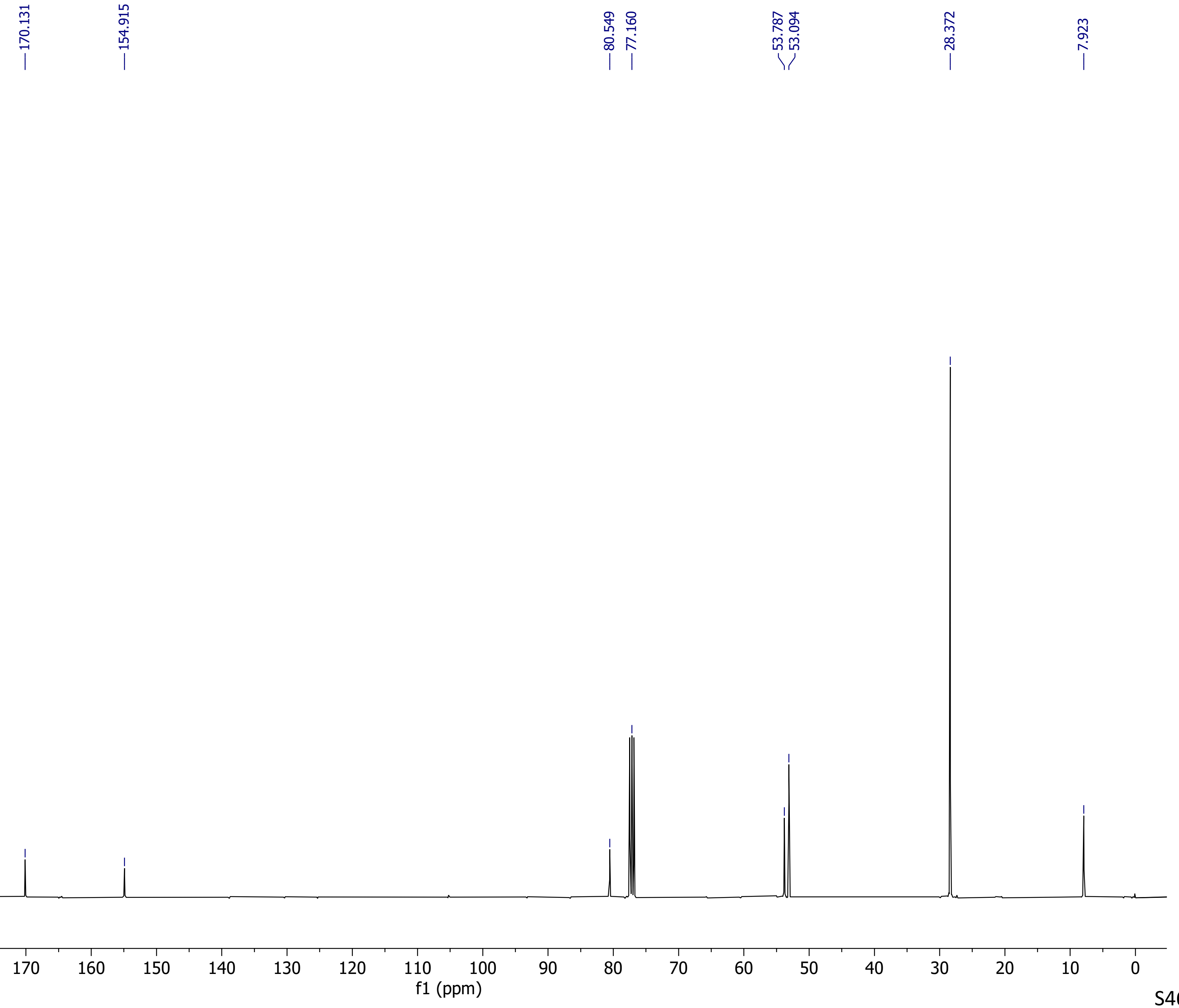

^{1}H NMR (400 MHz, $CDCl_3$)

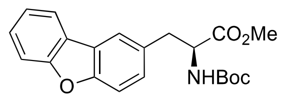

10d

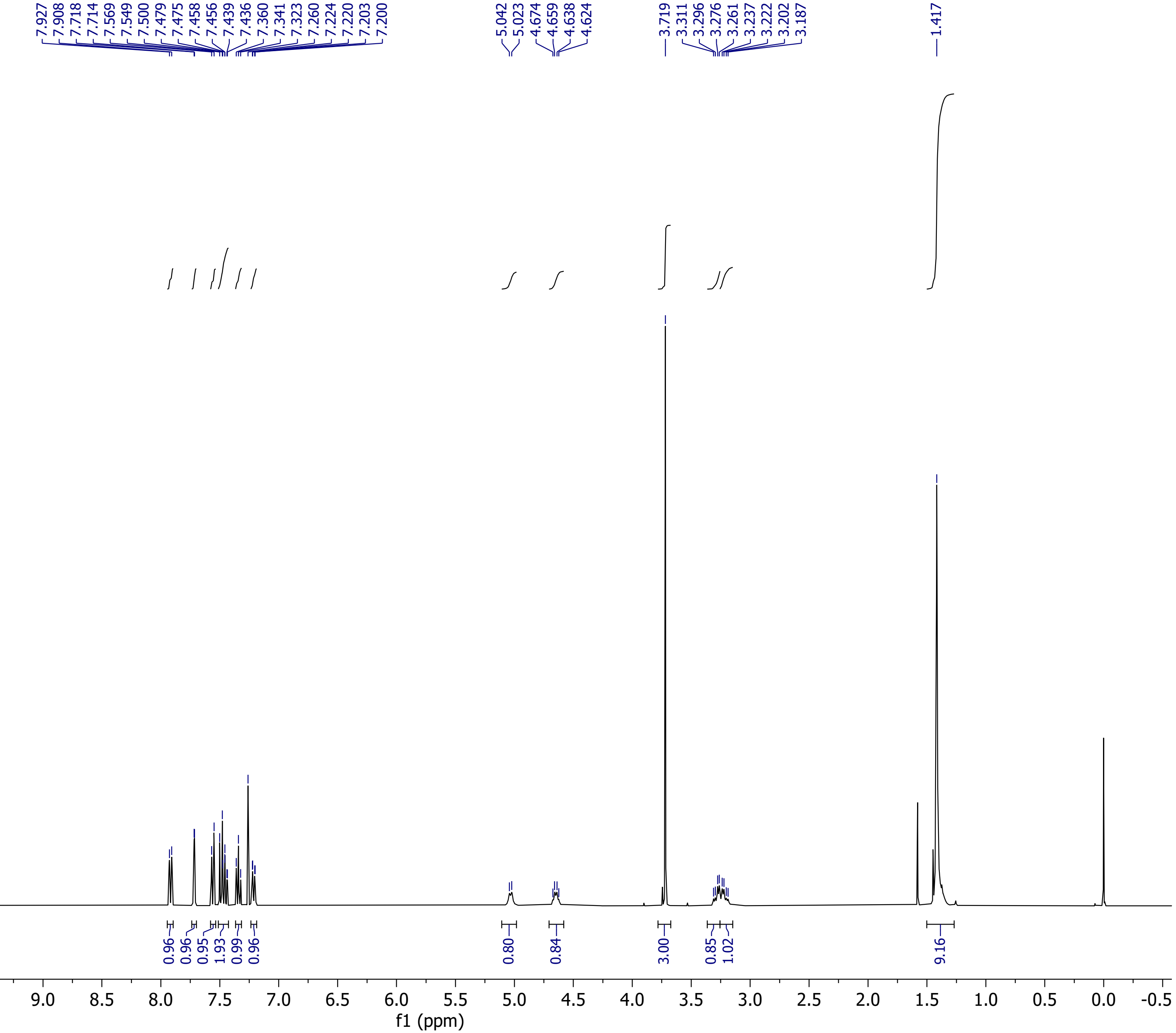

$^{13}C\{^1H\}$ NMR (101 MHz, $CDCl_3$)

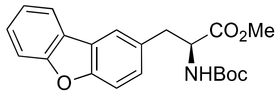

10d

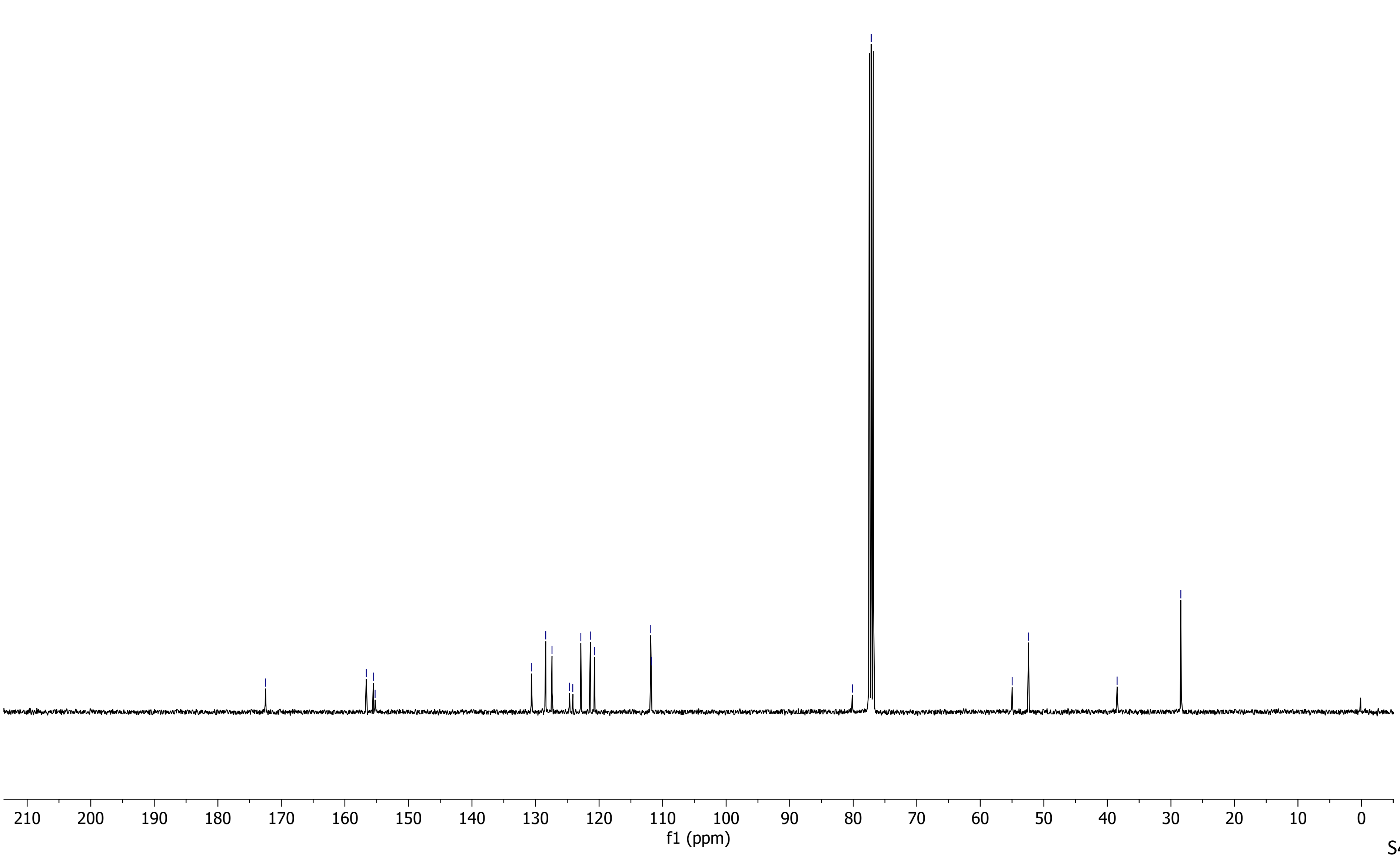

^{1}H NMR (400 MHz, $CDCl_3$)

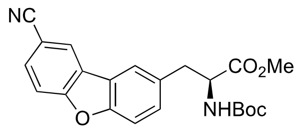

10e

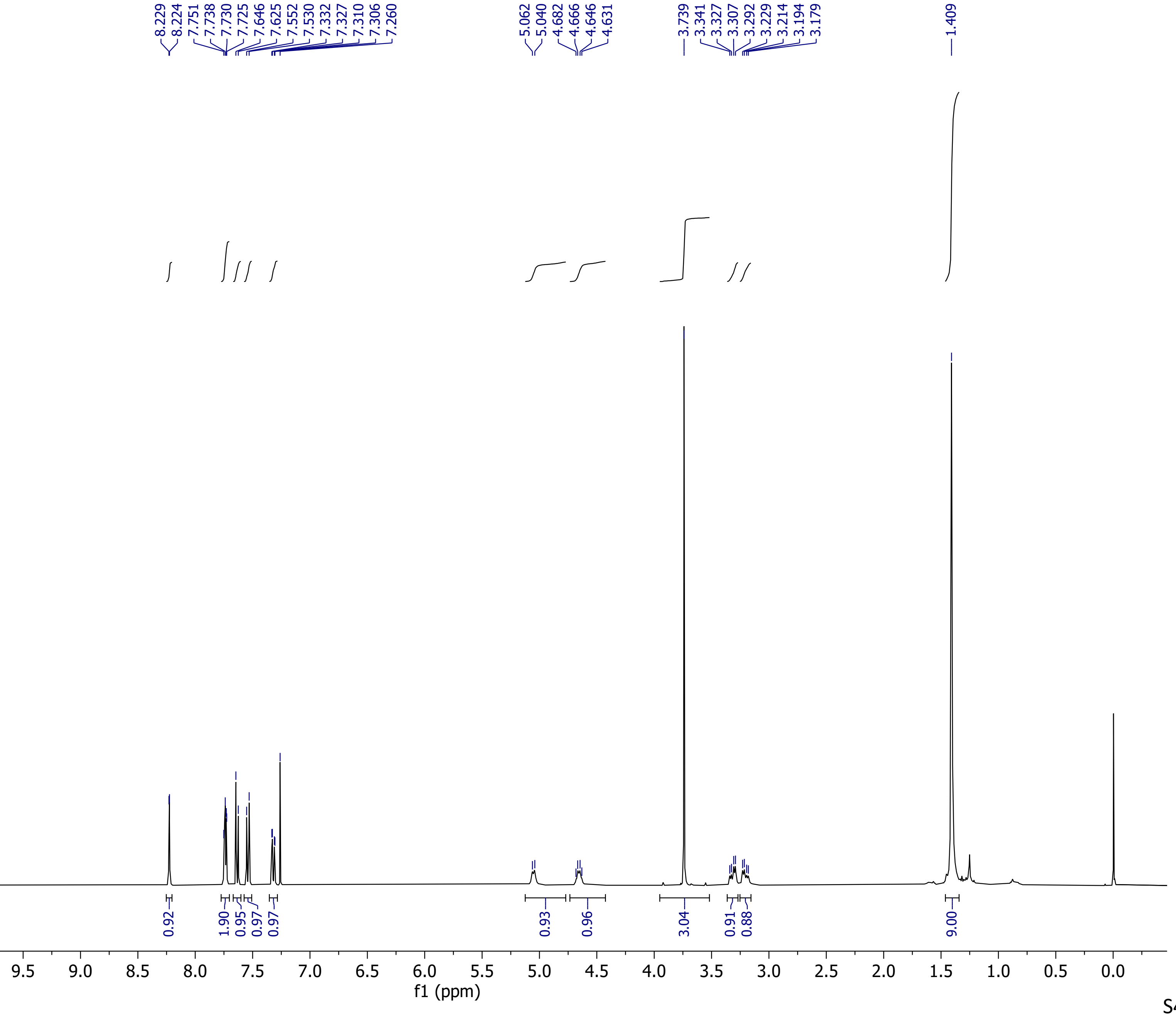

$^{13}C\{^{1}H\}$ NMR (101 MHz, $CDCl_3$)

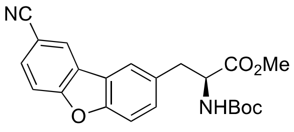

10e

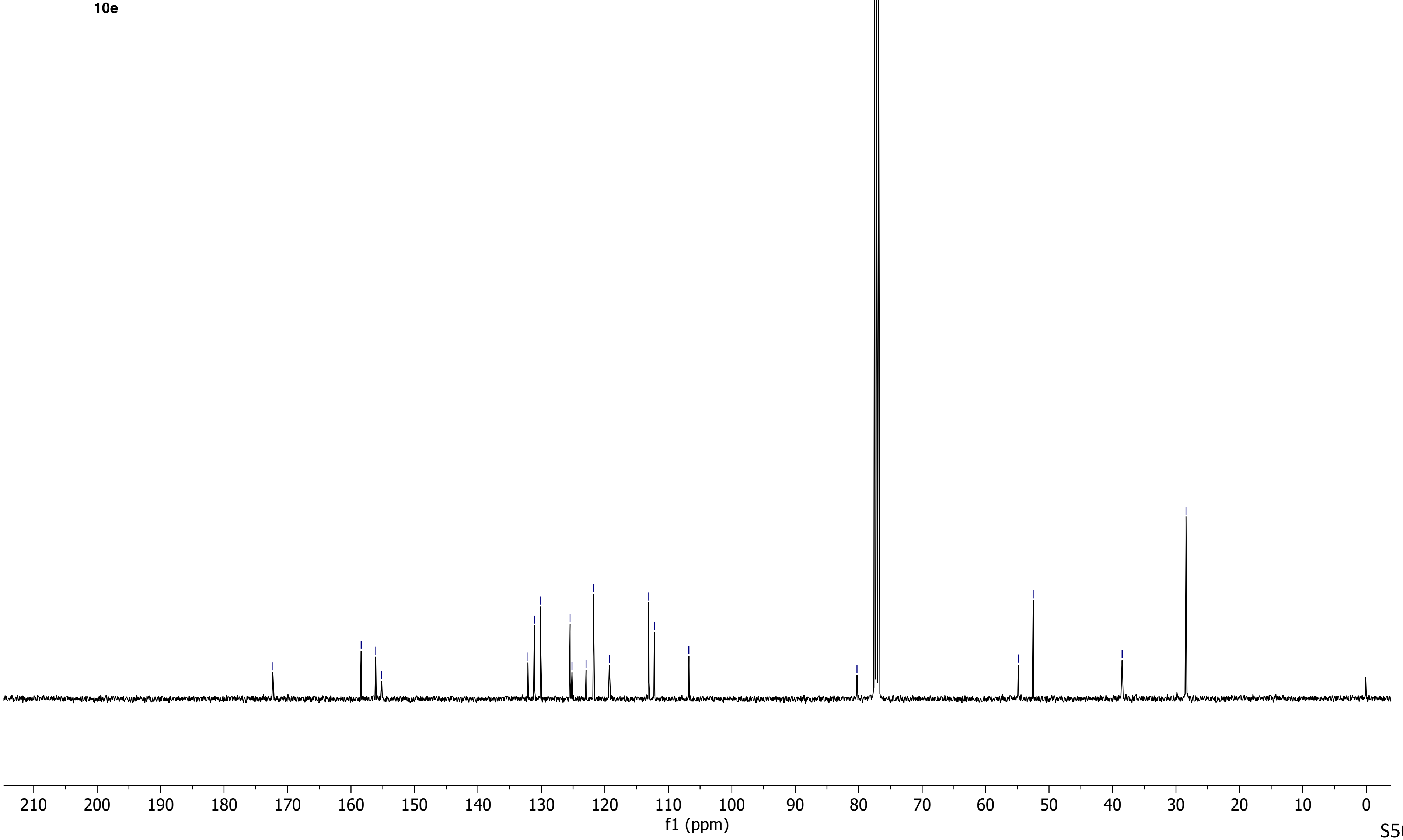

^{1}H NMR (400 MHz, CD_3OD)

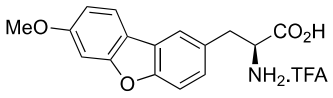

14

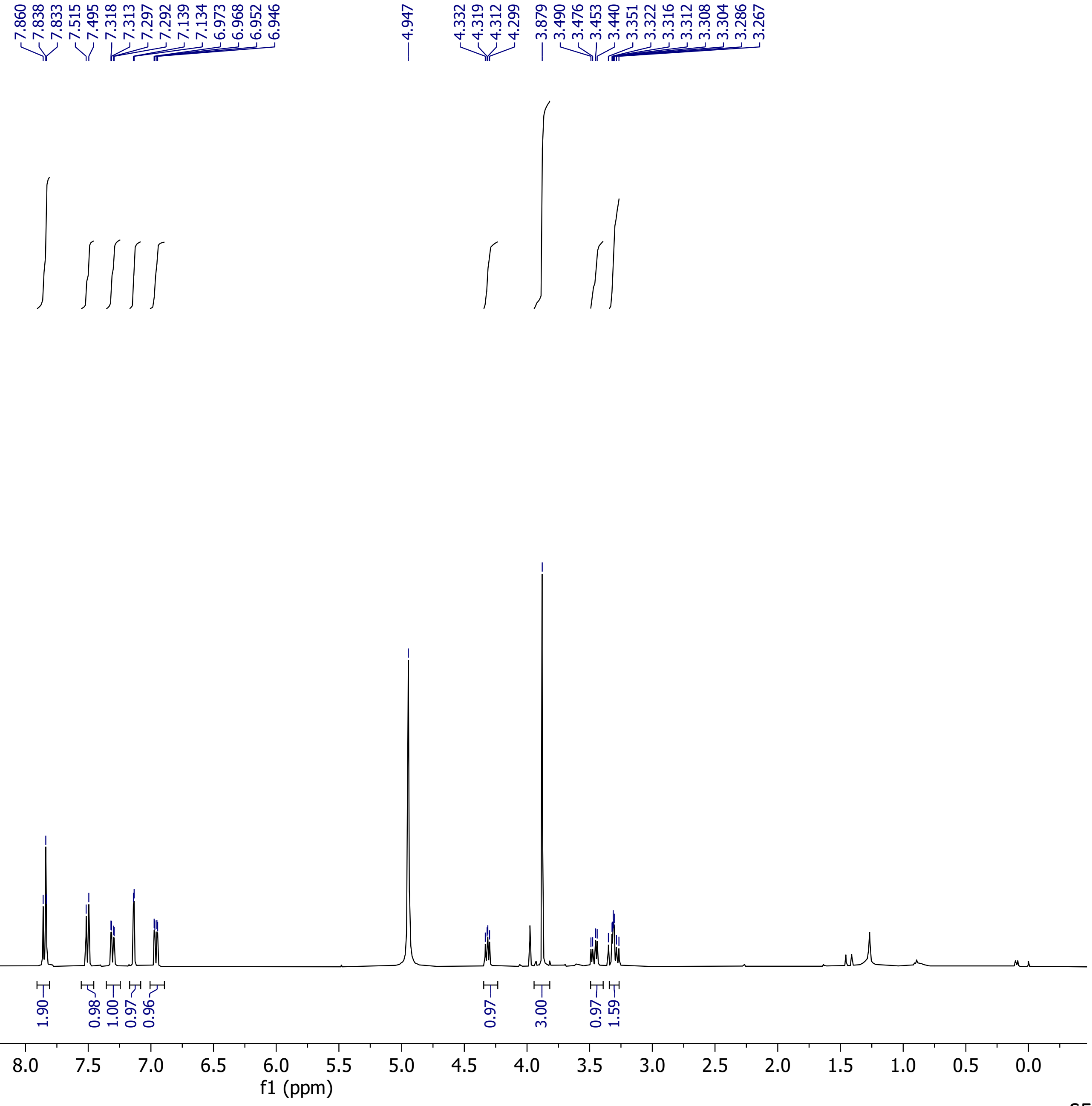

$^{13}C\{^{1}H\}$ NMR (101 MHz, CD_3OD)

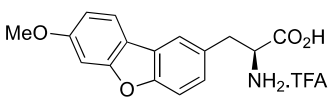

14

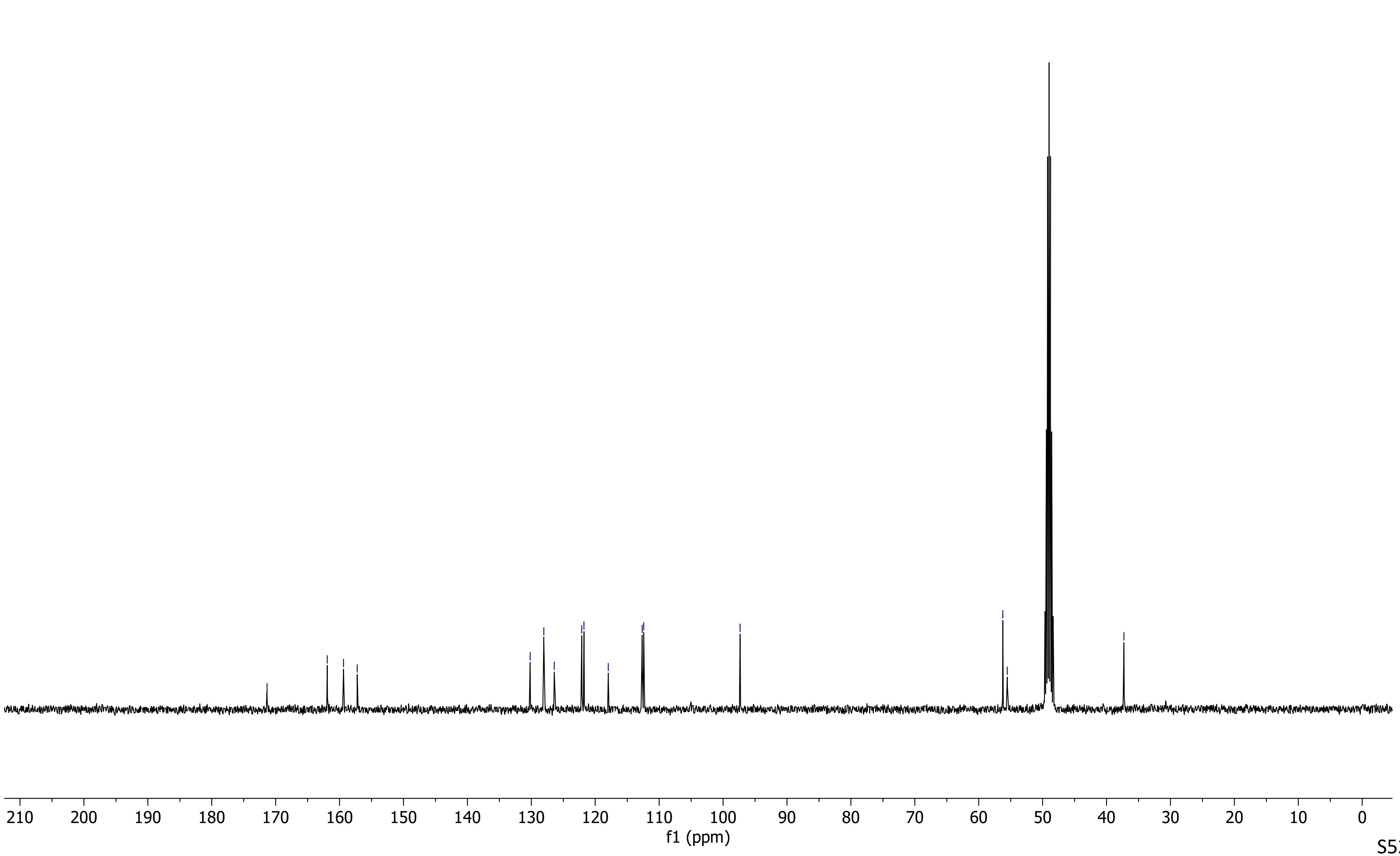

Supplement: Supplementary file 1 — ol5c00433_si_001.pdf [file ol5c00433_si_001.pdf]
